# Multidrug efflux pumps of *Pseudomonas aeruginosa* show selectivity for their natural substrates

**DOI:** 10.3389/fmicb.2024.1512472

**Published:** 2025-01-09

**Authors:** Léna Mazza, Alexandre Bory, Alexandre Luscher, Joachim Kloehn, Jean-Luc Wolfender, Christian van Delden, Thilo Köhler

**Affiliations:** ^1^Service of Infectious Diseases, Geneva University Hospitals, Geneva, Switzerland; ^2^Department of Microbiology and Molecular Medicine, University of Geneva, Geneva, Switzerland; ^3^School of Pharmaceutical Sciences, University of Geneva, Geneva, Switzerland; ^4^Institute of Pharmaceutical Sciences of Western Switzerland, University of Geneva, Geneva, Switzerland

**Keywords:** *Pseudomonas aeruginosa*, antibiotic resistance, multidrug efflux, metabolomics, natural substrates

## Abstract

Antibiotic-resistant Gram-negative bacteria are an increasing threat to human health. Strategies to restore antibiotic efficacy include targeting multidrug efflux pumps by competitive efflux pump inhibitors. These could be derived from natural substrates of these efflux systems. In this work, we aimed to elucidate the natural substrates of the clinically relevant Mex efflux pumps of *Pseudomonas aeruginosa* by an untargeted metabolomic approach. We constructed a PA14 mutant, genetically deleted in the major multidrug efflux pumps MexAB-OprM, MexCD-OprJ, MexXY-OprM, and MexEF-OprN and expressed in this mutant each efflux pump individually from an inducible promoter. Comparative analysis of the exo-metabolomes identified 210 features that were more abundant in the supernatant of efflux pump overexpressors compared to the pump-deficient mutant. Most of the identified features were efflux pump specific, while only a few were shared among several Mex pumps. We identified by-products of secondary metabolites as well as signaling molecules. Supernatants of the pump-deficient mutant also showed decreased accumulation of fatty acids, including long chain homoserine lactone quorum sensing molecules. Our data suggests that Mex efflux pumps of *P. aeruginosa* appear to have dedicated roles in extruding signaling molecules, metabolic by-products, as well as oxidized fatty acids. These findings represent an interesting starting point for the development of competitive efflux pump inhibitors.

## Introduction

Multidrug resistant (MDR) bacteria are a major threat to human health and new strategies to prevent and treat infections with MDR pathogens are urgently needed. Efflux pump (EP) inhibition, allowing potentiation of existing antibiotics, is a promising strategy (Venter et al., 2015; Zgurskaya et al., 2021; Sharma et al., 2019). EPs are major determinants of the MDR phenotype in Gram-negative pathogens. EPs transporting clinically relevant antibiotics in Gram-negative bacteria belong to the so-called Resistance Nodulation-Division (RND) family (Alav et al., 2021; Henderson et al., 2021; Dreier and Ruggerone, 2015; Köhler et al., 1999; Du et al., 2018). These form a tripartite transmembrane spanning complex composed of an inner membrane pump (IMP) protein, an outer membrane channel protein (OMP) and a membrane fusion protein (MFP) (Du et al., 2018; Neuberger et al., 2018).

*Pseudomonas aeruginosa* is a Gram-negative opportunistic pathogen, responsible for both acute and chronic infections, mainly in immunocompromised hosts, burn patients, as well as in cystic fibrosis (*CF*) patients (Reynolds and Kollef, 2021). *P. aeruginosa* is part of the WHO ESKAPE pathogen list for which novel antibiotics or alternative strategies are urgently needed (Miller and Arias, 2024). The bacterium is well known for its broad-spectrum multidrug efflux pumps, which are able to extrude a wide variety of antimicrobials and antiseptics (Botelho et al., 2019; Lister et al., 2009). Four Mex EPs of the RND family play a major role in the resistance phenotype of clinical isolates: the constitutively expressed MexAB-OprM pump as well as the inducible MexXY-OprM, MexCD-OprJ and MexEF-OprN efflux systems (Gotoh et al., 1998; Masuda et al., 2000; Verchère et al., 2015; Sanz-García et al., 2022)*. P. aeruginosa* further encodes eight RND type efflux pumps (Adamiak et al., 2021) of which MexVW and MexMN were shown to be able to extrude quinolone and beta-lactam antibiotics, respectively (Li et al., 2003). The remaining RND type EPs, including MexPQ*-*OpmE, do not transport antibiotics but have been shown to be involved in modulation of the quorum sensing response (MexJK) (Amieva et al., 2022) and extrusion of phenazines (MexGHI-OpmD) (Sakhtah et al., 2016).

Apart from their ability to transport antimicrobials, the role of Mex EPs in *P. aeruginosa* is not fully understood. Recent phylogenetic analysis revealed that Gram-negative EPs of the RND family did not evolve in response to clinical use of antibiotics but rather are ancient physiological traits (Teelucksingh et al., 2020; Zwama et al., 2019). Indeed, EP-encoding genes in *E. coli* do not appear to be accessory genes, as is often the case with antibiotic resistance elements, but are components of the intrinsic antibiotic resistome (Teelucksingh et al., 2020). These findings therefore raise the question of natural functions and substrates of RND EPs. Several hypotheses can be made, among them their use for transporting signaling molecules, secondary metabolites or self-produced toxic waste in their natural setting. Previous works hypothesized that Mex EPs of *P. aeruginosa* are involved in quorum sensing modulation (Amieva et al., 2022; Maseda et al., 2004; Alcalde-Rico et al., 2018; Alcalde-Rico et al., 2020; Evans et al., 1998; Pearson et al., 1999).

EPs represent an interesting target for drug potentiators. Indeed, blocking EPs has been shown to potentiate the efficacy of already existing antimicrobials by increasing their accumulation in the cytosol (Sharma et al., 2019; Pagès et al., 2005; Mehla et al., 2021; Pagès et al., 2005; Lamut et al., 2019; Lomovskaya et al., 2001). Several molecules have been identified, among them the most widely used compound phenylarginyl-*β*-naphtylamide (PAβN), which decreases minimal inhibitory concentrations (MIC) for several antibiotic classes and in a variety of Gram-negative pathogens but revealed being toxic to eukaryotic cells (Renau et al., 2003; Duffey et al., 2024). Another approach would be to use the natural substrates of the EPs as competitive inhibitors, or by chemically modifying them to administer them concomitantly with existing drugs, allowing the accumulation of antibiotics in the bacterial cytosol (Laborda et al., 2021; Nakayama et al., 2003; Tambat et al., 2022).

In this respect, we used untargeted metabolomics to analyze and compare the culture supernatants, termed afterwards exo-metabolome of wild type *P. aeruginosa* strain PA14, as well as a mutant (PA14Δ4mex) lacking the four clinically relevant EPs, in which we overexpressed individually Mex EPs from an inducible promoter. We found that EPs impact the fatty acid content of the exo-metabolome, and identified signaling molecules, byproducts of biosynthetic pathways or toxic metabolites as natural substrates of *P. aeruginosa* EPs. Importantly, we conclude that EPs show specificity toward their natural substrates. These novel insights should fuel the development of selective or broad-spectrum EP-inhibitors to combat problematic Gram-negative pathogens.

## Results

### Gene expression, antibiotic resistance, and growth profile of the PA14Δmex4 mutant and efflux pump overexpressors

To perform an unbiased, comparative analysis of potential natural EPs substrates, we constructed a mutant strain, PA14Δ4mex, genetically deleted in the four major clinically relevant Mex EPs: *mexAB-oprM*, *mexCD-oprJ*, *mexXY-oprM*, and *mexEF-oprN*. Since four major efflux pumps were deleted in the PA14Δ4mex mutant, we wondered whether this would affect the expression of other Eps introducing a bias in the metabolomics analysis. We thus performed an RNAseq analysis comparing PA14Δ4mex to PA14. Seventy-two genes were down-regulated (log_2_FC ≤ −1.0) and thirty-eight up-regulated (log_2_FC ≥ 1.0) in the PA14Δ4mex strain compared to the PA14 wild type (Supplementary Figure S1). Interestingly, three genes, *PA14_36010*, *PA14_36,020* and *PA14_36,030*, forming an operon and coding for a potential transmembrane lipid transport system homologous to the LetALetB system of *E. coli* (Isom et al., 2020), were the most overexpressed genes in PA14Δ4mex compared to PA14 (Supplementary Figure S1). The phospholipid desaturase gene *desB* was also overexpressed in PA14Δ4mex (Pezzoni et al., 2022; Supplementary Table S1). Importantly, expression of genes belonging to the remaining eight RND EP systems (*mexMN*, *mexJK*, *mexGHI-opmD*, *triABC*, *mexVW*, *mexPQ-opmE*, *muxABC-opmB* and *czcCBA*) was not affected (Supplementary Table S1), confirming that further analyses should not be influenced by a compensatory overexpression of other EPs in the PA14Δ4mex strain background. To explore the natural substrate profile of RND EPs in *P. aeruginosa*, we cloned the operons of the four major drug efflux pumps *mexAB-oprM, mexEF-oprN, mexCD-oprJ, mexXY-oprM* as well as the operon of the *mexPQ-opmE* pump, not involved in antibiotic efflux, into the expression vector pSRKGm under the control of the inducible *lac* promoter (Khan et al., 2008). The obtained constructs were then expressed individually in the EP deficient strain PA14Δ4mex. To verify the functionality of the overexpressed EPs, we assessed the susceptibility of these strains against their antibiotic substrates. As expected, the EP-deficient strain PA14Δ4mex was 2 to 64-fold more susceptible to the tested antibiotics than the PA14 wild type strain in standard MIC determination (Table 1; Clinical and Laboratory Standards Institute, 2012). Overexpression of the individual EPs correlated with previously established antibiotic substrate profiles, with MexAB-OprM showing the largest spectrum of antibiotic substrates (*n* = 9), followed by MexXY-OprM (*n* = 7), MexCD-OprJ (*n* = 6) and MexEF-OprN (*n* = 4) (Table 1). The MexPQ-OpmE pump did not contribute to antibiotic efflux (*n* = 0). We conclude from these data that the overexpressed pumps are functional and confer the expected antibiotic substrate profile.

**Table 1:** Antibiotic substrate profile of efflux pump overexpressing strains.

Antibiotic	MICs (μg/mL)						
	PA14 + pSRKGm	PA14Δ4mex + pSRKGm	PA14Δ4mex + pSRK-mexABM	PA14Δ4mex + pSRK-mexCDJ	PA14Δ4mex + pSRK-mexEFN	PA14Δ4mex + pSRK-mexXYM	PA14Δ4mex + pSRK-mexPQE
Aztreonam	4	0.125	**>16**	0.125	0.125	0.125	0.125
Carbenicillin	>8	0.5	**>8**	0.5	0.5	0.5	0.5
Ampicillin	256	8	**>256**	16	8	**128**	8
Azithromycin	128	2	**16**	**128**	1	**>128**	1
Tetracyclin	4	0.25	**>32**	**2**	0.5	**32**	0.25
Ciprofloxacin	0.06	0.004	**0.5**	**0.06**	**0.03**	**0.5**	0.004
Chloramphenicol	64	1	**>64**	**8**	**64**	**8**	0.5
Sulfamethoxazole	128	8	**>128**	**32**	**>128**	**16**	8
Trimethoprime	>128	2	**>128**	**128**	**>128**	**128**	2
Antibiotic substrates (*n*)	NA	NA	(9)	(6)	(4)	(7)	(0)

MIC, minimal inhibitory concentration; NA, not applicable. MIC values of EP-overexpressors showing a 4 fold change compared to vector control are shown in bold.

**Figure 1:**
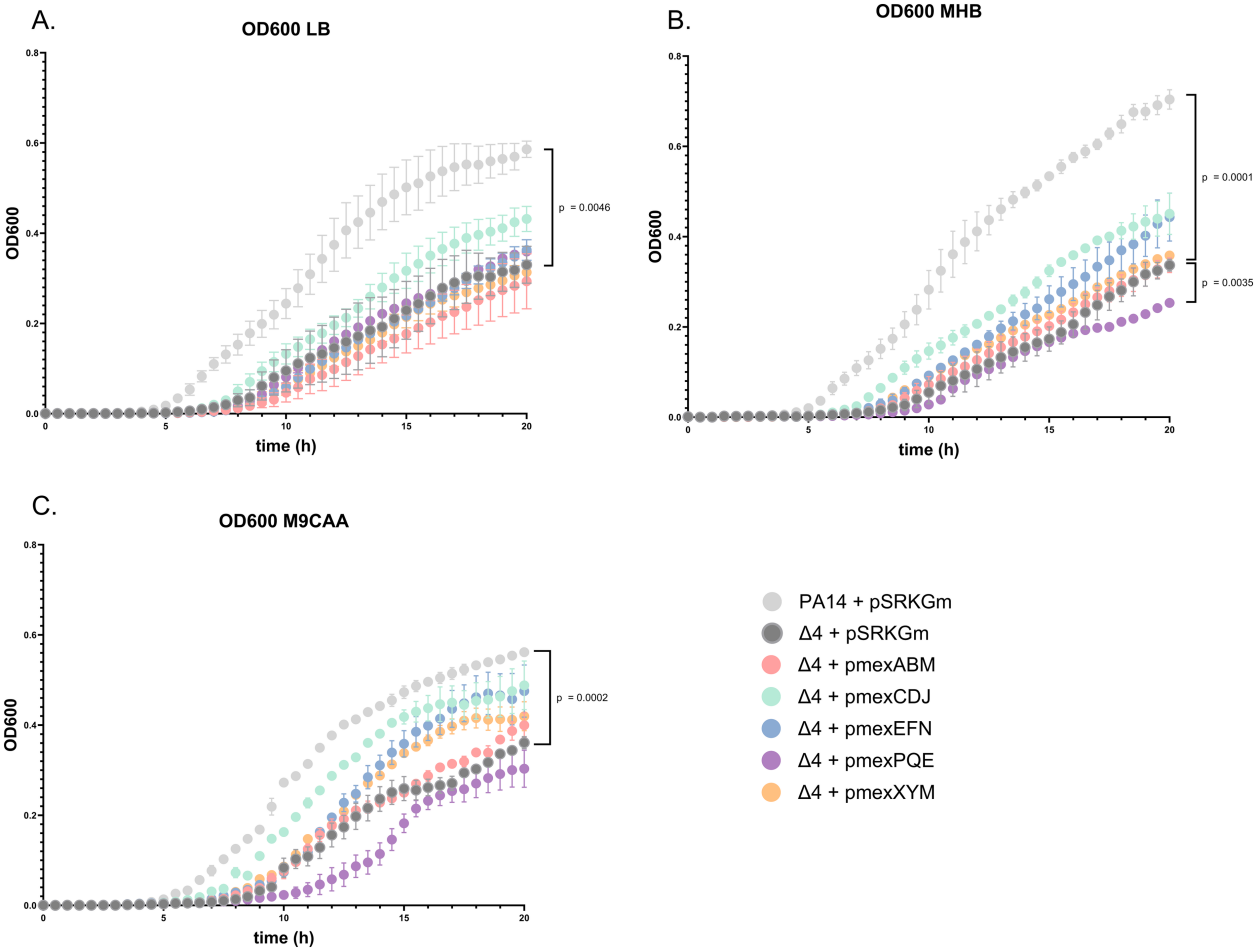
Growth of EP-overexpressing strains. Growth was measured in triplicates in microtiter plates in the following media: **(A)** Lysogeny Broth (LB). **(B)** Mueller-Hinton Broth (MHB). **(C)** M9 medium supplemented with casamino acids (M9CAA). Data represent the mean of triplicate determinations. *p*-values were determined using an unpaired *T*-test comparing cell density (OD600) values at 20 h of growth.

We then compared the growth of the PA14Δ4mex mutant with the individual EP-overexpressors and the PA14 wild type in different media (LB, MHB and M9CAA). A prolonged lag phase was observed in the EP mutants compared to the PA14 wild type (Figure 1). The slope of exponential growth phase, however, was similar between the two strains irrespective of the growth medium. We hypothesize the growth delay to be linked to the accumulation of toxic metabolites in the cytosol or the membranes. Moreover, PA14Δ4mex reached lower final cell densities (OD600) compared to PA14. Individual overexpression of the five EPs did not lead to a complete growth restoration, suggesting a simultaneous requirement of several EPs to achieve optimal growth. Interestingly, overexpression of MexCD-OprJ improved growth mainly in MHB and in the iron-limited M9CAA medium. Surprisingly, the overexpression of the constitutively expressed MexAB-OprM did not restore growth, while overexpression of MexPQ-OpmE further delayed growth in both MHB and M9CAA (Figure 1).

### Deletion of the four clinically relevant EPs changes the global content of the exo-metabolomes

**Figure 2:**
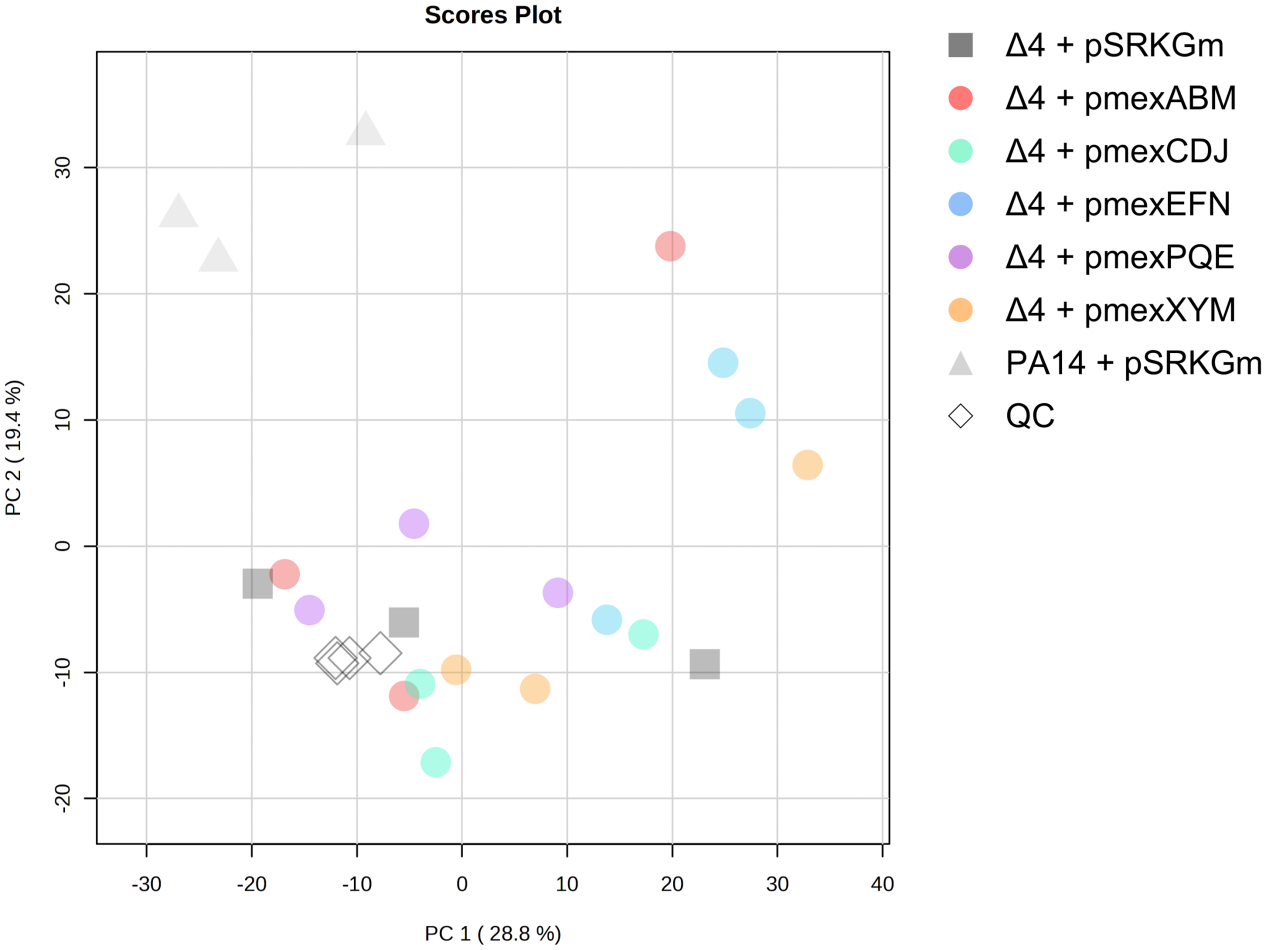
Score plot of the principal component analysis (PCA) of exo-metabolomes. Culture supernatants were prepared in three biological replicates. Quality control (QC) samples were prepared by pooling all samples. The analysis was performed on the online platform metaboanalyst.com.

Following the hypothesis that toxic metabolites accumulate in the bacteria and lead to a delayed growth upon EPs deletion, we aimed at elucidating the natural role and substrates of Mex EPs in *P. aeruginosa*. Using first untargeted Ultra-High Performance Liquid Chromatography–Mass Spectrometry (UHPLC–MS) we globally analyzed the metabolite content of PA14 wild type, PA14Δ4mex and EP-overexpressors supernatants. As we used sequential ethyl acetate and dichloromethane liquid–liquid extractions, the analysis focused primarily on the apolar portion of the exo-metabolomes. A total of 2,579 features (m/z @ RT), defined as a mass (m) over a charge (z) at a specific retention time (RT), were detected in the analyzed supernatants. Principal Component Analysis (PCA) clearly discriminated the exo-metabolomes of PA14 wild type compared to the PA14Δ4mex based strains (Figure 2). This points out the involvement of Mex EPs in the metabolite composition of culture supernatants and suggests that the expression of a single EP on a plasmid is not sufficient to restore the overall content of PA14 supernatant, similar to what was observed in the growth assay of the EP-overexpressors (Figure 1).

### EPs modulate the exo-metabolome fatty acid content

We first compared the exo-metabolomes of PA14 and PA14Δ4mex, to gain insight into the global effect of EPs on metabolite transport. For this and all further analyses we selected only features presenting a log_2_fold change (FC) of ≥1 or ≤ −1 and a *p*-value ≤0.05 (Student t-test), termed hereafter “features of interest.” Applying these selection criteria, we submitted the features identified in the UHPLC–MS analysis to Sirius and MS2Query annotation tools (Dührkop et al., 2019; de Jonge et al., 2023). Sirius provided the most comprehensive compound prediction compared to MS2Query (91.5% vs. 32.1% annotated features, respectively). To obtain a taxonomically informed prediction all annotations were re-ranked using Tima-R (Rutz et al., 2019).

**Figure 3:**
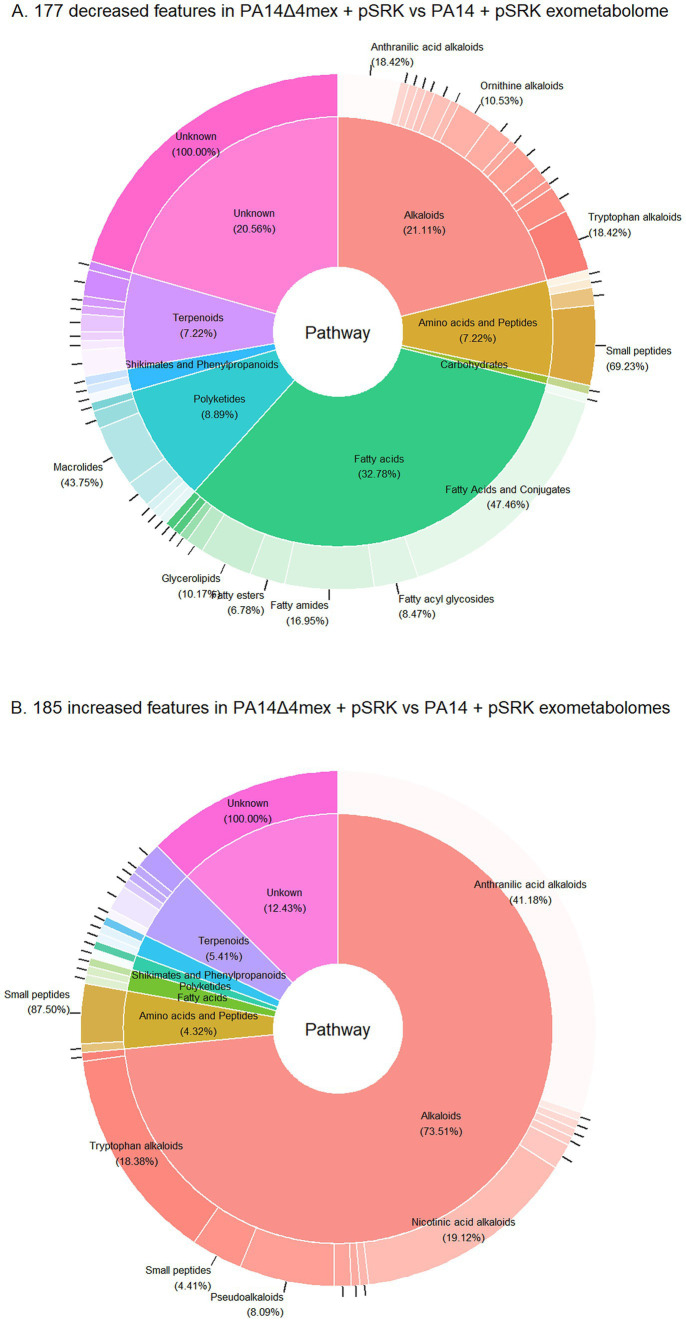
Features of interest (log_2_FC of ≥ 1 or ≤ −1 and a *p*-value ≤ 0.05) of PA14Δ4mex versus PA14 exo-metabolomes. Classification by NPClassifier (Kim et al., 2021): NPC pathways (inner ring) and superclasses (outer ring). **(A)** Decreased features of interest. **(B)** Increased features of interest.

The identified features could be attributed to seven different chemical classes according to NPClassifier (Kim et al., 2021): alkaloids, fatty acids, polyketides, amino acids and peptides, shikimates and phenylpropanoids and terpenoids (Figure 3). According to the UHPLC–MS analysis, a total of 362 features of interest (14% of total features) were changed in abundance in PA14∆4mex compared to PA14 (177 decreased, 185 increased) (Supplementary Table S2). The most represented class among the decreased features was fatty acids (32.78%), followed by alkaloids (21.11%) (Figure 3A). To validate the decreased abundance of fatty acids in the PA14Δ4mex exo-metabolome, we performed a semi-targeted gas chromatography–mass spectrometry (GC–MS) analysis. Importantly, while the UHPLC–MS analysis was performed directly on the extracts resuspended in DMSO, the GC–MS analysis was performed on the same extracts that were derivatized, limiting the analysis to metabolites possessing either a free acid function or esters. We only considered metabolites presenting a match factor of more than 70% when searched against NIST mass spectrometry library (Babushok et al., 2007; Zenkevich et al., 2009; Qu et al., 2021). Using this threshold, a total of 116 features were tentatively identified, among them 11 features (9.5% of total) (Supplementary Table S3) showed altered abundance (8 decreased, 3 increased) (log_2_FC of ≥1 or ≤ −1 and a *p*-value ≤0.05) between PA14Δ4mex and PA14 exo-metabolomes. All GC–MS decreased features were predicted to be fatty acids (Supplementary Table S3). When comparing the two analyses (UHPLC–MS, GC–MS), two predicted oxidized fatty acids were decreased in PA14Δ4mex supernatants by both techniques: hydroxy-octanoic acid and 3-hydroxy-decanoic acid, further confirming the role of EPs in fatty acid transport (Supplementary Figure S2). We next identified features that showed increased abundance in PA14Δ4mex when compared to PA14 (Figure 3B). Alkaloids, specifically pyridine quinazoline, pyocyanin and other phenazines, were the most prominent features increased in the exo-metabolome of PA14Δ4mex and identified by both UHPLC–MS (Supplementary Figure S3) and GC–MS (Supplementary Tables S2, S3). This was corroborated by measuring spectrophotometrically the concentration of pyocyanin, which was significantly increased (*p*-value = 0.0062) in PA14Δ4mex supernatants compared to the one of PA14 (Supplementary Figure S4). This may be explained by a precursor of pyocyanin being a substrate of EPs, accumulating in the PA14Δ4mex cytosol and leading to increased synthesis and diffusion of pyocyanin into the external medium.

Overall, the deletion of the four EPs mainly impacted fatty acids, specifically oxidized fatty acids, which were less abundant in the exo-metabolome of the EPs deficient mutant, as observed by both UHPLC–MS and GC–MS. This suggests that Mex EPs might be involved in export of (oxidized) fatty acids that accumulate in the inner membrane thereby affecting growth of the EP deficient mutant.

### Selectivity of EPs for their potential natural substrates

Given the functional assessment of the cloned Mex EPs via antibiotic transport (Table 1), we next compared the exo-metabolome of each individual EP-overexpressing strain to the one of PA14Δ4mex. We suspected that secondary metabolites, signaling molecules or toxic waste generated during growth could be natural substrates. The UHPLC–MS analysis revealed that the PA14Δ4mex exo-metabolome, differed from the EPs overexpressors with respect to the number of overrepresented features (*n*) (log_2_FC ≥ 1, *p*-value ≤0.05): MexAB-OprM (*n* = 62), MexCD-OprJ (*n* = 49), MexEF-OprN (*n* = 36), MexXY-OprM (*n* = 83) and MexPQ-OpmE (*n* = 52) (Supplementary Figure S5; Supplementary Table S2). In the GC–MS analysis, only a reduced number of features were increased upon EP overexpression (*n*): MexAB-OprM (*n* = 1), MexCD-OprJ (*n* = 3), MexEF-OprN (*n* = 2), and MexPQ-OpmE (*n* = 3) (Supplementary Table S3). No chemical class was specifically attributable to an individual EP. However, the metabolites content of the MexCD-OprJ and MexXY-OprM overexpressor supernatants showed a smaller proportion of fatty acids (8.2 and 2.4% respectively) than those of MexAB-OprM (14.5%), MexPQ-OpmE (17.3%) and MexEF-OprN (22.2%) (Supplementary Figure S5).

**Figure 4:**
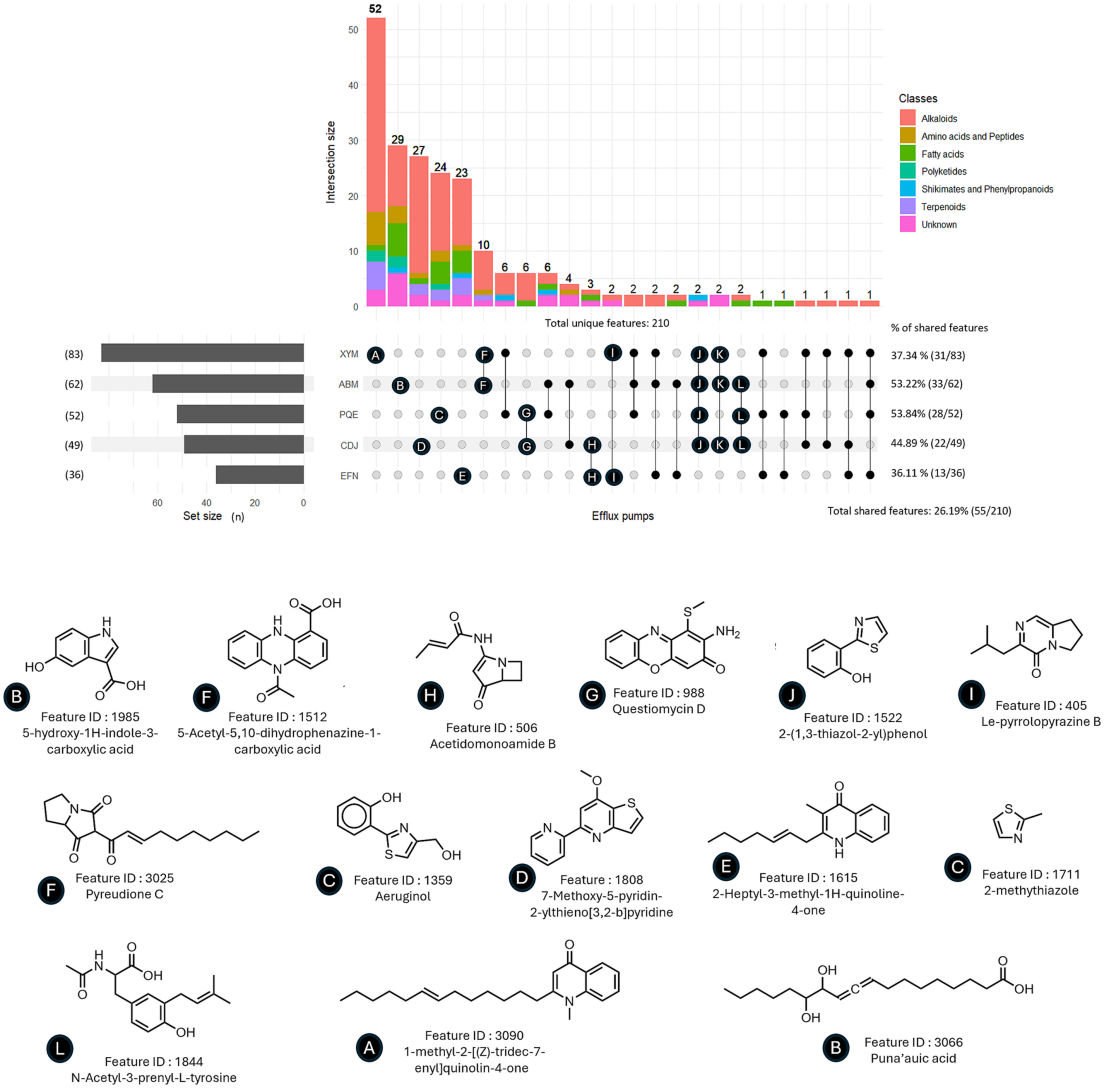
Distribution of features of interest increased upon EP-overexpression in exo-metabolomes and examples of predicted molecular structures. Features of interest are defined by a log_2_FC ≥ 1 and a *p*-value ≤0.05 when compared to the PA14Δ4mex exo-metabolome. Statistical analysis was performed with standard student *T*-test. Predicted structures were given by the Sirius annotation tool (Dührkop et al., 2019).

We then compared the sets of UHPLC–MS features with increased abundance in supernatants of a specific EP with each other (Figure 4; Supplementary Figure S6). Overall, most features of interest were specific to a particular exo-metabolome with only 55 out of 210 (26.2%) overlapping between two or more sets, pointing toward specific endogenous substrates transported by each pump. The percentage of features shared with at least one other EP-overexpressor varied from 36.11% (MexEF-OprN) to 53.84% (MexPQ-OpmE). MexXY-OprM and MexAB-OprM had the largest number of overlapping features (*n* = 10), most of them belonging to the alkaloid class. None of the increased features were shared by all five overexpressors exo-metabolomes (Figure 4). Potential substrates predicted by Sirius (Dührkop et al., 2019) (as presented in the examples in Figure 4) belong to a wide range of molecular patterns. While some of them are known as *P. aeruginosa* metabolites, the majority are unknown yet. The predicted feature Pyreudione C, an alkaloid produced by *P. fluorescens*, was increased in strains overexpressing MexAB-OprM and MexXY-OprM (Klapper et al., 2018; Klapper et al., 2016). Acetidomonoamine B, a *P. aeruginosa* metabolite derived from a quorum-sensing regulated non-ribosomal peptide synthetase (NRPS) and involved in biofilm formation, was found among the increased features of both MexCD-OprJ and MexEF-OprN overexpressors (Ernst et al., 2022). Questiomycin A, a phenazine antibacterial metabolite known to be produced by *P. chlororaphis*, was predicted to be increased in MexPQ-OpmE and MexCD-OprJ exo-metabolomes (Guo et al., 2022). Finally, some quinolones were found to be increased in MexXY-OprM. Other features overrepresented in several EP-overexpressing strains include indoles, phenazines and thiazole containing molecules (Figure 4). While the natural substrates seem to be EP-specific, we could not find clear molecular patterns explaining these specificities when comparing physico-chemical properties such as logP, polar surface area, molecular weight or total charge of the predicted metabolites (data not shown). Altogether, these results indicate a rather specific natural substrate profile for Mex EPs in *P. aeruginosa.*

### Signaling molecules, metabolic byproducts, and secondary metabolites are potential natural substrates of Mex efflux pumps

**Figure 5:**
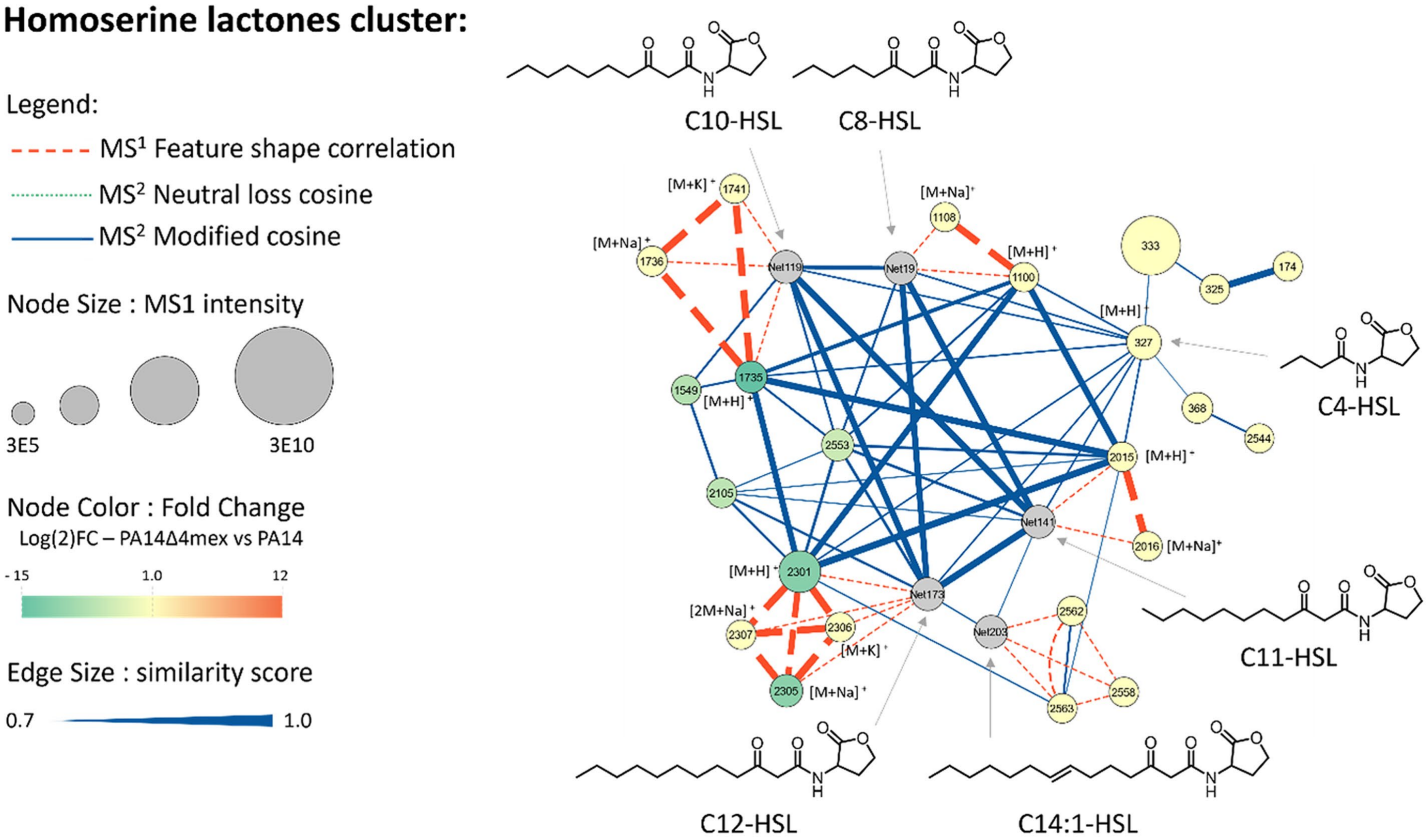
Metabolomic analysis and molecular networks, A cluster of homoserine lactone (HSL) features was generated in MzMine based on their spectral similarities, expressed as cosine scores, allowing grouping of structurally similar features. The identified features are represented as nodes (circles), showing their ID in the node centers together with the adduct when identified. The size of each node is proportional to the MS^1^ intensity of the feature. Nodes are colored based on their relative abundance in the PA14Δ4mex exo-metabolome compared to PA14 wild type exo-metabolome. Nodes are linked with different relationships and width’s edges are proportional to the similarity. Putative structures are based on SIRIUS annotations and C4-HSL was confirmed by injection of a commercially available standard.

Several of the overrepresented features in EP overexpressors were predicted by Sirius (Dührkop et al., 2019) to be well known signaling molecules and secondary metabolites. We employed commercially available standard molecules (Supplementary Table S4) to confirm the identity of several *P. aeruginosa* produced metabolites in our UHPLC–MS analysis. This included the siderophore pyochelin (Supplementary Table S4), homoserine lactones (Figure 5) and two alkylquinolines (AQs), the pseudomonas quinolone signal (PQS) and its precursor 4-hydroxy-2-heptylquinoline (HHQ) (Supplementary Table S4). The spectra and retention times of the predicted metabolites matched those of the corresponding authentic standards, validating their identity and highlighting the accuracy of the *in-silico* prediction tools. This approach enabled us to confidently predict the presence of these endogenous metabolites in the samples and to map their relative abundance onto the network, highlighting significant differences between the PA14Δ4mex and PA14 exo-metabolomes.

AQs was the most represented molecule family overall in the analyzed exo-metabolomes, including PA14Δ4mex. Among the fifty most represented features (based on average peak intensity in all supernatants pooled in the quality control sample), 24% were AQs (IDs: 1954, 2132, 2771, 2694, 2388, 2659, 2145, 1783, 2111, 2472, 2716, 2256) (Supplementary Table S5). *P. aeruginosa* produces more than fifty AQs with variable carbon chain lengths and hydroxyl substitutions (Lépine et al., 2004). In our analysis, several AQs were enriched in MexAB-OprM, MexPQ-OpmE and MexEF-OprN supernatants (Supplementary Table S2). This indicates that Mex EPs are possible routes for AQs export, thereby supporting previous reports (Lamarche and Déziel, 2011). However, the quorum sensing autoinducer PQS was neither enriched or depleted in any of the EP overexpressing supernatants. Another interesting metabolite found significantly increased in the supernatant of MexPQ-OpmE was predicted to be aeruginol (log_2_FC = 2.29, feature ID 1359), a byproduct of the siderophore pyochelin biosynthesis (Supplementary Figure S7). However, two features predicted to be similar to aeruginol were decreased in MexPQ-OpmE exo-metabolome. These were predicted to be 7-hydroxy-6-oxo-2,5-dihydro-1H-isoquinoline-3-carboxylic acid (ID 1452) and 5-hydroxy-6-methoxy-2-methylisoindole-1,3-dione (ID 1355) (Supplementary Figure S7; Supplementary Table S2). Although pyochelin was among the fifty most represented features of the combined (QC) exo-metabolomes (IDs: 1509, 1699, 1816, and 1372) (Supplementary Table S5), it was not clearly increased in any of the EPs overexpressing supernatants, suggesting that it is not transported by the five EPs analyzed here.

A total of twenty-three features were attributed to homoserine lactones (HSLs) (Supplementary Table S6), and six were depleted in PA14Δ4mex when compared to PA14 exo-metabolomes (Figure 5). Among these six depleted homoserine lactones, we found two with a C10 side chain (IDs 1735 and 2553), two with a C12 side chain (IDs 2301 and 2305) and two with a C13 side chain (IDs 1549 2105) (Supplementary Table S6). Interestingly, shorter chain HSLs such as C4-HSL and C8-HSL were not affected by the EPs deletion or overexpression. Hence long chain HSL signaling molecules seem to be favored substrates of Mex EP.

**Figure 6:**
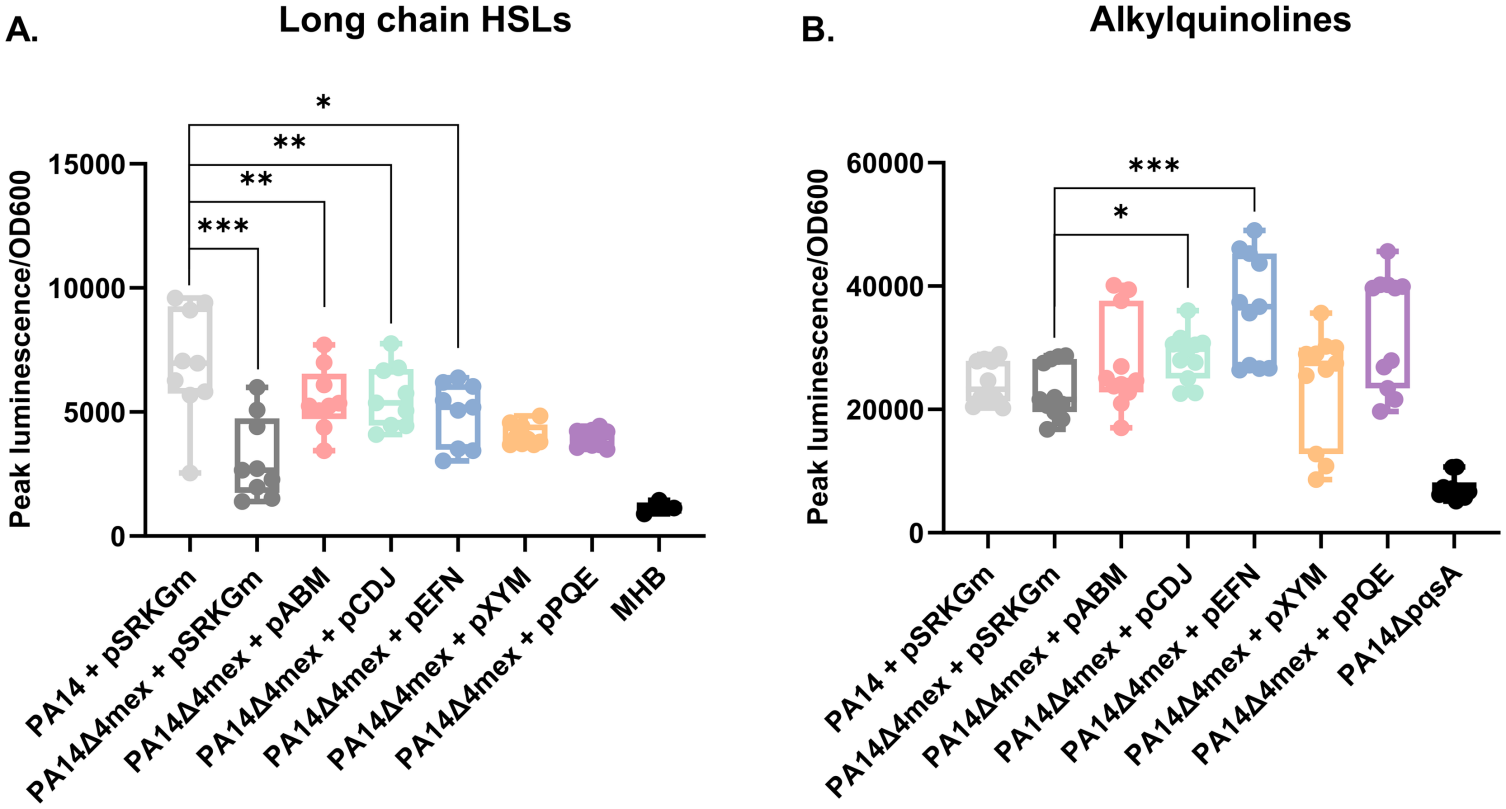
Determination of metabolites in culture supernatants using specific bioassays. **(A)** *E. coli* based luminescence bioassay for detection of long chain HSL. **(B)** Alkylquinolines. PAO1pqsA::lux luminescence based bioassay. The measures were repeated on at least three different occasions. Luminescence was monitored during growth and the values presented are relative light units (RLU) normalized to the OD600 (cell density) at peak luminescence. Statistical analysis: student T-test was performed by GraphPad; *p-value ≤0.05, **p-value ≤0.01, ***p-value ≤0.005.

Using the *E. coli* biosensor strain detecting preferentially long chain HSLs, we confirmed decreased amounts of these quorum sensing auto-inducers in the supernatant of PA14Δ4mex, which were increased again upon individual overexpression of MexAB-OprM, MexCD-OprJ or MexEF-OprN EPs (Figure 6A). This suggests that long chain HSLs are preferentially exported by EPs. Moreover, we observed an increase of AQs in the supernatants of MexCD-OprJ, MexEF-OprN, and MexPQ-OpmE (Figure 6B). In correlation to what was observed in the untargeted metabolomics, there was no significant difference in terms of AQs content between PA14 and PA14Δ4mex (Figure 6B).

Altogether, these data suggest that in the absence of the four major drug EPs, toxic intermediates of well-known *P. aeruginosa* metabolites likely accumulate in the cytosol or inner membrane, and delay growth of the PA14Δ4mex mutant. EPs therefore transport not only antibiotics but also a variety of known natural metabolites, including signaling molecules, components of the quorum sensing systems and the pyochelin byproduct aeruginol. Finally, most natural substrates seem to be EP-specific (Figure 4).

## Discussion

In this study we attempted to gain insights in the natural substrates of Mex EPs from *P. aeruginosa*, by comparing exo-metabolomes of the PA14 wild type strain and a mutant deleted for the four major Mex EPs. In a second part, we further assessed the specific metabolite profile for each of five Mex EPs by overexpressing their operons individually in the same EP deficient strain background.

### *Pseudomonas aeruginosa* Mex efflux pumps are involved in the transport of molecules harboring alkyl side chains

Using both UHPLC–MS and GC–MS, we could show that deleting the four main Mex EPs drastically decreased the fatty acid content in the exo-metabolome. These findings corroborate previous metabolomic studies of endo-and exo-metabolomes from *E. coli* and *Salmonella enterica* Serovar Typhimurium, comparing a wild type strain with a mutant deficient in the constitutively expressed AcrAB-TolC efflux pump (Wang-Kan et al., 2021). The authors found that the AcrAB-TolC system could extrude fatty acids and their oxidized derivatives. AcrB is structurally similar to MexB in *P. aeruginosa*, thus we would expect chemically similar natural substrates between these two export systems. However surprisingly *acrB* mutants in *E. coli* and *Salmonella enterica* Serovar Typhimurium showed non-overlapping substrate profiles (Wang-Kan et al., 2021).

In our unbiased analysis of exo-metabolomes, we identified only predicted fatty acids that are not derived from phospholipids being part of the inner or outer membrane of *P. aeruginosa*. None of these fatty acid constituents were diminished in the EP-deficient mutant PA14Δ4mex compared to PA14, suggesting that Mex EPs in *P. aeruginosa* are not involved in maintaining phospholipid composition.

Indeed, a majority of the fatty acids decreased in the PA14Δ4mex mutant were predicted to be oxidized. Moreover, six of the decreased features in the PA14Δ4mex exo-metabolome were attributed to homoserine lactones, that are oxidized fatty acids. Among them was the quorum sensing autoinducer C12-HSL. This was corroborated by HSL specific biosensor assays, showing increased amounts of long chain HSLs in the supernatant of MexCD-OprJ, MexEF-OprN and MexAB-OprM overexpressors.

Accumulation of potentially toxic (per)oxidized fatty acids due to reactive oxygen species (ROS) production and introduction of unsaturated bonds via the DesA and DesB desaturases of *P. aeruginosa* may affect membrane fluidity and hence growth phenotypes (Pezzoni et al., 2022). Indeed, the RNAseq analysis showed increased expression in the PA14Δ4mex strain of the *desB* desaturase gene, as well as overexpression of the PA14_36010–36,020-36030 operon potentially involved in lipid trafficking between inner and outer membranes (Isom et al., 2020; Isom et al., 2017; Klepp et al., 2022). Since MexAB-OprM is expressed constitutively, like AcrAB in *E. coli*, this function may be important during the bacterial growth cycle. Hence, one of the primary roles of MexAB-OprM could be the removal of oxidized fatty acids, accumulating in the cytoplasmic membrane.

### A majority of natural substrates are EP-specific

Under growth in rich medium (MHB), MexXY and MexEFN showed the highest proportion of EP specific features (65%), while MexAB-OprM, MexCD-OprJ and MexPQ-OpmE, showed roughly the same proportion of EP-specific (47 to 56%) and common features (44 to 53%). Not a single feature of interest (log2(FC) ± 1 and *p*-value ≤0.05) was shared between the five EPs. These results are in contrast with the antibiotic substrate profile of the overexpressed Mex EPs. With the exception of the *β*-lactams carbenicillin and aztreonam, which were specific to MexAB-OprM, all other antibiotic substrates tested here were shared by at least one other EP. This is in support of a specific physiological role for each EP and could explain why these EPs have been maintained on the *P. aeruginosa* core genome during evolution, while their function may have been considered redundant based solely on antibiotic transport.

### Mex efflux pumps transport virulence factors and signaling molecules

*Pseudomonas aeruginosa* produces more than fifty secreted HAQs (Lépine et al., 2004), eleven of which have been characterized: HQNO prevents the growth of Gram-positive bacteria by inhibiting cytochromes of the respiratory chain, while PQS together with its transcriptional regulator PqsR (MvfR) regulates numerous virulence genes but acts also as an iron chelator (Diggle et al., 2007). Both PQS and its precursor HHQ were found in the lungs of cystic fibrosis patients, indicating a potential role in inter-species competition in the chronically infected lung setting (Barr et al., 2017). The EP MexEF-OprN has previously been described to transport HHQ (Lamarche and Déziel, 2011). PQS has also been shown to interact with the EP protein MexG (Hodgkinson et al., 2016). Our metabolomic analysis on culture supernatants predicted at least twelve AQs with side chain lengths containing 7 to 11 carbon atoms. However, the majority were identified also in supernatants from the PA14Δ4mex EP mutant. Previous studies from us and others showed that precursors (kynurenin) of AQ synthesis were effluxed mainly by the MexEF-OprN pump, which translated into reduced expression of QS-regulated virulence factors (Lamarche and Déziel, 2011; Köhler et al., 2001; Olivares et al., 2012). PQS, as well as 3-oxo-C12-HSL, have been shown to be incorporated into outer membrane vesicles (OMVs) (Mashburn-Warren et al., 2008). PQS is also able to induce OMV formation. It remains unclear whether PQS and other AQs might cross the inner membrane by so far unidentified inner membrane transporters to be spontaneously incorporated into OMV without prior export to the external medium or whether other EPs not analyzed here (MexMN, MexJK, MexVW, etc) extrude these molecules into the external medium to be eventually incorporated into preformed OMVs.

Pyochelin, the low affinity siderophore of *P. aeruginosa* was identified in all supernatants including PA14Δ4mex, suggesting that the tested EPs are not involved in pyochelin efflux under our experimental conditions, and export via other EPs or ABC transporters are possible alternatives. Pyoverdin, the high affinity siderophore of *P. aeruginosa* has been shown to be transported via the RND type efflux pump PvdRT-OpmQ (Hannauer et al., 2010) while the siderophore mycobactin was shown to be transported by the MmpL4 RND efflux pump of *Mycobacterium tuberculosis* (Meikle et al., 2023; Wells et al., 2013). Here, we tentatively identified aeruginol, a byproduct of pyochelin biosynthesis but also of spontaneous pyochelin hydrolysis (G. Mislin, personal communication), as a potential substrate of MexPQ-OpmE. Aeruginol as well as its oxidation product aeruginaldehyde (IQS) and aeruginoic acid were shown to chelate iron and promote growth of Pseudomonads (Kaplan et al., 2021).

Interestingly, several predicted EP substrates contain a thiazol group or S-atoms either within heterocycles or as sulfides. These were mainly enriched in supernatants of MexPQ-OpmE, but also in those of the MexCD-OprJ overexpressor. Finally, other known pseudomonal metabolites, including the phenazine antimicrobial compound Questiomycin A (Guo et al., 2022), the QS regulated metabolite acetidomonoamine B (Ernst et al., 2022) and the alkaloid Pyreudione C were predicted as potential EP substrates (Klapper et al., 2018; Klapper et al., 2016). Indole derivatives were predicted as EP substrates in our analysis, which corroborates data from *E. coli*, where indoles were substrates and competitive inhibitors of RND EPs (Zeng et al., 2010; Kawamura-Sato et al., 1999).

In summary, our metabolomic analysis revealed a wide array of potential natural substrates of Mex EPs, covering a broad spectrum of chemical structures and functions. Moreover, the natural EP substrate profiles are mainly non-overlapping and dominated by alkaloid class compounds. This notion contrasts with the more redundant antibiotic substrate profile of these EPs. Exploring the natural substrate profile of *P. aeruginosa* Mex EPs represents an interesting starting point for the development of competitive EP inhibitors. Indeed, using these natural substrates in combination is an interesting strategy to potentiate existing antibiotics in the fight against ESKAPE pathogens (Zeng et al., 2010; Kawamura-Sato et al., 1999).

## Materials and methods

### Bacterial strains and growth conditions

Strains, primers and plasmids used in this study are listed in Table 2. For supernatant extraction, bacteria were grown in Mueller-Hinton (MH) broth at 37°C with shaking (250 rpm). For pSRKGm plasmid selection, media were supplemented with gentamicin 2.5 μg/mL and IPTG 2 mM. MICs were determined according to CLSI guidelines in Mueller-Hinton (MH) broth and when required supplemented with gentamicin 2.5 μg/mL and 2 mM IPTG (Clinical and Laboratory Standards Institute, 2012). Determinations were repeated at least on three different occasions. Growth of EP-overexpressor strains was monitored as optical density at 600 nm (OD600) in 96-well microtiter plates in a BioTek Synergy H1 plate reader under static conditions at 37°C.

**Table 2:** Primers, strains, and plasmids used or constructed in this study.

Primers	Sequence 5′- > 3′	Reference
Deletion of mexXY		
mexXY-hind-F1	ACACAAGCTTGCACATCGCCAGACAGACCT	This study
mexXY-xba-R1	ACACTCTAGAACGTCCTGGCCTTCCTCGTA	This study
mexXY-xba-F2	ACACTCTAGAGAACGCCATCCTCATCATCG	This study
mexXY-xho-R2	ACACCTCGAGGATCCGCTCGGTAGCCTGAC	This study
Deletion of mexCD-oprJ		
mexCDJ-hind-F1	ACACAAGCTTGTCCGGGCGGTACTGGAATA	This study
mexCDJ-bam-R1	ACACGGATCCGAACTCAGCGCCAGGGACTC	This study
mexCDJ-bam-F2	ACACGGATCCTCGACAACCACCTGCGCTAC	This study
mexCDJ-eco-R2	ACACGAATTCGTACCCTCGAACGCCTCACC	This study
Deletion of mexEF-oprN		
mexEFN-hind-F1	ACACAAGCTTCAAGCGCAAGGTGGTCCTG	This study
mexEFN-bam-R1	ACACGGATCCCGGTGAATTCGTCCCACTC	This study
mexEFN-bam-F2	ACACGGATCCGAAGGCACCACCGATTTCCT	This study
mexEFN-eco-R2	ACACGAATTCCCCACCAACAGACCAACAG	This study
Cloning of mexAB-oprM in pSRKGm		
mexABM-F-Xba	ACACTCTAGAGAGGCTTTCGGACGTTTACAA	This study
mexABM-R-Hind	ACACAAGCTTCGACTTCCGCGAGGATAAAA	This study
Cloning of mexCD-oprJ in pSRKGm		
mexC-xba	ACACTCTAGACAATCAACGGTCGGGTGTGT	This study
oprJ-hind	ACACAAGCTTCGTCCTGATCTACGGCATGG	This study
Cloning of mexXY-oprM in pSRKGm		
mexXY-eco-R	ACACGAATTCGTTTCGCTAGGGGCATCAGG	This study
oprM-eco-F	ACACGAATTCGTTCTACGTGGCGGTCAGCA	This study
Cloning of mexEF-oprN in pSRKGm		
mexE-Xba-F	ACACTCTAGAATTAGTTCCCTGCCGGAGCA	This study
oprN-hind-R	ACACAAGCTTCGTCAACCATGGCACCTACC	This study
Cloning of mexPQ-opmE in pSRKGm		
mexPQE-F-bam	ACACGGATCCTTGCCGGACTTCCCTTCCTA	This study
mexPQE-R-hind	ACACAAGCTTAACGCAGAGGCACAGGAGTG	This study

Strains	Genotypes, characteristics	Reference
PA14	Wild type	He et al. (2004)
PA14Δ4mex	Genomic deletion of *mexA/mexB/oprM/mexE/mexF/oprN/mexX/mexY/mexC/mexD/*o*prJ* genes	This study
PA14ΔmexABM	Genomic deletion of *mexA/mexB/oprM* genes. Background for PA14Δ4mex construction	Dr Pletzer
PAO1 pqsA CTX-luxHpqsA	Lux cassette replacing the *pqsA* gene for Aqs quantification	Fletcher et al. (2007)
JM109	Bioluminescence plasmid pSB1075for HSL detection	Winson et al. (1998)
S17-1λpir (TE52)	Strain used for plasmid mobilization	De Lorenzo et al. (1993)
DH10B	F– endA1 deoR+ recA1 galE15 galK16 nupG rpsL Δ(lac)X74 φ80lacZΔM15 araD139 Δ(ara,leu)7697 mcrA Δ(mrr-hsdRMS-mcrBC), SmR, λ–	Laboratory collection

Plasmids	Description	Reference
pEXG2	Suicide vector, Gentamicin resistance cassette	Hmelo et al. (2015)
pSRKGm	Gentamicin resistance cassette, IPTG inducible	Khan et al. (2008)
pSRKGm + mexAB-oprM	mexAB-oprM overexpression, gentamicin resistance cassette, IPTG inducible	This study
pSRKGm + mexEF-oprN	mexEF-oprN overexpression, gentamicin resistance cassette, IPTG inducible	This study
pSRKGm + mexPQ-opmE	mexPQ-opmE overexpression, gentamicin resistance cassette, IPTG inducible	This study
pSRKGm + mexXY-oprM	mexXY-oprM overexpression, gentamicin resistance cassette, IPTG inducible	This study
pSRKGm + mexCD-oprJ	mexCD-oprJ overexpression, gentamicin resistance cassette, IPTG inducible	This study
pSB1075	Bioluminescent, HSL detection	Winson et al. (1998)

### Construction of knockout deletions

The generation of knockout mutants was based on the protocol by Hoang et al. (1998). DNA fragments of 500 to 700 bp in the 5′ and 3′ regions of the genes or operons were PCR-amplified using primer pairs F1/R1 and F2/R2, respectively (Table 2). After amplification, the obtained fragments were gel purified, and approximately 40 ng of each fragment was used in a PCR fusion amplification with primers F1 and R2. The resulting fusion products were gel purified and further cloned into the suicide vector pEXG2 (Rietsch et al., 2005) with the appropriate restriction enzymes. The cloned PCR fragments were verified by Sanger sequencing. The gene replacement vectors were introduced by conjugation into *P. aeruginosa* via biparental mating using *E. coli* strain S17-1λpir as donor. Merodiploid strains were selected on gentamicin containing LB-agar and unmarked mutants yielding the desired genetic deletion were enriched by repeated streaking on low salt LB agar plates supplemented with 10% sucrose. The generated gene knockouts were verified by PCR amplification using the external primers (F1 and R2) and Sanger sequencing.

### Construction of expression plasmids

The coding regions of the genes or operons of interest, including at least 50 nucleotides (nt) upstream of the ATG initiation codon and 20 nt downstream of the STOP codon, were amplified by PCR from *P. aeruginosa* PA14 wild type genomic DNA using the plasmids listed in Table 2. The amplified fragments were digested with corresponding restriction enzymes and cloned into vectors. The Q5 high-fidelity DNA polymerase (NEB) was used for all amplifications. PCR conditions were as follows: denaturation at 98°C for 2 min, followed by 27 cycles of 98°C for 20 s, 57°C for 30 s and 72°C for 2 min, and a final extension at 72°C for 4 min. The plasmids were transferred into *P. aeruginosa* by electroporation, and cells were plated on LB agar supplemented with the appropriate antibiotic selection. Plasmids were also introduced in *E. coli* DH10B by heat shock transformation for conservation in the laboratory conservation. All constructs were verified by Sanger sequencing.

### Alkylquinoline measurement in supernatants

Alkylquinoline measurements in the supernatants were performed using a protocol based on the technique described by Fletcher et al. (2007). In a *pqsA* knockout mutant (PAO1ΔpqsA), a copy of the *pqsA* promoter was linked to the *luxCDABE* genes and inserted into a neutral site on the chromosome. This strain, called PAO1pqsA::luxCDABE was used as a biosensor. For measurement of AQs, the supernatants (SNs) were prepared as previously described and then adjusted to OD600 = 1, centrifuged and filtered through 0.22 μm syringe-driven filters (Millipore). The biosensor was grown overnight in LB and then adjusted to OD600 = 1, and further diluted into fresh LB at concentrations of either 1/50 (test wells) or 1/100 (control wells). 100 μL of 1/50 diluted biosensor was added to each test well, where 100 μL of supernatant were added, for a total of 200 μL per well. The biosensor was then grown for 24 h at 37°C using a BioTek Synergy H1 plate reader. The cell density (OD600) as well as the luminescence were recorded every 30 min. The results are given in relative light units (RLU) per unit of OD600 (luminescence/OD600).

### Pyocyanin quantification

Pyocyanin measurements in the supernatants were performed as follows. Strains of interest were grown for 20 h at 37°C with shaking (250 rpm) in 2 mL LB or MHB supplemented with appropriate antibiotics for plasmid selection and supernatants were prepared as previously described. 500 μL of CHCl_3_ was added to 700 μL of supernatant in Eppendorf tubes, that were then centrifuged for 2 min at 23′000 rpm. The lower phase was removed and 500 μL of 0.2 N HCl was added. Finally, 400 μL of supernatant was mixed with 400 μL of H_2_O in glass cuvettes and absorption was read at 520 nm. The results were expressed as absorption (520 nm)/cell density (OD600) ratios.

### Long chain HSLs quantification

The quantity of long chain HSLs was assessed in supernatants via an *E. coli* reporter strain harboring the bioluminescence plasmid pSB1075 (Priya et al., 2013) carrying a lasI-luxCDBAE reporter fusion Briefly, the *E. coli* strain + pSB1075 was grown overnight in 3 mL LB supplemented with 50 μg/mL ampicillin. The next morning, the overnight culture was diluted 1/10 in fresh LB. The diluted culture was distributed into 96-well plates containing 190 μL per well. Supernatants were added at 10 μL per well, and luminescence as well as growth (OD600) were recorded over 24 h using a microplate reader. The results were given as the peak luminescence/cell density (OD600).

### Transcriptomic analysis

Strains were grown in 96 wells plates, 150 μL per well, at 37°C static over-night in MHB until cell density (OD600) = 1.5–2. Four wells were pooled to form one sample. Two volumes of RNA Protect Bacteria solution (Qiagen) were added. The samples were then pelleted by centrifugation at 13′000 rpm for 5 min at RT. Supernatant was discarded and the pellets were stored at −20°C for later use. RNA was extracted from the pellets using the RNAeasy Qiagen kit according to manufacturer’s instructions. The RNA obtained was immediately frozen at −80°C or underwent DNase treatment using Promega RNAse-free DNAse. RNA quantity was measured at nanodrop after extraction. Approximately 200 ng of total RNA for each sample was ribodepleted using the removal kit for bacteria Ribo-Zero rRNA (Epicentre) according to manufacturer’s instructions. The Illuma kit Stranded Total RNA Ribo-Zero Plus was used to prepare libraries. The Illumina HiSeq4000 instrument was used to sequence (iGE3 Genomics platform of the University of Geneva). Sequencing quality control was done with the FastQC quality control tool. The sequencing quality was determined to be good. The fastq files were mapped to the Ensembl *Pseudomonas aeruginosa* UCBPP-PA14 (GCA_000014625) genome with BWA v.0.7.17 aligner software (Dobin et al., 2013). The average number of mapped reads was 99.65%. The table of counts with the number of reads mapping to each gene feature of Ensembl *Pseudomonas aeruginosa* UCBPP-PA14 (GCA_000014625) reference was prepared with HTSeq v0.9.1 (htseq-count). After normalization, the poorly detected genes were filtered out genes with a count above 10 were kept for the analysis. The differential expression analysis was performed with the statistical analysis R/Bioconductor package edgeR 3.34. with a multiple testing Benjamini and Hochberg correction FDR 5% and a fold change threshold of 2.

### Supernatant extraction for metabolomics analysis

Supernatants were prepared from overnight cultures in 20 mL Mueller-Hinton (MH) broth supplemented with gentamicin 2.5 μg/mL and 2 mM IPTG. Cultures were then collected, centrifuged and filtered using a 0.22 μm filter. Josamycin (Sigma-Aldrich, Switzerland) was spiked (1.25 μg/mL) into each supernatant as an internal control for an expected post-extraction concentration of 75 μg/mL. Filtered supernatants were extracted using four consecutive liquid–liquid extractions with first 20 mL and then 10 mL of dichloromethane followed by 20 mL and 10 mL of ethyl acetate. The combined organic layers were washed with brine and dried over MgSO_4_, filtered, concentrated *in vacuo* and finally under nitrogen flux to yield crude extracts. After determination of their masses, the dried extracts were resuspended in DMSO at a concentration of 10 mg/mL and analyzed by UHPLC MS/MS and/or by GC–MS.

### UHPLC-ESI-HRMS/MS analyses

The UHPLC-ESI-HRMS/MS analysis was carried out on a Waters Acquity UPLC IClass system interfaced to a Q Exactive Focus mass spectrometer (Thermo Scientific, Bremen, Germany), using a heated electrospray ionization (HESI-II) source. Chromatographic separation was performed on a column of Waters BEH C18 50 × 2.1 mm i.d., 1.7 µm, mobile phase consisted of 0.1% formic acid in water (A) and 0.1% formic acid in acetonitrile (B), flow rate was 600 μL/min, injection volume was 1 μL, and linear gradient elution from 5% to 100% B in 7 min, followed by isocratic at 100% B for 1 min, and decreased to 5% B at the final step for 2 min. Positive and negative ionization mode were applied in this study. The diisooctyl phthalate C24H38O4 [M-H]− ion (m/z 389.2697) was used as an internal lock mass. The optimized HESI-II parameters were set as follows: source voltage, 3.5 kV (pos) or 2.5 kV (neg); sheath gas flow rate (N2), 48 units; auxiliary gas flow rate, 11 units; spare gas flow rate, 2.0; capillary temperature, 300°C (pos), S‒Lens RF Level, 55. The mass analyzer was calibrated using a mixture of caffeine, methionine‒arginine‒phenylalanine‒alanine‒acetate (MRFA), sodium dodecyl sulfate, sodium taurocholate, and Ultramark 1,621 in an acetonitrile/methanol/water solution containing 1% formic acid by direct injection. The data‒dependent MS/MS events were performed on the three most intense ions detected in full scan MS (Top3 experiment). The MS/MS isolation window width was 2 Da, and the normalized collision energy (NCE) was set to 35 units. In data-dependent MS/MS experiments, full scans were acquired at a resolution of 35,000 fwhm (at m/z 200) and MS/MS scans at 17,500 fwhm both with a maximum injection time of 50 ms. After being acquired in a MS/MS scan, parent ions were placed in a dynamic exclusion list for 2.0 s.

The chromatographic separation was done on a Waters BEH C18 column (50 × 2.1 mm i.d., 1.7 μm, Waters, Milford, MA) using a gradient as follows (time (min), %B): 0.5,5; 7,99; 8,99; 9.10,5; 9.75, 5. The mobile phases were (A) water with 0.1% formic acid and (B) acetonitrile with 0.1% formic acid. The flow rate was set to 600 μL/min, the injection volume was 1 μL, and the column was kept at 40°C. The PDA detector was used from 210 to 400 nm with a resolution of 1.2 nm. The CAD detector was kept at 40°C, for a data collection rate of 20 Hz. Supernatant extracts from PA14, PA14Δ4mex and EP overexpressing strains were profiled by UHPLC coupled to a Q-Exactive Focus Mass-spectrometer with automated acquisition of MS/MS spectra. The MS data were converted from. RAW (Thermo) standard data format to.mzXML format using the MSConvert software, part of the ProteoWizard package. The converted files were treated using the MZMine software suite v. 4.0.3. The dereplication strategy consisted of a combination of annotation tools: SIRIUS (Dührkop et al., 2019) and MS2Query (de Jonge et al., 2023) for analog prediction. All annotations results were re-ranked using taxonomic information (Tima-R) (Rutz et al., 2019).

### GC–MS analysis

The extracted supernatant was dried and resuspended in chloroform:methanol (3:1) at a concentration of 10 mg/mL. Of this solution, 50 μL were re-dried in a centrifugal evaporator and total fatty acids converted to their methyl esters by reconstituting samples in a mix of chloroform (48%, v/v), methanol (24%, v/v) and 3-(trifluoromethyl)-phenyltrimethyl ammomium hydroxide in methanol (TCI, T0961; 28%, v/v). Samples were analyzed on an 8,890 GC System (Agilent) equipped with a DB5 capillary column (J&W Scientific, 30 m, 250 μm inner diameter, 0.25-μm film thickness), with a 10-m inert duraguard, connected to a 5977B GC/MSD in electron impact (EI) mode linked to a 7693A autosampler (Agilent). The GC–MS settings were as follows: Inlet temperature: 270°C, MS transfer line temperature: 280°C, MS source temperature: 230°C and MS quadrupole temperature: 150°C. The oven temperature gradient during the sample run was as follows: 80°C (2 min); 80°C to 140°C at 30°C/min; 140°C to 250°C at 5°C/min; 250°C to 310°C at 15°C/min; 310°C for 2 min. Samples (1 μL) were injected splitless and analyzed in scan mode (*m/z* 70–700).

Peak detection and deconvolution were performed using MassHunter Unknowns Analysis 12.1 (Agilent), using the default settings but limiting the analysis to features with a signal intensity of >500 counts. Features were tentatively identified using NIST MS Search Program 2.4, considering only those with a match factor of >70. The list of tentatively identified features was further curated manually by removing duplicate identifications, keeping only those with the highest match factor and removing features that were more abundant in fresh medium than in spent medium (MHB). This resulted in a library of 116 putatively secreted metabolites, the associated qualifier ions and retention time of which were exported as a quantitative method. Next, the abundance of each of these putative metabolites was determined across all samples using MassHunter Quantitative Analysis Software 12.0 (Agilent). Compounds were identified based on the presence of three qualifier ions and quantified based on the signal intensity (area under the curve) of the most abundant target ion at the assigned retention time with a left and right retention time delta of 0.06 min.

### Statistical analysis of metabolomic data

The metabolomic data were analyzed as follows for both GC–MS and UHPLC–MS using the MetaboanalystR RStudio package workflow (Pang et al., 2024). Briefly, the features with a constant or single value across all samples were found and deleted. Then the mission values were replaced by the LoDs (1/5 of the minimum positive values of their corresponding variables). The data were then normalized on the internal standard josamycin for the UHPLC–MS analysis or on the median value (each metabolite peak intensity is divided by the median value) for the GC–MS, and then transformed into log10. The fold change analysis was unpaired, and FCs were calculated as the ratio between the two groups means. Features of interest were defined as having a fold change ≥2 and a raw *p*-value ≤0.05. The Principal Component analysis was performed on the Metaboanalyst platform as follows: data were normalized on the internal standard and transformed to log10 and then an autoscaling was performed (mean-centering and division by the standard deviation of each variable). A quality control sample (pooled samples) was used to verify the quality of the PCA analysis. The significance of the analysis was assessed using a permanova permutation test.

## Data Availability

The original contributions presented in the study are publicly available. This data can be found at: https://doi.org/10.5281/zenodo.13361298.
