# Supplementary material for: Multidrug efflux pumps of Pseudomonas aeruginosa show selectivity for their natural substrates
Source: Front Microbiol. 2025 Jan 9;15:1512472. doi: 10.3389/fmicb.2024.1512472 (PMC11754269; doi:10.3389/fmicb.2024.1512472)
Supplement: Supplementary file 1 [file Data_Sheet_1.pdf]

Supplementary Tables, Mazza et al.

Table S1. Differentially expressed genes PA14Δ4mex vs PA14 - RNA sequencing

EnsemblID	Symbol	Description	logFC	logCPM	PValue	FDR
**PA14_31170**	NA	hypothetical protein	2.48699618	1.426172458	1.52E-06	0.000242748
**PA14_36010**	NA	hypothetical protein	2.443068203	5.667605329	6.63E-11	7.20E-08
**PA14_31160**	NA	hypothetical protein	2.322325949	3.939191275	1.75E-07	5.28E-05
**PA14_44890**	hcpA	secreted protein Hcp	1.952255716	1.846422841	3.39E-05	0.002438034
**PA14_43080**	NA	conserved hypothetical protein	1.91545476	1.101458693	0.000185118	0.008643337
**PA14_03240**	hcpC	Secreted protein Hcp	1.912779773	2.703346597	7.29E-05	0.004565533
**PA14_36020**	NA	paraquat-inducible protein B	1.841660938	6.174943379	4.86E-10	3.10E-07
**PA14_28130**	NA	putative transcriptional regulator	1.736798681	4.775371441	2.38E-05	0.001926507
**PA14_41290**	NA	hypothetical protein	1.708467481	0.623995192	0.002658169	0.043967881
**PA14_37470**	NA	putative flavin-dependent oxidoreductase	1.648903785	1.397707847	0.000500718	0.017428021
**PA14_64610**	NA	putative fatty acid desaturase	1.638056701	6.397099596	0.000512383	0.017620442
**PA14_28120**	NA	hypothetical protein	1.594580331	4.622375072	3.43E-06	0.000458538
**PA14_43090**	NA	hypothetical protein	1.556902312	3.211905714	0.000107624	0.0060596
**PA14_20200**	nosZ	nitrous-oxide reductase precursor	1.443445983	6.926427512	0.00183863	0.037236299
**PA14_37460**	NA	putative permease	1.428743039	1.667098716	0.000846334	0.023943334
**PA14_13770**	narK2	nitrite extrusion protein	1.41497872	2.465721054	3.78E-05	0.002648277
**PA14_40040**	NA	putative penicillin acylase	1.372801307	5.372005919	2.21E-06	0.000325329
**PA14_40030**	NA	putative enzyme	1.330069143	5.346481944	3.13E-05	0.002326569
**PA14_10370**	choS	putative cholesterol oxidase	1.291291167	7.467346891	7.04E-06	0.000827127
**PA14_20190**	nosD	copper ABC transporter%2C periplasmic copper-binding protein	1.291247167	4.854424065	0.000243038	0.010510753
**PA14_41280**	NA	putative beta-lactamase	1.262116552	3.814665101	1.01E-05	0.00099314

**PA14_40020**	NA	hypothetical protein	1.22840464	5.092721661	3.14E-05	0.002326569
**PA14_10380**	NA	hypothetical protein	1.193021343	9.010128489	0.000387635	0.014645957
**PA14_40050**	NA	hypothetical protein	1.18833535	3.822997836	2.69E-05	0.002119277
**PA14_69540**	NA	hypothetical protein	1.188204956	3.381827454	2.33E-05	0.001926507
**PA14_06830**	norB	nitric-oxide reductase subunit B	1.18657583	7.775246005	0.000147663	0.007504693
**PA14_13750**	narK1	putative nitrite extrusion protein	1.160730377	3.499364896	0.000146186	0.007495966
**PA14_26090**	NA	putative hydrolase	1.158593512	6.998640209	0.00164952	0.0357412
**PA14_58330**	NA	conserved hypothetical protein	1.155628467	10.27793837	0.001058026	0.027872689
**PA14_45100**	muiA	putative mucoidy inhibitor A	1.152476521	8.280029438	0.002613856	0.043967881
**PA14_53400**	NA	probable oxidoreductase	1.141109204	5.599505176	8.13E-05	0.004967655
**PA14_22350**	yjcG	putative sodium/proline:solute symporter	1.123466679	6.265249596	3.68E-06	0.00046967
**PA14_36030**	NA	paraquat-inducible protein A	1.092317166	4.825096788	1.35E-06	0.000227193
**PA14_37430**	NA	putative sigma-70 factor%2C ECF subfamily	1.087226497	2.525676344	0.002420645	0.04366245
**PA14_53410**	NA	probable transcriptional regulator	1.061347843	4.35542256	0.001142825	0.029697945
**PA14_59440**	NA	conserved hypothetical protein	1.058365895	4.314388014	0.000690096	0.021064671
**PA14_32250**	NA	hypothetical protein	1.048272203	2.969156889	0.002685723	0.043967881
**PA14_51470**	cupC1	fimbrial subunit CupC1	1.014418057	4.31394401	3.16E-05	0.002326569
**PA14_06600**	NA	putative acyl-CoA dehydrogenase	-1.055072034	11.25766813	0.000217066	0.009663635
**PA14_08000**	NA	conserved hypothetical protein	-1.056208041	7.046562982	0.000157122	0.007814077
**PA14_13330**	NA	putative phosphatase or phosphodiesterase	-1.056394552	2.850040853	0.00037444	0.014432264
**PA14_08060**	NA	putative tail fiber assembly protein	-1.06130377	7.116017413	2.83E-05	0.002166434
**PA14_06640**	NA	putative acyl-CoA dehydrogenase	-1.062434449	8.643922579	0.000594001	0.019164862
**PA14_24170**	fadH1	2%2C4-dienoyl-CoA reductase FadH1	-1.071254518	5.560675314	7.19E-06	0.000827127
**PA14_01520**	NA	putative transcriptional regulator	-1.077789087	3.322043787	0.001127181	0.029424552
**PA14_68110**	NA	putative multiple antibiotic resistance protein MarR	-1.082224308	4.265983264	0.000284273	0.011736234
**PA14_33920**	NA	putative transcriptional regulator	-1.091272402	1.744063514	0.002584873	0.043790349
**PA14_32400**	mexE	RND multidrug efflux membrane fusion protein MexE precursor	-1.093285306	3.852776857	0.000493123	0.017268339
**PA14_47510**	NA	putative hydrolase	-1.094931206	4.141098943	0.0002861	0.011736234

**PA14_08050**	NA	putative tail fiber protein	-1.108322647	9.097565438	3.67E-06	0.00046967
**PA14_47420**	NA	conserved hypothetical protein	-1.11832147	3.321656577	0.000570925	0.018836609
**PA14_53530**	NA	hypothetical protein	-1.142031825	6.961372521	7.03E-06	0.000827127
**PA14_55240**	NA	hypothetical protein	-1.153867366	3.275243916	9.45E-05	0.005509676
**PA14_49080**	lcaD	probable acyl-CoA dehydrogenase	-1.158189566	4.905833062	2.64E-05	0.002105336
**PA14_07990**	hol	putative holin	-1.171556596	4.811592056	0.000242562	0.010510753
**PA14_08280**	NA	putative phage tail assembly protein	-1.193424174	6.43316097	8.57E-06	0.00092854
**PA14_18870**	NA	conserved hypothetical protein	-1.200088156	6.858759406	0.00156089	0.034880124
**PA14_08020**	gpW	putative baseplate assembly protein W	-1.202701217	6.274398257	1.99E-06	0.000301126
**PA14_08250**	NA	putative phage minor tail protein	-1.250031319	5.865557397	7.53E-06	0.000844143
**PA14_17640**	potA	polyamine transport protein PotA	-1.272436886	2.233571978	0.000695103	0.021064671
**PA14_08140**	XR2	putative phage protein X	-1.281393209	5.560449571	9.60E-06	0.00099314
**PA14_08010**	gpV	putative baseplate assembly protein V	-1.326818649	7.019703644	7.03E-07	0.000134658
**PA14_52510**	NA	hypothetical protein	-1.33005831	5.021737222	1.49E-06	0.000242748
**PA14_60850**	mexC	multidrug efflux RND membrane fusion protein	-1.33499538	5.385789432	4.02E-05	0.002782035
**PA14_08100**	NA	conserved hypothetical protein	-1.336557423	6.913978971	2.18E-07	5.97E-05
**PA14_08150**	gpD	putative phage late control gene D protein	-1.356110326	7.498848522	8.01E-07	0.000143703
**PA14_08300**	JF1	putative phage-related protein%2C tail component	-1.36305928	9.141028817	1.70E-07	5.28E-05
**PA14_53820**	NA	hypothetical protein	-1.363777556	7.550069714	1.56E-07	5.26E-05
**PA14_08200**	NA	hypothetical protein	-1.364943443	3.956291806	2.14E-05	0.001830961
**PA14_21530**	NA	putative ankyrin domain protein	-1.367379089	8.64415515	0.001042296	0.027712538
**PA14_08040**	gpI	putative phage tail protein	-1.412006416	7.265832194	9.89E-06	0.00099314
**PA14_08090**	gpFII	putative phage tail tube protein	-1.437979674	8.015286889	3.41E-07	8.17E-05
**PA14_08130**	gpU	putative tail formation protein	-1.438675755	7.150443178	2.56E-07	6.38E-05
**PA14_08110**	NA	hypothetical protein	-1.442984238	5.870486346	1.63E-06	0.000252735
**PA14_08120**	NA	putative tail length determinator protein	-1.444702362	9.601058737	1.20E-06	0.000208329
**PA14_08030**	NA	putative phage baseplate assembly protein	-1.448176396	7.785607051	4.47E-07	9.88E-05
**PA14_08240**	NA	putative tail length determination protein	-1.464935214	8.60336974	2.44E-07	6.36E-05

**PA14_34320**	NA	putative monooxygenase%2C DszC family	-1.507326553	1.424725787	0.002876747	0.045358539
**PA14_08210**	NA	putative major tail protein V	-1.514678523	8.412214768	7.42E-07	0.000137552
**PA14_08070**	gpFI	putative phage tail sheath protein	-1.541634227	9.609154717	1.98E-07	5.69E-05
**PA14_08220**	NA	hypothetical protein	-1.573335051	7.687314275	3.05E-06	0.000416513
**PA14_07980**	NA	conserved hypothetical protein	-1.578240399	8.019120725	5.68E-07	0.000116448
**PA14_08160**	lys	putative lytic enzyme	-1.59110089	6.137289358	2.70E-08	1.19E-05
**PA14_08270**	NA	conserved hypothetical protein	-1.617279571	6.836069117	2.57E-08	1.19E-05
**PA14_08230**	NA	hypothetical protein	-1.61779142	7.746486709	2.38E-08	1.19E-05
**PA14_08260**	NA	putative minor tail protein L	-1.621134976	6.476113016	4.46E-08	1.83E-05
**PA14_08190**	NA	hypothetical protein	-1.688006432	4.196947475	1.18E-05	0.001097234
**PA14_52480**	NA	hypothetical protein	-1.688176562	5.795627435	2.26E-08	1.19E-05
**PA14_39590**	metE	5-methyltetrahydropteroyltriglutamate-homocysteine S-methyltransferase	-1.698720244	8.517758755	1.85E-05	0.001606452
**PA14_52490**	NA	hypothetical protein	-1.733181766	5.931212399	1.45E-07	5.22E-05
**PA14_07970**	NA	putative zinc finger protein	-1.7485437	6.428921593	9.62E-08	3.68E-05
**PA14_52500**	NA	hypothetical protein	-1.782163822	4.382498532	3.82E-07	8.77E-05
**PA14_42620**	pscR	translocation protein in type III secretion	-1.786671641	0.771382077	0.001490669	0.034211682
**PA14_02490**	tonB2	putative TonB protein	-1.80214052	0.724260785	0.001787268	0.036789531
**PA14_61330**	mgtC	putative magnesium transporter%2C MgtC family	-1.820701744	2.120116772	0.000111121	0.006136226
**PA14_08180**	NA	hypothetical protein	-1.840936289	3.501414983	2.35E-05	0.001926507
**PA14_63740**	NA	conserved hypothetical protein	-1.845357529	0.984075407	0.000123074	0.006569983
**PA14_53300**	NA	probable alkyl hydroperoxide reductase	-1.875429518	10.38320456	0.000174562	0.008354264
**PA14_31150**	NA	hypothetical protein	-2.326930107	6.041495905	2.64E-10	1.90E-07
**PA14_31130**	NA	conserved hypothetical protein	-2.708132823	2.032515009	3.40E-05	0.002438034
**PA14_63750**	NA	putative Na+/phosphate symporter	-2.722073214	3.678111792	4.76E-07	0.000101269
**PA14_63770**	NA	hypothetical protein	-2.815995494	0.607956267	0.002304315	0.042572659
**PA14_38410**	amrB	RND multidrug efflux transporter	-3.007917272	4.042704957	2.53E-10	1.90E-07
**PA14_63800**	mgtA	Mg(2+) transport ATPase%2C P-type 2	-3.191042646	6.305443386	1.02E-05	0.00099314
**PA14_63780**	NA	conserved hypothetical protein	-3.348698	3.005277313	3.04E-06	0.000416513

**PA14_05530**	mexA	RND multidrug efflux membrane fusion protein MexA precursor	-6.757481639	8.488537549	1.24E-15	3.56E-12
**PA14_05550**	oprM	major intrinsic multiple antibiotic resistance efflux outer membrane protein OprM precursor	-8.931585608	8.56190354	1.98E-16	1.14E-12
**PA14_32390**	mexF	RND multidrug efflux transporter MexF	-10.41672987	4.013085557	7.53E-11	7.20E-08
**PA14_60830**	mexD	multidrug efflux RND transporter MexD	-12.05911852	6.05089136	1.04E-14	1.49E-11
**PA14_05540**	mexB	RND multidrug efflux transporter MexB	-16.14491175	9.715274183	1.95E-15	3.72E-12

Table S2. Features with differential abundance in PA14Δ4mex vs PA14 WT and EPs overexpressors vs PA14Δ4mex – UHPLC-MS

**mexAB-oprM increased features (vs PA14Δ4mex)** log2(FC) > 1 n= 62						
**ID**	**log2(FC)**	**row m/z**	**RT**	**NPC superclass**	**NPC pathway**	**Candidate inchikey**
**3025**	8.6376	292.1909	4.891997	unknown	Terpenoids	QPGUMJSECSFGCC
**2691**	7.2988	278.1758	4.479116	Linear polyketides	Alkaloids	BGKRPVOZROQPBE
**1301**	5.6241	854.5027	2.504773	unknown	unknown	VCMQMWKGIHWSPX
**2675**	5.4037	308.1655	4.4969	Tryptophan alkaloids	Alkaloids	PDWXCPOZTNNZCY
**2422**	5.2948	264.1591	4.164217	Phenolic acids (C6-C1)	Shikimates and Phenylpropanoids	ASXNZRSELVPGQC
**1512**	5.1954	269.0918	2.904994	Anthranilic acid alkaloids	Alkaloids	OTRRZLYYKZUVRM
**1625**	4.7608	293.1862	3.038829	Peptide alkaloids	Alkaloids	WUPLEXBYYUOWQF
**1621**	4.6949	315.1679	3.038101	unknown	unknown	WSFQKSIBZODGPB
**1205**	4.686	233.1318	2.34736	Fatty amides	Fatty acids	HNZKRSKSIABLNS
**2420**	4.6763	120.0443	4.162898	unknown	unknown	RWZYAGGXGHYGMB
**1400**	4.6012	854.5019	2.678333	unknown	unknown	VCMQMWKGIHWSPX
**3048**	4.4533	683.4728	4.900607	unknown	unknown	QWJFFNXSWQZKKL
**3044**	4.4154	348.2741	4.904153	Fatty Acids and Conjugates	Fatty acids	UDMBCSSLTHHNCD
**1844**	4.3301	292.1542	3.368793	Linear polyketides	Fatty acids	LJFWHSLCSLLNMJ
**774**	4.0057	357.1448	1.628669	Anthranilic acid alkaloids	Alkaloids	WVPWAWQSZCUQGO
**2547**	3.6137	326.1742	4.337158	Anthranilic acid alkaloids	Alkaloids	CXPGERDAOAFZJT
**645**	3.5255	170.1174	1.389183	Small peptides	Amino acids and Peptides	KLYKJOPFNDDFNE
**1395**	3.4199	204.1385	2.656437	Tryptophan alkaloids	Alkaloids	DNTGGZPQPQTDQF
**2452**	3.391	915.4866	4.222682	unknown	unknown	GSZLUEQWCUVBFT

**1281**	3.3512	299.1026	2.486549	Anthranilic acid alkaloids	Alkaloids	KQPFLOCEYZIIRD
**2693**	3.3494	328.1908	4.515947	Tryptophan alkaloids	Alkaloids	BMNMZWYECBBAJT
**3105**	3.0641	356.2219	4.9654	Macrolides	Alkaloids	JNVITAJZSSMIIU
**2461**	3.0122	260.2004	4.230392	Tryptophan alkaloids	Alkaloids	AEGYQCQXUUGBRT
**1385**	2.9817	323.0515	2.631801	Serine alkaloids	Alkaloids	HZCUBLLQCILJTN
**4197**	2.9506	516.2973	6.098938	Small peptides	Alkaloids	WBWWGRHZICKQGZ
**2916**	2.9286	553.2566	4.766755	Tryptophan alkaloids	Alkaloids	XPESNHSGXNAATK
**1697**	2.69	128.0528	3.14233	Lysine alkaloids	Alkaloids	CMOIEFFAOUQJPS
**2442**	2.6349	478.2157	4.226776	Small peptides	Amino acids and Peptides	DXCYJJKWPZZHNZ
**2340**	2.6113	324.1566	4.046235	unknown	unknown	SHANZLCFUGOMJA
**1160**	2.609	353.1973	2.275326	Tryptophan alkaloids	Alkaloids	QLPBAXRSKZBNQY
**2683**	2.5939	476.1267	4.50358	Anthranilic acid alkaloids	Alkaloids	QGPZBTSBJQUOSK
**2722**	2.576	302.2114	4.566289	Tryptophan alkaloids	Alkaloids	WVBVVWIGEPISMW
**2678**	2.5326	475.1195	4.49622	Peptide alkaloids	Alkaloids	CRISVSOALHAQCE
**2532**	2.4915	375.2102	4.318599	Ornithine alkaloids	Alkaloids	YTSDPGXQZGDXTJ
**2682**	2.4874	270.1001	4.500824	Pseudoalkaloids	Alkaloids	ZNAULGVROLOSBS
**2494**	2.4816	325.1987	4.26857	Fatty Acids and Conjugates	Polyketides	YOSVFFVBSPQTTP
**1012**	2.4251	118.065	1.996237	unknown	unknown	SUSQOBVLVYHIEX
**4016**	2.4236	578.4262	5.876802	Glycerolipids	Fatty acids	PAFOXYLFALKPOE
**1985**	2.3587	178.0495	3.559725	unknown	unknown	RVWZUOPFHTYIEO
**5866**	2.314	826.4658	8.177539	Anthranilic acid alkaloids	unknown	NLUFZUPOQMATMY
**683**	2.3037	309.1557	1.449104	Small peptides	Alkaloids	BFSYFTQDGRDJNV
**3066**	2.2641	313.2373	4.90437	Octadecanoids	Fatty acids	FIFLDFYODFCEFA
**3035**	2.2569	640.3906	4.895848	unknown	Polyketides	INNHOUBLAKKYHI
**2338**	2.2199	178.0499	4.044773	unknown	Amino acids and Peptides	RVWZUOPFHTYIEO
**1710**	2.1744	178.032	3.138597	Phenolic acids (C6-C1)	Shikimates and Phenylpropanoids	LOGPRZMQSREDOU
**3155**	2.1579	116.0528	5.055428	Oligopeptides	Fatty acids	TUFJIDJGIQOYFY
**1522**	2.1289	178.0318	2.892573	Phenolic acids (C6-C1)	Shikimates and Phenylpropanoids	LOGPRZMQSREDOU
**815**	2.0614	285.0873	1.655001	Anthranilic acid alkaloids	Alkaloids	AUBMZQABQDPFKS

**2710**	2.0348	312.1968	4.543877	Tryptophan alkaloids	Alkaloids	AWAMJTQSWCOGDM
**1305**	2.021	290.139	2.517695	Anthranilic acid alkaloids	Alkaloids	BFSBNVPBVGFFCF
**1005**	1.9988	136.0757	1.994887	Nicotinic acid alkaloids	Alkaloids	VYFYYTLLBUKUHU
**1703**	1.991	702.0377	3.137458	unknown	unknown	unknown
**2520**	1.8892	391.2243	4.297929	Small peptides	Amino acids and Peptides	FDDUXCZCGGJQHT
**2497**	1.8148	115.0753	4.271053	unknown	unknown	CQJHAULYLJXJNL
**2460**	1.7934	258.1854	4.224319	Tryptophan alkaloids	Alkaloids	JMNXQKCZLWIUSQ
**3145**	1.6918	522.3638	4.963509		Fatty acids	PZRFVAHZNWPPAC
**3873**	1.6543	578.4264	5.844245	Fatty amides	Fatty acids	PAFOXYLFALKPOE
**2785**	1.6524	949.4177	4.638897	unknown	unknown	MOAREQZIZKPJBU
**2716**	1.6107	314.2114	4.538503	Anthranilic acid alkaloids	Alkaloids	LWYHXEXQBAUDEY
**3033**	1.57	1003.62	4.892698	Small peptides	unknown	unknown
**2715**	1.5298	315.2148	4.536043	Small peptides	Amino acids and Peptides	PRAWUGHUUSVULS
**3513**	1.3383	536.3796	5.490673	Fatty Acids and Conjugates	Fatty acids	JMNXFZCXVGEDAC
**mexCD-oprJ increased features (vs PA14Δ4mex)** log2(FC) > 1 n= 49						
**ID**	**log2(FC)**	**row m/z**	**RT**	**NPC superclass**	**NPC pathway**	**Candidate inchikey**
**988**	9.0467	259.0537	1.951714	Nicotinic acid alkaloids	Alkaloids	WFHIVHVSLQQGHS
**2855**	6.1824	395.1741	4.713027	Tryptophan alkaloids	Alkaloids	LHYVYCQNULXZNE
**2457**	5.9914	484.1707	4.244044	Pseudoalkaloids	Alkaloids	FLDHEBAYUPEYMZ
**1210**	5.3707	345.0341	2.370744	Serine alkaloids	Alkaloids	SBHXYTNGIZCORC
**941**	5.0659	362.0623	1.873886	Small peptides	Alkaloids	VMKDFEOKUWRINT
**2971**	5.0246	649.1105	4.825598	Small peptides	Alkaloids	PMYMMQNSAGKMHX
**1081**	4.6948	394.0347	2.146615	Tryptophan alkaloids	Alkaloids	QCMCIUCOCAPEJI
**3343**	4.565	357.2627	5.269464	Triterpenoids	Fatty acids	VPRZXCNKOANCBI
**1086**	4.5616	273.0692	2.136337	Nicotinic acid alkaloids	Alkaloids	PHFSBARLASYIFM
**1808**	4.1883	243.0591	3.299184	Nicotinic acid alkaloids	Alkaloids	HNGJLMHFCIQECQ
**3180**	4.1795	514.2165	5.118917	Pseudoalkaloids	Alkaloids	AKEOLENLJFVVTD
**3371**	4.1618	540.2335	5.321324	Linear polyketides	Alkaloids	UONHTCLFKUIIEE
**2116**	4.0995	257.0749	3.733677	Nicotinic acid alkaloids	Alkaloids	LOYXTWZXLWHMBX
**2698**	3.7566	486.1851	4.518549	Tryptophan alkaloids	Alkaloids	BQNGWUKHPUZJBE

**3135**	3.7038	450.1148	5.011806	Tyrosine alkaloids	Alkaloids	QGGNFPVBCAGWRP
**1400**	3.6902	854.5019	2.678333	unknown	unknown	VCMQMWKGIHWSPX
**3134**	3.4844	451.121	5.011435	Fatty esters	Alkaloids	KSEXWHUUWQCZSN
**3729**	3.3666	490.373	5.721461	Diterpenoids	unknown	AHUPTCAANZMABA
**2691**	3.3589	278.1758	4.479116	Linear polyketides	Alkaloids	BGKRPVOZROQPBE
**891**	3.3197	342.1442	1.788989	Tryptophan alkaloids	Alkaloids	GXKCUFUYTFWGNK
**2480**	3.2456	423.0922	4.248278	Anthranilic acid alkaloids	Alkaloids	ITPHOIFCAFNCLL
**943**	3.2404	293.0052	1.88353	Fatty esters	Terpenoids	XMOUUBJGOKHMTB
**3375**	3.2275	541.3332	5.344727	Anthranilic acid alkaloids	Fatty acids	YIOSSWUAQNGRSJ
**1301**	3.0776	854.5027	2.504773	unknown	unknown	VCMQMWKGIHWSPX
**1360**	3.0553	309.148	2.599236	Nicotinic acid alkaloids	Alkaloids	WSARFUKLLMQDDB
**2897**	2.9706	451.1219	4.753029	Anthranilic acid alkaloids	Alkaloids	ITOSXXHGQJTRPF
**2943**	2.8823	512.2007	4.816349	Ornithine alkaloids	Alkaloids	ZBQJUUDIPOFIAD
**1624**	2.7354	336.1577	3.013309	unknown	unknown	UQHKFADEQIVWID
**1625**	2.6832	293.1862	3.038829	Peptide alkaloids	Alkaloids	WUPLEXBYYUOWQF
**4517**	2.5863	605.3605	6.418253	Sphingolipids	Terpenoids	SOEBXBQCTCBBNC
**610**	2.5104	204.1248	1.332437	Small peptides	Alkaloids	HYVABZIGRDEKCD
**3923**	2.4635	501.2157	5.879335	Anthranilic acid alkaloids	Alkaloids	ZHAMDNLRQOFZPU
**1844**	2.4381	292.1542	3.368793	Linear polyketides	Fatty acids	LJFWHSLCSLLNMJ
**2068**	2.3562	336.2286	3.679403	Tryptophan alkaloids	Alkaloids	XPZFMHCHEWYIGE
**1522**	2.3488	178.0318	2.892573	Phenolic acids (C6-C1)	Shikimates and Phenylpropanoids	LOGPRZMQSREDOU
**811**	2.2674	283.1045	1.579996	Tryptophan alkaloids	Alkaloids	YWBRXRXMXOEXLY
**1980**	2.2097	573.2934	3.556648	unknown	unknown	DVKZXACHTUATJV
**4481**	2.1854	435.3085	6.401498	Sesquiterpenoids	Fatty acids	MNGASGFOKSKGQQ
**5825**	2.1517	746.4902	8.071898	Small peptides	Amino acids and Peptides	OJAJNPUYPONDBD
**3209**	2.1314	318.2067	5.151517	Tryptophan alkaloids	Alkaloids	BJEPYKJPYRNKOW
**1213**	2.1267	323.0517	2.368219	Serine alkaloids	Alkaloids	HZCUBLLQCILJTN
**2520**	1.9485	391.2243	4.297929	Small peptides	Amino acids and Peptides	FDDUXCZCGGJQHT
**1012**	1.9188	118.065	1.996237	unknown	unknown	SUSQOBVLVYHIEX

**2196**	1.9058	276.1869	3.863899	Peptide alkaloids	Alkaloids	SJIMDGIDDDGXLI
**506**	1.9007	193.0972	1.14341	Ornithine alkaloids	Alkaloids	WNKLVRDIRPADMZ
**2075**	1.5748	291.1703	3.681136	Small peptides	Alkaloids	WXEMUKVRWINVJS
**2073**	1.5484	147.0553	3.680765	Anthranilic acid alkaloids	Alkaloids	YJVOWRAWFXRESP
**2785**	1.4403	949.4177	4.638897	unknown	unknown	MOAREQZIZKPJBU
**2420**	1.1309	120.0443	4.162898	unknown	unknown	RWZYAGGXGHYGMB
**mexEF-oprN increased features (vs PA14Δ4mex)** log2(FC) > 1 n= 36						
**ID**	**log2(FC)**	**row m/z**	**RT**	**NPC superclass**	**NPC pathway**	**Candidate inchikey**
**1545**	7.7607	276.1385	2.905134	Tryptophan alkaloids	Alkaloids	WUGMRIBZSVSJNP
**1001**	4.84	371.161	1.976042	Flavonoids	Shikimates and Phenylpropanoids	WGASWKHIQMYEHL
**3133**	4.7271	329.2308	5.007749	Tryptophan alkaloids	Fatty acids	MKYUCBXUUSZMQB
**3212**	4.6931	361.214	5.130691	Fatty Acids and Conjugates	Alkaloids	DOUASPUQJMFLIU
**994**	4.6484	221.1284	1.971988	Lysine alkaloids	Alkaloids	UISQDUMVIZJYEI
**506**	4.0298	193.0972	1.14341	Ornithine alkaloids	Alkaloids	WNKLVRDIRPADMZ
**2410**	4.0123	328.1898	4.128937	Fatty amides	Fatty acids	UMVKTYCOYYLLPX
**509**	3.9703	194.1007	1.140785	Pseudoalkaloids	Alkaloids	RITVBFZKAMDJHU
**1205**	3.6595	233.1318	2.34736	Fatty amides	Fatty acids	HNZKRSKSIABLNS
**3364**	3.6384	356.259	5.296839	Anthranilic acid alkaloids	unknown	OZKSAMXSQQCGQT
**1204**	3.493	385.1771	2.347075	Meroterpenoids	Terpenoids	OLVSVPOPXGQLBV
**749**	3.4849	207.1128	1.558318	Small peptides	Alkaloids	YEMXDMCTTAKDIF
**774**	3.2603	357.1448	1.628669	Anthranilic acid alkaloids	Alkaloids	WVPWAWQSZCUQGO
**20**	3.1314	322.9197	0.312565	Fatty acyls	Fatty acids	unknown
**746**	3.0526	283.1045	1.548832	Small peptides	Alkaloids	YWBRXRXMXOEXLY
**2037**	2.9375	968.5688	3.621739	unknown	unknown	unknown
**811**	2.8374	283.1045	1.579996	Tryptophan alkaloids	Alkaloids	YWBRXRXMXOEXLY
**1305**	2.8155	290.139	2.517695	Anthranilic acid alkaloids	Alkaloids	BFSBNVPBVGFFCF
**3935**	2.7719	387.2467	5.904247	Meroterpenoids	Fatty acids	XREILSQAXUAAHP
**3343**	2.7711	357.2627	5.269464	Triterpenoids	Fatty acids	VPRZXCNKOANCBI
**3729**	2.7275	490.373	5.721461	Diterpenoids	unknown	AHUPTCAANZMABA

**1114**	2.6798	231.1157	2.187523	Small peptides	Amino acids and Peptides	PLPBLFIRKVGHBJ
**17**	2.634	244.9057	0.309326	unknown	unknown	unknown
**1615**	2.4791	256.1695	3.03548	Tryptophan alkaloids	Alkaloids	IUTFNOOGPQDULV
**2722**	2.4424	302.2114	4.566289	Tryptophan alkaloids	Alkaloids	WVBVVWIGEPISMW
**178**	2.4157	177.1023	0.607981	Tryptophan alkaloids	Alkaloids	QZAYGJVTTNCVMB
**3131**	2.3664	385.2486	5.0049	Triterpenoids	Alkaloids	LRRYXTKTIYROOY
**2324**	2.2279	328.1909	4.021801	Fatty amides	Fatty acids	HWFNEQJAINFFPD
**4106**	2.119	385.2311	6.041934	Steroids	Terpenoids	PSYJGKOWZZOMRS
**770**	2.112	257.0917	1.611991	Anthranilic acid alkaloids	Alkaloids	CUYPWOPOBAHCCE
**405**	2.0875	193.1335	0.97786	Tryptophan alkaloids	Alkaloids	AVIMCWMLFNLXJX
**1120**	1.9607	383.1614	2.192065	Sesquiterpenoids	Terpenoids	VMSQKUCYEMOKMM
**2559**	1.9167	326.211	4.353331	Tryptophan alkaloids	Alkaloids	OAXPQNCOMDEHMJ
**2693**	1.6994	328.1908	4.515947	Tryptophan alkaloids	Alkaloids	BMNMZWYECBBAJT
**2500**	1.668	303.2156	4.28352	Fatty Acids and Conjugates	Fatty acids	GTJOQTFPQLBALB
**1346**	1.2923	216.1383	2.530587	Lysine alkaloids	Alkaloids	FJUBKTNNXRFHFD
**mexXY-oprM increased features (vs PA14Δ4mex)** log2(FC) > 1 n= 83						
**ID**	**log2(FC)**	**row m/z**	**RT**	**NPC#superclass**	**NPC#pathway**	**candidate_structure_inchikey_no_stereo**
**2589**	6.515	393.1631	4.378405	Anthranilic acid alkaloids	Alkaloids	IAIWBAHUWORDTI
**1695**	6.4148	269.0916	3.116656	Small peptides	Alkaloids	RKFYTTOYCOWKNS
**507**	4.9052	511.0618	1.120189	Nucleosides	Alkaloids	CRZOICRNCTVVDV
**1766**	4.62	255.0768	3.184649	Peptide alkaloids	Alkaloids	CCTIOCVIZPCTGO
**1512**	4.495	269.0918	2.904994	Anthranilic acid alkaloids	Alkaloids	OTRRZLYYKZUVRM
**2410**	4.309	328.1898	4.128937	Fatty amides	Fatty acids	UMVKTYCOYYLLPX
**2287**	4.2948	589.3644	3.982906	Steroids	Terpenoids	KVEGLZCDCVSBQX
**3025**	4.2602	292.1909	4.891997	unknown	Terpenoids	QPGUMJSECSFGCC
**1613**	3.9783	255.0768	3.016421	Anthranilic acid alkaloids	Alkaloids	CCTIOCVIZPCTGO
**2037**	3.9034	968.5688	3.621739	unknown	unknown	unknown
**2641**	3.8642	318.1162	4.450713	Tryptophan alkaloids	Alkaloids	SUYSEAZIUVYHDG
**349**	3.6016	133.076	0.853494	Tryptophan alkaloids	Alkaloids	LVRFTAZAXQPQHI
**1281**	3.5992	299.1026	2.486549	Anthranilic acid alkaloids	Alkaloids	KQPFLOCEYZIIRD

**2675**	3.5976	308.1655	4.4969	Tryptophan alkaloids	Alkaloids	PDWXCPOZTNNZCY
**811**	3.586	283.1045	1.579996	Tryptophan alkaloids	Alkaloids	YWBRXRXMXOEXLY
**753**	3.5387	203.0815	1.559964	Anthranilic acid alkaloids	Alkaloids	VASLDXYTSVTJKY
**2640**	3.4998	391.2041	4.44721	Phenolic acids (C6-C1)	Alkaloids	NTUPPVZJNOAYQQ
**1301**	3.3675	854.5027	2.504773	unknown	unknown	VCMQMWKGIHWSPX
**751**	3.1981	175.0865	1.561819	Nicotinic acid alkaloids	Alkaloids	VBHFPIWVTVHWES
**1276**	3.1777	389.1043	2.477474	Monoterpenoids	Terpenoids	CJDAIJHZTKDLTJ
**3209**	3.1647	318.2067	5.151517	Tryptophan alkaloids	Alkaloids	BJEPYKJPYRNKOW
**924**	3.122	275.0305	1.800976	Anthranilic acid alkaloids	Alkaloids	GRAKHGFVPASAHI
**716**	3.1028	190.0862	1.505281	Small peptides	Shikimates and Phenylpropanoids	KTHADMDGDNYQRX
**662**	3.0416	511.062	1.39819	Pseudoalkaloids	Alkaloids	CRZOICRNCTVVDV
**1050**	3.0179	201.0658	2.071038	Nicotinic acid alkaloids	Alkaloids	UMNZUDYDIKGADO
**823**	2.9481	294.124	1.674213	Anthranilic acid alkaloids	Alkaloids	YMNQPZJRJJQFBM
**645**	2.9232	170.1174	1.389183	Small peptides	Amino acids and Peptides	KLYKJOPFNDDFNE
**3493**	2.7948	325.236	5.485218	Diterpenoids	Fatty acids	CXJHSAYGYXEWQG
**1305**	2.7211	290.139	2.517695	Anthranilic acid alkaloids	Alkaloids	BFSBNVPBVGFFCF
**798**	2.6987	227.082	1.617997	unknown	unknown	XNXQVRHXDIDGDT
**1054**	2.5421	219.1215	2.09042	Pseudoalkaloids	Alkaloids	CZCIKBSVHDNIDH
**946**	2.5335	204.0849	1.890397	Pseudoalkaloids	Alkaloids	FTTGAAZKBNZDCZ
**3105**	2.5182	356.2219	4.9654	Macrolides	Alkaloids	JNVITAJZSSMIIU
**405**	2.4981	193.1335	0.97786	Tryptophan alkaloids	Alkaloids	AVIMCWMLFNLXJX
**1163**	2.4856	266.0955	2.280707	Lysine alkaloids	Alkaloids	ZSJLQEPLLKMAKR
**881**	2.4731	238.0965	1.770143	unknown	unknown	NWPWVFAEENVVJM
**1012**	2.4716	118.065	1.996237	unknown	unknown	SUSQOBVLVYHIEX
**950**	2.3635	203.0814	1.88991	Anthranilic acid alkaloids	Alkaloids	LKYWXXAVLLVJAS
**664**	2.3432	211.0875	1.409439	Small peptides	Alkaloids	YNCMLFHHXWETLD
**2264**	2.3242	560.3928	3.945279	Tryptophan alkaloids	Alkaloids	JFKCGYLVIVJKAH
**970**	2.3044	238.0732	1.910798	Nicotinic acid alkaloids	Alkaloids	BIGFZLZJHGMGPI
**2193**	2.2783	539.3639	3.83919	Anthranilic acid alkaloids	Alkaloids	CIYLRXJOTVCFNF

**969**	2.2377	301.0641	1.909407	Anthranilic acid alkaloids	Alkaloids	MHJJLGCCASIGNO
**649**	2.2	212.0905	1.404476	Small peptides	Amino acids and Peptides	WCSXQBAXPUYDDV
**981**	2.1775	216.1018	1.934352	Anthranilic acid alkaloids	Alkaloids	UHIBDZZBBOUXBF
**2376**	2.1646	586.408	4.111086	Linear polyketides	Polyketides	unknown
**855**	2.1601	238.074	1.736437	Small peptides	Amino acids and Peptides	LMCANDWMUCRYHX
**2547**	2.1485	326.1742	4.337158	Anthranilic acid alkaloids	Alkaloids	CXPGERDAOAFZJT
**2924**	2.0781	302.2115	4.77604	Oligopeptides	Alkaloids	AYCZWTQJTQZKHF
**2693**	1.9945	328.1908	4.515947	Tryptophan alkaloids	Alkaloids	BMNMZWYECBBAJT
**3090**	1.9815	340.2634	4.953427	Nicotinic acid alkaloids	Alkaloids	GXRKDSSARDBYHW
**2280**	1.9679	302.1745	3.978308	Linear polyketides	Alkaloids	OIPFXMLUWNAVJK
**2045**	1.9377	543.3584	3.64515	Diterpenoids	Terpenoids	UAMWSMPMWGFABN
**891**	1.9362	342.1442	1.788989	Tryptophan alkaloids	Alkaloids	GXKCUFUYTFWGNK
**1133**	1.8771	179.0602	2.219057	Pseudoalkaloids	Alkaloids	SQMDJKFNRLCHMW
**2722**	1.8497	302.2114	4.566289	Tryptophan alkaloids	Alkaloids	WVBVVWIGEPISMW
**1697**	1.8383	128.0528	3.14233	Lysine alkaloids	Alkaloids	CMOIEFFAOUQJPS
**2925**	1.7485	120.0444	4.784372	Anthranilic acid alkaloids	Amino acids and Peptides	RWZYAGGXGHYGMB
**2272**	1.701	577.3913	3.95498	unknown	unknown	WDFWQGCNPVLXKK
**2532**	1.6928	375.2102	4.318599	Ornithine alkaloids	Alkaloids	YTSDPGXQZGDXTJ
**2046**	1.6925	326.1748	3.64789	Tryptophan alkaloids	Alkaloids	KZEICARMWDZDDS
**2530**	1.6605	302.1211	4.318012	Tryptophan alkaloids	Alkaloids	IMXSCCDUAFEIOE
**2804**	1.6431	681.2722	4.662077	Oligopeptides	Amino acids and Peptides	XMDNHTUCEGYMFQ
**2159**	1.5718	563.362	3.815445	unknown	unknown	PYXZGBVSQBXPDQ
**1522**	1.5664	178.0318	2.892573	Phenolic acids (C6-C1)	Shikimates and Phenylpropanoids	LOGPRZMQSREDOU
**2133**	1.5225	275.1832	3.839055	Nicotinic acid alkaloids	Alkaloids	JOTAOFMUQJKRMS
**1985**	1.5219	178.0495	3.559725	unknown	unknown	RVWZUOPFHTYIEO
**2373**	1.5126	286.2163	4.169992	Anthranilic acid alkaloids	Alkaloids	TXPWUMZDOQMDFF
**1016**	1.4393	274.1438	1.998376	Tryptophan alkaloids	Alkaloids	MNIVLQOKRHODTM
**1941**	1.4117	584.2335	3.481179	Oligopeptides	Alkaloids	QOVMGOYVZMBYEY
**1137**	1.3983	181.0764	2.221395	Nicotinic acid alkaloids	Alkaloids	WQZGKKKJIJFFOK

**2710**	1.3696	312.1968	4.543877	Tryptophan alkaloids	Alkaloids	AWAMJTQSWCOGDM
**2933**	1.3084	679.2567	4.798267	Monoterpenoids	Alkaloids	GLAJZJRWMNNYEH
**1135**	1.2013	180.0685	2.219905	Pseudoalkaloids	Alkaloids	POAXUNDIOGWQOC
**2381**	1.1727	570.4137	4.116888	Pseudoalkaloids	Terpenoids	JYXWNSQXDWWFEW
**1932**	1.1641	437.095	3.476878	Anthranilic acid alkaloids	Alkaloids	CXNXZZJPLGLUHA
**2801**	1.1338	637.2171	4.661598	ÃŽÂ²-lactams	Amino acids and Peptides	IJMVASGWKRPVHU
**2802**	1.123	636.2136	4.660355	Tyrosine alkaloids	Polyketides	unknown
**2785**	1.1216	949.4177	4.638897	unknown	unknown	MOAREQZIZKPJBU
**2157**	1.1216	543.3943	3.827459	Steroids	Terpenoids	ZPTPAHYXDZSFJO
**497**	1.0651	110.06	1.125485	Small peptides	Amino acids and Peptides	CDAWCLOXVUBKRW
**2460**	1.0619	258.1854	4.224319	Tryptophan alkaloids	Alkaloids	JMNXQKCZLWIUSQ
**854**	1.0245	200.107	1.80283	Anthranilic acid alkaloids	Alkaloids	ANAVOAVCCMJUHE
**mexPQ-opmE increased features (vs PA14Δ4mex)** log2(FC) > 1 n= 52						
**ID**	**log2(FC)**	**row m/z**	**RT**	**NPC superclass**	**NPC pathway**	**Candidate inchikey**
**988**	8.1225	259.0537	1.951714	Nicotinic acid alkaloids	Alkaloids	WFHIVHVSLQQGHS
**1621**	6.1302	315.1679	3.038101	unknown	unknown	WSFQKSIBZODGPB
**1210**	5.8138	345.0341	2.370744	Serine alkaloids	Alkaloids	SBHXYTNGIZCORC
**1625**	5.1056	293.1862	3.038829	Peptide alkaloids	Alkaloids	WUPLEXBYYUOWQF
**1272**	4.418	235.0542	2.490227	Tryptophan alkaloids	Alkaloids	XLIQJCUAXROFGQ
**1118**	4.3223	221.0379	2.190301	Anthranilic acid alkaloids	Alkaloids	YCFFMSOLUMRAMD
**4492**	4.2003	358.1837	6.406226	Macrolides	Terpenoids	XCFSBOSFMAOQAL
**3375**	3.9897	541.3332	5.344727	Anthranilic acid alkaloids	Fatty acids	YIOSSWUAQNGRSJ
**1385**	3.7373	323.0515	2.631801	Serine alkaloids	Alkaloids	HZCUBLLQCILJTN
**2675**	3.7233	308.1655	4.4969	Tryptophan alkaloids	Alkaloids	PDWXCPOZTNNZCY
**1700**	3.3313	172.0422	3.142551	Fatty acyls	Fatty acids	IDAICLIJTRXNER
**1711**	3.1821	100.0214	3.14365	Fatty acyls	Fatty acids	VZWOXDYRBDIHMA
**924**	3.1117	275.0305	1.800976	Anthranilic acid alkaloids	Alkaloids	GRAKHGFVPASAHI
**1090**	2.9727	314.0953	2.158781	Anthranilic acid alkaloids	Alkaloids	BHXCFNZULGNWRA
**1308**	2.9255	413.1642	2.523094	Small peptides	Amino acids and Peptides	DZSKDBWTXGMINH
**1707**	2.9207	329.058	3.141756	Monoterpenoids	Alkaloids	GRCODZNXJRUTGU

**1046**	2.8503	196.0428	2.059265	Serine alkaloids	Alkaloids	QBBKPPKSBCAJHK
**1008**	2.8325	226.0534	1.991059	Anthranilic acid alkaloids	Alkaloids	LQQUFKZMSYJJGW
**1697**	2.7994	128.0528	3.14233	Lysine alkaloids	Alkaloids	CMOIEFFAOUQJPS
**1844**	2.7963	292.1542	3.368793	Linear polyketides	Fatty acids	LJFWHSLCSLLNMJ
**1213**	2.7889	323.0517	2.368219	Serine alkaloids	Alkaloids	HZCUBLLQCILJTN
**1703**	2.6404	702.0377	3.137458	unknown	unknown	unknown
**1445**	2.6071	427.1794	2.750743	Small peptides	Amino acids and Peptides	HFBASMIVABLIAD
**2410**	2.5875	328.1898	4.128937	Fatty amides	Fatty acids	UMVKTYCOYYLLPX
**3209**	2.5076	318.2067	5.151517	Tryptophan alkaloids	Alkaloids	BJEPYKJPYRNKOW
**1702**	2.4807	146.028	3.138673	unknown	unknown	DMGKNZBGIHCAMP
**1710**	2.4352	178.032	3.138597	Phenolic acids (C6-C1)	Shikimates and Phenylpropanoids	LOGPRZMQSREDOU
**1362**	2.3812	190.0323	2.617182	Small peptides	Alkaloids	unknown
**881**	2.322	238.0965	1.770143	unknown	unknown	NWPWVFAEENVVJM
**1701**	2.3172	279.0626	3.143205	Anthranilic acid alkaloids	Alkaloids	SKYXJWUTGOPZDA
**2068**	2.3083	336.2286	3.679403	Tryptophan alkaloids	Alkaloids	XPZFMHCHEWYIGE
**1359**	2.2923	208.0428	2.619498	Serine alkaloids	Alkaloids	BOSGDCHVRSIZSY
**2196**	2.2561	276.1869	3.863899	Peptide alkaloids	Alkaloids	SJIMDGIDDDGXLI
**3770**	2.165	395.2774	5.755904	unknown	Fatty acids	YJSXTLYNFBFHAT
**716**	2.1647	190.0862	1.505281	Small peptides	Shikimates and Phenylpropanoids	KTHADMDGDNYQRX
**1349**	2.1502	341.0625	2.594136	Serine alkaloids	Alkaloids	AMPDZVASNOBSQZ
**2303**	2.0745	326.1766	4.002193	Linear polyketides	Polyketides	YNEOQMQDVSEXCP
**1522**	1.9224	178.0318	2.892573	Phenolic acids (C6-C1)	Shikimates and Phenylpropanoids	LOGPRZMQSREDOU
**1524**	1.6577	378.9856	2.891751	Histidine alkaloids	Alkaloids	MUEMJABCTIXBIW
**1872**	1.633	302.1756	3.38525	Anthranilic acid alkaloids	Alkaloids	FMGBNISRFNDECK
**2291**	1.6079	275.1834	3.916886	Nicotinic acid alkaloids	Alkaloids	JOTAOFMUQJKRMS
**2722**	1.5048	302.2114	4.566289	Tryptophan alkaloids	Alkaloids	WVBVVWIGEPISMW
**2133**	1.4801	275.1832	3.839055	Nicotinic acid alkaloids	Alkaloids	JOTAOFMUQJKRMS
**2785**	1.4554	949.4177	4.638897	unknown	unknown	MOAREQZIZKPJBU
**3513**	1.3223	536.3796	5.490673	Fatty Acids and Conjugates	Fatty acids	JMNXFZCXVGEDAC

**2103**	1.3195	631.3736	3.722984	Steroids	Terpenoids	GNASEHLLVVEFHT
**2373**	1.2919	286.2163	4.169992	Anthranilic acid alkaloids	Alkaloids	TXPWUMZDOQMDFF
**2046**	1.2588	326.1748	3.64789	Tryptophan alkaloids	Alkaloids	KZEICARMWDZDDS
**2096**	1.1961	346.2008	3.717979	Tryptophan alkaloids	Alkaloids	JQYPNSFOJGBSOS
**2623**	1.174	625.4365	4.422786	Linear polyketides	Fatty acids	DYXMGEHQMTZPKJ
**2500**	1.1416	303.2156	4.28352	Fatty Acids and Conjugates	Fatty acids	GTJOQTFPQLBALB
**2716**	1.0127	314.2114	4.538503	Anthranilic acid alkaloids	Alkaloids	LWYHXEXQBAUDEY
**PA14Δ4mex increased or decreased features (vs PA14)** - total n = 362, log2(FC) > 1 n= 185, log2(FC) < 1 n= 177						
**ID**	**log2(FC)**	**row m/z**	**RT**	**NPC superclass**	**NPC pathway**	**Candidate inchikey**
**5514**	12.276	617.3961	7.498384	Pseudoalkaloids (transamidation)	unknown	PHEDXBVPIONUQT
**908**	10.817	459.2137	1.842093	Small peptides	Amino acids and Peptides	KRKNYBCHXYNGOX
**5763**	10.217	685.4609	7.937309	Fatty amides	unknown	DQQFPJYHEYSNAS
**679**	9.6694	233.0686	1.433995	Nicotinic acid alkaloids	Alkaloids	OIURYJWYVIAOCW
**2664**	9.4381	444.1575	4.478004	Tryptophan alkaloids	Alkaloids	OZRNSSUDZOLUSN
**5455**	9.3336	527.3507	7.425827	Ornithine alkaloids	Terpenoids	KARAUHIKECAWGB
**5611**	9.3269	655.4336	7.67671	Pseudoalkaloids	unknown	GORGDRGXUKJXOM
**5823**	9.3191	583.4142	8.061977	Macrolides	unknown	ATZSDDALXBKOQD
**2841**	9.2786	404.1272	4.694898	Coumarins	Shikimates and Phenylpropanoids	TVJHJRXOIAMNSY
**788**	9.1217	443.1829	1.625829	Histidine alkaloids	Alkaloids	OZVCWTRKMWKHHC
**628**	9.0365	233.0687	1.372241	unknown	unknown	OIURYJWYVIAOCW
**2748**	9.0217	406.1379	4.584912	Small peptides	Amino acids and Peptides	ULBLUJBIDCYVTD
**2020**	9.0055	261.0636	3.639777	Small peptides	Alkaloids	ZLOIGESWDJYCTF
**2093**	8.8192	261.0633	3.690501	Anthranilic acid alkaloids	Alkaloids	ZLOIGESWDJYCTF
**653**	8.8029	237.1023	1.412138	Anthranilic acid alkaloids	Alkaloids	JOMFHFOOPCCBLZ
**496**	8.6999	212.0907	1.106677	Small peptides	Alkaloids	WCSXQBAXPUYDDV
**1068**	8.5353	439.2334	2.157894	Small peptides	Amino acids and Peptides	OXYNOEAWWVRXNO
**1900**	8.1717	419.1493	3.477637	Pseudoalkaloids	Alkaloids	KQPFLOCEYZIIRD
**2363**	7.9535	342.127	4.072987	Anthranilic acid alkaloids	Alkaloids	SFLGFRJGKHRRID
**5897**	7.9487	597.4296	8.285293	Ornithine alkaloids	unknown	DFOYVSCLGRVGJK

**280**	7.865	476.0925	0.77381	Flavonoids	Alkaloids	ZRTFDOHHKNWDGO
**2480**	7.8191	423.0922	4.248278	Anthranilic acid alkaloids	Alkaloids	ITPHOIFCAFNCLL
**5729**	7.7915	568.3892	7.877055	Carotenoids (C40)	Fatty acids	KOARRXOZPWLCJM
**1481**	7.7805	316.108	2.829854	Anthranilic acid alkaloids	Alkaloids	ODTCNJWUFHCFJB
**1566**	7.6772	210.0754	2.948294	Pseudoalkaloids	Alkaloids	MYVUDDGZMPQJEZ
**5639**	7.642	553.3654	7.712562		Terpenoids	AKDLLCSTRRAVPQ
**1263**	7.6237	391.1194	2.472725	Nicotinic acid alkaloids	Alkaloids	BZOJTNLXPLGYIH
**1491**	7.5628	267.1126	2.85981	Tryptophan alkaloids	Alkaloids	NHLSUHWCECGWQN
**626**	7.5343	209.0712	1.372156	Nicotinic acid alkaloids	Alkaloids	CDICDSOGTRCHMG
**4959**	7.5222	485.3037	6.90748	Nicotinic acid alkaloids	Alkaloids	KKMHFUKZHJOMJL
**1261**	7.4859	168.0682	2.476267	unknown	unknown	RADKZDMFGJYCBB
**762**	7.4147	237.1022	1.504321	Anthranilic acid alkaloids	Alkaloids	ADIPDRPBEZELRZ
**1971**	7.3687	447.1439	3.534045	Nicotinic acid alkaloids	Alkaloids	YCRUGMHLZHDDGT
**1123**	7.1536	209.0711	2.200805	Tryptophan alkaloids	Alkaloids	UEHCMQFOWIRZGQ
**1256**	7.1093	359.1484	2.456443	unknown	unknown	FIAHDCXBPGRVEK
**1350**	7.0588	198.0762	2.543064	Small peptides	Amino acids and Peptides	BAWFJGJZGIEFAR
**306**	7.0577	513.0601	0.787106	Anthranilic acid alkaloids	Polyketides	OSALNYNYADJXIW
**2511**	7.0121	624.1779	4.296814	Oligopeptides	Amino acids and Peptides	UCXVISAAKKHFGU
**2419**	6.9371	344.0886	4.160383	Nicotinic acid alkaloids	Alkaloids	ZXSJQYOLQMMIHI
**2523**	6.9238	461.161	4.310133	Anthranilic acid alkaloids	Alkaloids	UMCSSUSEJCLNON
**1075**	6.9191	332.1035	2.116537	Anthranilic acid alkaloids	Alkaloids	DHEYNOAUAOKCSU
**1274**	6.9082	198.0762	2.478336	Small peptides	Amino acids and Peptides	BAWFJGJZGIEFAR
**5808**	6.8896	683.4638	8.000899	Glycerophospholipids	Alkaloids	IULXNECTRWHKDM
**3280**	6.8837	894.3502	5.208419	unknown	unknown	
**2622**	6.7507	241.0978	4.430805	Tryptophan alkaloids	Alkaloids	WFYLTIMBLPIRRU
**1363**	6.7302	316.1079	2.618036	Anthranilic acid alkaloids	Alkaloids	NUWZAJDJERBAHJ
**2665**	6.6693	445.164	4.476369	Anthranilic acid alkaloids	Alkaloids	NWXMGUDVXFXRIG
**886**	6.6254	283.0683	1.773104		unknown	YPYRMWFNCMDUIB
**2468**	6.6153	330.1224	4.214034	Tryptophan alkaloids	Alkaloids	CVKLXIIZBOVUOL
**1276**	6.5743	389.1043	2.477474	Monoterpenoids	Terpenoids	CJDAIJHZTKDLTJ

**823**	6.553	294.124	1.674213	Anthranilic acid alkaloids	Alkaloids	YMNQPZJRJJQFBM
**1765**	6.5046	379.1195	3.210676	Nicotinic acid alkaloids	Alkaloids	HXAZTNIVBVVFJS
**1574**	6.4225	242.1026	2.963404	Pseudoalkaloids	Alkaloids	AKOVXPNRITVXAR
**2577**	6.4214	437.1059	4.376672	Tryptophan alkaloids	Alkaloids	XRTJEHUGXNDQHW
**1571**	6.4109	209.0709	2.949258	Anthranilic acid alkaloids	Alkaloids	GTPAIRPVSKCYST
**2614**	6.343	209.0706	4.411646	Nicotinic acid alkaloids	Alkaloids	CFKDNVWYKYSRQV
**2698**	6.3011	486.1851	4.518549	Tryptophan alkaloids	Alkaloids	BQNGWUKHPUZJBE
**1351**	6.2671	338.0908	2.612775	unknown	unknown	UIEZBUHEBCJAMR
**260**	6.2319	241.0969	0.721952	Anthranilic acid alkaloids	Alkaloids	WFYLTIMBLPIRRU
**766**	6.1239	255.0767	1.596105	Anthranilic acid alkaloids	Alkaloids	INPMVLIHPFWVLB
**301**	6.0797	511.0619	0.789166	Nucleosides	Alkaloids	CRZOICRNCTVVDV
**2191**	6.0749	419.1513	3.846101	Anthranilic acid alkaloids	Alkaloids	KQPFLOCEYZIIRD
**5726**	6.0618	567.3822	7.873022	Steroids	unknown	GLIPEBQBWMVZJH
**5559**	6.0383	541.3662	7.561037	Small peptides	Polyketides	JRFQUOXEKACVEL
**3730**	6.0051	415.2257	5.70957	unknown	Alkaloids	OFKABAMHUKOKMV
**1601**	5.8117	435.1439	3.004781	Peptide alkaloids	Alkaloids	WQKKQBMRVKDLBP
**1269**	5.7686	169.0763	2.475649	Anthranilic acid alkaloids	Alkaloids	AIFRHYZBTHREPW
**2587**	5.7319	434.1345	4.368959	Tryptophan alkaloids	Alkaloids	NFKKAYYYJYWZMD
**1856**	5.6116	239.0813	3.395597	Anthranilic acid alkaloids	Alkaloids	FBMQNRKSAWNXBT
**2099**	5.5942	239.0814	3.697787	Anthranilic acid alkaloids	Alkaloids	ICWZJHDBAPKRIX
**1864**	5.561	549.3327	3.388665	Pseudoalkaloids	Terpenoids	NETARJWZTMGMRM
**5727**	5.5545	569.3984	7.873217		Terpenoids	HICFZYXWKMYSPM
**2870**	5.5418	433.1308	4.690584	Anthranilic acid alkaloids	Alkaloids	DNOIKCRYNMLUFP
**707**	5.5157	229.0607	1.494589	Anthranilic acid alkaloids	Alkaloids	PRLQJFSSCAIOOO
**5725**	5.5131	570.4013	7.876262	unknown	Terpenoids	MAIAZYOVLDFHJR
**3180**	5.4515	514.2165	5.118917	Pseudoalkaloids	Alkaloids	AKEOLENLJFVVTD
**875**	5.3731	346.118	1.740064	Anthranilic acid alkaloids	Alkaloids	YXNIEZJFCGTDKV
**1166**	5.2999	223.0531	2.286524	Small peptides	Alkaloids	YIVWQNVQRXFZJB
**1257**	5.2393	337.1658	2.456235	Anthranilic acid alkaloids	Alkaloids	XZIJBWJIVVWFHL

**1504**	5.2023	379.1196	2.876593	Chromanes	Shikimates and Phenylpropanoids	JPIKFLJADUHSCV
**1560**	5.1917	239.0817	2.939806	Anthranilic acid alkaloids	Alkaloids	BMSXFAVRACGWDQ
**2679**	5.1679	316.1908	4.498109	Tryptophan alkaloids	Alkaloids	LTXRLUQBZWBCGH
**684**	5.1654	234.0724	1.45093	Nicotinic acid alkaloids	Alkaloids	QFBRVVIIHBQBQA
**1043**	5.165	209.0709	2.051397	unknown	unknown	FIQPIEYTJUGXHH
**1113**	5.1454	323.1493	2.179496	Tryptophan alkaloids	Alkaloids	CFQJLIHMLCPJQF
**1868**	5.0971	290.1748	3.392793	Anthranilic acid alkaloids	Alkaloids	RKUNBYITZUJHSG
**1392**	5.064	431.1724	2.657491	Small peptides	Alkaloids	KDTTYUOOJRPMSG
**751**	5.0539	175.0865	1.561819	Nicotinic acid alkaloids	Alkaloids	VBHFPIWVTVHWES
**5686**	4.9494	809.5475	7.803451	Glycerolipids	Fatty acids	BOEMOMZYCWHUMI
**1543**	4.9478	455.1354	2.911093	Anthranilic acid alkaloids	Alkaloids	DCKDCCDCRYVECG
**1277**	4.9174	214.0741	2.482422	Lysine alkaloids	Alkaloids	RTOWSIGTGIANGZ
**718**	4.8912	251.0426	1.510863	Nicotinic acid alkaloids	Alkaloids	XOAAWQZATWQOTB
**3003**	4.8755	421.178	4.871006	Saccharides	Alkaloids	QEUHGZGGGPRQOJ
**4332**	4.8351	459.2503	6.238798	Small peptides	Alkaloids	AEULNKPXALBIHK
**1242**	4.8064	306.1247	2.435274	Tryptophan alkaloids	Alkaloids	OGSAEMMXCPDVRH
**1579**	4.7207	241.0971	2.963403	Anthranilic acid alkaloids	Alkaloids	DKHBOZGVMLZLLL
**2486**	4.7164	209.0709	4.266103	Tryptophan alkaloids	Alkaloids	AIFNAMVERSBWPS
**414**	4.6349	350.1139	0.988581	Tryptophan alkaloids	Alkaloids	AVKUERGKIZMTKX
**685**	4.6043	255.0762	1.47197	Anthranilic acid alkaloids	Alkaloids	JIEZGIQZGBBYFW
**4813**	4.5765	471.2878	6.700851	Fatty esters	Terpenoids	QORZOJLEBCJYBI
**1130**	4.55	237.1019	2.201792	unknown	unknown	ADIPDRPBEZELRZ
**2610**	4.4971	433.1288	4.405226	Tryptophan alkaloids	Alkaloids	JRFVOMWWESIGRR
**1265**	4.4725	413.1014	2.473548		unknown	FRKARNRSYLXVBE
**4329**	4.4441	458.2431	6.23536	Steroids	Alkaloids	RWSVCNGLTCIUJD
**5636**	4.3502	555.3822	7.708763	Fatty amides	unknown	XSWBRSHUFLPEOV
**1858**	4.3427	283.1064	3.369496	Nicotinic acid alkaloids	Alkaloids	ROPTVRLUGSPXNH
**1357**	4.2834	314.092	2.624426	unknown	unknown	ZUUILEHAHVINQF
**1271**	4.2408	197.071	2.476021	Anthranilic acid alkaloids	Alkaloids	JGCSKOVQDXEQHI

**1482**	4.2398	314.0926	2.832523	Anthranilic acid alkaloids	Alkaloids	GNTVWGDQPXCYBV
**1007**	4.2089	332.1031	1.988594	Anthranilic acid alkaloids	Alkaloids	ROYNNIHWGRMUAU
**2602**	4.1932	211.0866	4.384463	Anthranilic acid alkaloids	Alkaloids	YNCMLFHHXWETLD
**2654**	4.191	432.1217	4.464018	Tryptophan alkaloids	Alkaloids	BPZZWRPHVVDAPT
**3111**	4.1569	446.1739	4.981899	Diterpenoids	Alkaloids	MSTNYGQPCMXVAQ
**727**	4.1245	110.0599	1.503099	Nicotinic acid alkaloids	Alkaloids	CDAWCLOXVUBKRW
**1195**	4.0096	318.0853	2.330277	Anthranilic acid alkaloids	Alkaloids	VUKAQXDGBZWYGE
**493**	3.9764	205.061	1.122421	Nicotinic acid alkaloids	Alkaloids	XIIQRVUIEJEQQV
**664**	3.9674	211.0875	1.409439	Small peptides	Alkaloids	YNCMLFHHXWETLD
**2796**	3.9489	314.0931	4.65495	Anthranilic acid alkaloids	Alkaloids	FFXSZPYLDUQMNY
**1141**	3.9274	246.0639	2.222326	Nicotinic acid alkaloids	Alkaloids	OWLBYSCPSVERBE
**1137**	3.8815	181.0764	2.221395	Nicotinic acid alkaloids	Alkaloids	WQZGKKKJIJFFOK
**1040**	3.858	227.0817	2.041732	Lysine alkaloids	Alkaloids	XNXQVRHXDIDGDT
**4331**	3.8067	441.2418	6.237959	Lysine alkaloids	Alkaloids	UPNKNNIPZSHPKK
**2584**	3.802	239.0817	4.371906	Anthranilic acid alkaloids	Alkaloids	UEHCMQFOWIRZGQ
**1477**	3.7942	338.09	2.82874	Anthranilic acid alkaloids	Alkaloids	ZTPHWMRCXGOIFZ
**1754**	3.7807	329.1937	3.204774	Pseudoalkaloids	Alkaloids	OLOQSHYLOVYRGK
**918**	3.7768	219.0764	1.834632	Anthranilic acid alkaloids	Alkaloids	IUQZLPOODRVXJP
**4042**	3.7762	429.2402	5.975014	Meroterpenoids	Terpenoids	BCWSYPDADKVYMB
**520**	3.7456	211.0867	1.120894	unknown	unknown	YNCMLFHHXWETLD
**3921**	3.7336	328.1084	5.878076	Ornithine alkaloids	Alkaloids	OJUJNNKCVPCATE
**1358**	3.7257	197.071	2.550522	Anthranilic acid alkaloids	Alkaloids	RGHNJXZEOKUKBD
**494**	3.7244	161.071	1.120431	Nicotinic acid alkaloids	Alkaloids	FIEYHAAMDAPVCH
**1266**	3.723	392.1234	2.473435	Peptide alkaloids	Alkaloids	VHIRZEOVXUFNRW
**4318**	3.7195	443.2571	6.228893	Steroids	Terpenoids	RKHVHPLYSACKRB
**1239**	3.6712	304.1085	2.434447	Nicotinic acid alkaloids	Alkaloids	BTWZMPXQZXWWGU
**1478**	3.6635	318.1178	2.828935	Nicotinic acid alkaloids	Alkaloids	SUYSEAZIUVYHDG
**1262**	3.6535	179.0606	2.476656	Small peptides	Shikimates and Phenylpropanoids	SQMDJKFNRLCHMW
**291**	3.6058	212.0903	0.775749	Pseudoalkaloids	Alkaloids	GDPVGSOBUOFHQM

**1562**	3.5944	207.0552	2.877191	unknown	unknown	YNOXCRMFGMSKIJ
**2530**	3.5829	302.1211	4.318012	Tryptophan alkaloids	Alkaloids	IMXSCCDUAFEIOE
**292**	3.5129	211.0865	0.777561	Anthranilic acid alkaloids	Alkaloids	YNCMLFHHXWETLD
**1561**	3.5034	240.0885	2.938311	Anthranilic acid alkaloids	Alkaloids	IQFWYNFDWRYSRA
**649**	3.4799	212.0905	1.404476	Small peptides	Amino acids and Peptides	WCSXQBAXPUYDDV
**518**	3.452	443.1362	1.16162	Anthranilic acid alkaloids	Alkaloids	FOPKJWLSORSOOZ
**2943**	3.4446	512.2007	4.816349	Ornithine alkaloids	Alkaloids	ZBQJUUDIPOFIAD
**912**	3.4278	237.1022	1.782098	Anthranilic acid alkaloids	Alkaloids	ADIPDRPBEZELRZ
**1302**	3.4107	244.0805	2.5124	Pseudoalkaloids	Alkaloids	INPMVLIHPFWVLB
**1708**	3.4016	439.1394	3.140547	Tryptophan alkaloids	Alkaloids	DEBRBEDKRGQAPL
**431**	3.3692	233.0685	1.023849	Nicotinic acid alkaloids	Alkaloids	WHRFJAWYQNMTIP
**2477**	3.3355	612.4255	4.236771	Steroids	Terpenoids	unknown
**1352**	3.335	224.0819	2.537656	Anthranilic acid alkaloids	Alkaloids	KPZYYKDXZKFBQU
**1310**	3.3201	264.1019	2.526132	Tryptophan alkaloids	Alkaloids	COBBNRKBTCBWQP
**3159**	3.3184	631.1549	5.065905	Tryptophan alkaloids	Alkaloids	NJCRSCPKMAXMKK
**1304**	3.286	243.0766	2.512921	Coumarins	Alkaloids	CUYPWOPOBAHCCE
**1135**	3.2639	180.0685	2.219905	Pseudoalkaloids	Alkaloids	POAXUNDIOGWQOC
**1126**	3.2435	207.0552	2.211717	Tryptophan alkaloids	Alkaloids	YNOXCRMFGMSKIJ
**1052**	3.2163	239.0812	2.084138	Anthranilic acid alkaloids	Alkaloids	ICWZJHDBAPKRIX
**2532**	3.18	375.2102	4.318599	Ornithine alkaloids	Alkaloids	YTSDPGXQZGDXTJ
**3090**	3.1519	340.2634	4.953427	Nicotinic acid alkaloids	Alkaloids	GXRKDSSARDBYHW
**2106**	3.1487	304.1909	3.714054	Anthranilic acid alkaloids	Alkaloids	RKSQYDBORLFRPF
**2590**	3.1288	261.0636	4.375292	Nicotinic acid alkaloids	Alkaloids	ZLOIGESWDJYCTF
**2561**	3.1132	431.1505	4.355466	Tryptophan alkaloids	Alkaloids	ZMQRJWIYMXZORG
**2487**	3.0909	312.117	4.263633	Anthranilic acid alkaloids	Alkaloids	MEXUTNIFSHFQRG
**1452**	3.0856	208.0591	2.710985	Pseudoalkaloids	Alkaloids	ZPZAOBSARYZTDT
**1270**	3.0531	196.0638	2.47611	Anthranilic acid alkaloids	Alkaloids	JDWYRSDDJVCWPB
**859**	3.0133	304.1077	1.74662	Anthranilic acid alkaloids	Alkaloids	OLMHRSLUDJARDO
**1355**	3.0049	208.0589	2.595199	unknown	unknown	STQUCJZSEVAMPY
**1129**	2.9741	224.0818	2.215751	Anthranilic acid alkaloids	Alkaloids	KPZYYKDXZKFBQU

**1290**	2.8962	248.1069	2.491734	Tryptophan alkaloids	Alkaloids	UTVNYKLBXWXEQP
**801**	2.8752	237.1023	1.587405	Anthranilic acid alkaloids	Alkaloids	ADIPDRPBEZELRZ
**1050**	2.8554	201.0658	2.071038	Nicotinic acid alkaloids	Alkaloids	UMNZUDYDIKGADO
**1429**	2.726	181.0765	2.721766	Nicotinic acid alkaloids	Alkaloids	WQZGKKKJIJFFOK
**2628**	2.6605	448.1525	4.43048	Tryptophan alkaloids	Alkaloids	VCRVBZKVBRQVRP
**2465**	2.5155	611.4207	4.234175	Anthranilic acid alkaloids	Alkaloids	FVCCCELSDGJJMK
**1131**	2.4734	226.0692	2.218845	Nicotinic acid alkaloids	Alkaloids	OIUJHGOLFKDBSU
**1333**	2.2449	247.0478	2.588806	unknown	unknown	UPMKELQKOTYAEP
**1140**	2.1751	225.0659	2.220247	Anthranilic acid alkaloids	Alkaloids	JGCSKOVQDXEQHI
**1133**	2.1277	179.0602	2.219057	Pseudoalkaloids	Alkaloids	SQMDJKFNRLCHMW
**3814**	2.0025	356.2944	5.82178	Peptide alkaloids	Fatty acids	UXBFKHXOHNPCCI
**2390**	1.9371	320.1985	4.121972	unknown	unknown	BJEPYKJPYRNKOW
**568**	1.8782	216.1019	1.241655	Tryptophan alkaloids	Alkaloids	SUHOQUVVVLNYQR
**2479**	1.7616	609.4059	4.247804		unknown	DYPQZGJEEBDGCP
**1128**	1.7592	225.0864	2.214275	Small peptides	Amino acids and Peptides	LQSKCSIAFZODHC
**1404**	1.692	207.0552	2.592366	Anthranilic acid alkaloids	Alkaloids	YNOXCRMFGMSKIJ
**2876**	1.3606	326.2477	4.725432	Anthranilic acid alkaloids	Alkaloids	YQPWHWKJONMUQU
**2388**	1.2916	298.2162	4.120736	Anthranilic acid alkaloids	Alkaloids	AWCWOJPATVETFJ
**2133**	-1.0431	275.1832	3.839055	Nicotinic acid alkaloids	Alkaloids	JOTAOFMUQJKRMS
**70**	-1.1918	164.093	0.460983	Pseudoalkaloids	Alkaloids	NTBYIQWZAVDRHA
**3256**	-1.2719	550.3944	5.208501	unknown	Fatty acids	JOPPVTHMSDTTEL
**3248**	-1.4258	527.3189	5.194675	Fatty acyl glycosides	Fatty acids	DPVZSCHCGANSMG
**2706**	-1.4345	439.2233	4.532158	Small peptides	Amino acids and Peptides	MDYSLOGZXCWLSL
**2045**	-1.5111	543.3584	3.64515	Diterpenoids	Terpenoids	UAMWSMPMWGFABN
**1800**	-1.5455	258.1853	3.2889	Anthranilic acid alkaloids	Alkaloids	JMNXQKCZLWIUSQ
**2365**	-1.5523	284.201	4.077424	Anthranilic acid alkaloids	Alkaloids	XICKFFMQHHMRFO
**2585**	-1.5555	284.1649	4.370188	Tryptophan alkaloids	Alkaloids	FNEYFRLVOWTCBP
**2069**	-1.5913	313.1523	3.679848	Peptide alkaloids	Alkaloids	MNIVLQOKRHODTM
**2574**	-1.6098	318.1698	4.36914	Anthranilic acid alkaloids	Alkaloids	CAWZYUQNVABYFN
**2339**	-1.6249	296.2008	4.055811	Anthranilic acid alkaloids	Alkaloids	QFIWUNYGZZMAFG

**2579**	-1.634	285.1715	4.370347	Monoterpenoids	Alkaloids	IHGGDNBNTURAAG
**2571**	-1.6468	302.1759	4.371143	Tryptophan alkaloids	Alkaloids	OROGSEYTTFOCAN
**4232**	-1.7164	217.1799	6.135396	Fatty acyl glycosides	Fatty acids	YDZIJQXINJLRLL
**3241**	-1.7346	359.2791	5.195636	Ornithine alkaloids	Fatty acids	ZFPAFAWFRTWCSK
**3259**	-1.7516	525.3724	5.198515	Pseudoalkaloids	Terpenoids	NRAFDWWUQUTQAC
**3855**	-1.7609	556.3541	5.832233	Ornithine alkaloids	Alkaloids	OLGSOPZIFZLCSE
**3273**	-1.7688	522.3631	5.195578	Octadecanoids	Terpenoids	FCBUKWWQSZQDDI
**3247**	-1.7715	505.3374	5.194842	unknown	Fatty acids	PPMPLIBYTIWXPG
**3270**	-1.7865	164.0919	5.198299	Nicotinic acid alkaloids	Fatty acids	NTBYIQWZAVDRHA
**2073**	-1.8049	147.0553	3.680765	Anthranilic acid alkaloids	Alkaloids	YJVOWRAWFXRESP
**3841**	-1.8253	107.0855	5.831025	Fatty Acids and Conjugates	Terpenoids	IVSZLXZYQVIEFR
**3600**	-1.8948	553.3352	5.553489	unknown	Polyketides	IEWRNTONLPUAQI
**2520**	-1.8977	391.2243	4.297929	Small peptides	Amino acids and Peptides	FDDUXCZCGGJQHT
**2075**	-1.8986	291.1703	3.681136	Small peptides	Alkaloids	WXEMUKVRWINVJS
**3269**	-1.9041	147.0602	5.197117	unknown	Alkaloids	YJVOWRAWFXRESP
**4225**	-2.0016	163.1481	6.132002	Fatty Acids and Conjugates	unknown	DDDUDPZNWNWAHI
**2362**	-2.0077	285.2043	4.073857	Fatty esters	Fatty acids	HYRMWBVOZFMSIF
**3612**	-2.0707	550.3863	5.553496	Fatty acyl glycosides	Alkaloids	JOPPVTHMSDTTEL
**2193**	-2.0936	539.3639	3.83919	Anthranilic acid alkaloids	Alkaloids	CIYLRXJOTVCFNF
**4221**	-2.1231	181.1587	6.130139	Glycerolipids	Fatty acids	XMPCQTDMPUUQFZ
**3461**	-2.1678	373.2949	5.491127	unknown	Fatty acids	AMRVOUCEFGMNBS
**466**	-2.1995	279.134	1.078264	Nucleosides	Alkaloids	UNFWWIHTNXNPBV
**1301**	-2.2521	854.5027	2.504773	unknown	unknown	VCMQMWKGIHWSPX
**4219**	-2.2548	199.1693	6.126437	Glycerolipids	Fatty acids	YDZIJQXINJLRLL
**3972**	-2.2664	443.3315	5.87584	Monoterpenoids	unknown	BLHLKJLSYHEOGY
**603**	-2.2705	343.13	1.287763	Fatty amides	Amino acids and Peptides	CZMRCDWAGMRECN
**2338**	-2.2797	178.0499	4.044773	Small peptides	Amino acids and Peptides	RVWZUOPFHTYIEO
**3174**	-2.3203	534.364	5.119965	Pseudoalkaloids	Alkaloids	PWQGBBFDEMPNKU
**3513**	-2.3349	536.3796	5.490673	Fatty Acids and Conjugates	Fatty acids	JMNXFZCXVGEDAC
**3026**	-2.3388	508.3482	4.889255	Phenolic acids (C6-C1)	Fatty acids	NSDCZUSAJCYTGH

**610**	-2.348	204.1248	1.332437	Small peptides	Alkaloids	HYVABZIGRDEKCD
**3820**	-2.3626	153.1274	5.829486	Fatty esters	unknown	CCEFMUBVSUDRLG
**3589**	-2.4258	1052.71	5.548275	unknown	unknown	KWGVNZWSEWAOMI
**3532**	-2.4288	519.353	5.493092	Fatty Acids and Conjugates	Fatty acids	FCBUKWWQSZQDDI
**645**	-2.433	170.1174	1.389183	Small peptides	Amino acids and Peptides	KLYKJOPFNDDFNE
**322**	-2.4718	296.1356	0.809871	Nucleosides	Carbohydrates	IEROEXCOOYBHPG
**4202**	-2.5142	387.3104	6.103026	Fatty Acids and Conjugates	Fatty acids	CARLURCVFKTIGN
**599**	-2.5146	191.0847	1.281584	Ornithine alkaloids	Alkaloids	RILHUWWTCSDPAN
**779**	-2.5166	242.0491	1.645156	Serine alkaloids	Alkaloids	QEGMHBAVHYBAMB
**3011**	-2.5302	171.1379	4.87986	Linear polyketides	Fatty acids	FYSSBMZUBSBFJL
**3018**	-2.5411	153.1274	4.880363	unknown	Fatty acids	CCEFMUBVSUDRLG
**2290**	-2.5485	541.3793	3.955278	Fatty amides	Fatty acids	OTNYCNDIGKWKTO
**3583**	-2.5616	619.4393	5.543712	Fatty Acids and Conjugates	unknown	IQZFGPJVZJWNJS
**1120**	-2.5942	383.1614	2.192065	Sesquiterpenoids	Terpenoids	VMSQKUCYEMOKMM
**3609**	-2.5988	551.3901	5.557076	Triterpenoids	unknown	BSBFCBHGWBTHOG
**2925**	-2.6141	120.0444	4.784372	Anthranilic acid alkaloids	Amino acids and Peptides	RWZYAGGXGHYGMB
**3903**	-2.6417	385.2947	5.811572	Fatty Acids and Conjugates	Polyketides	PPMPLIBYTIWXPG
**2675**	-2.6498	308.1655	4.4969	Tryptophan alkaloids	Alkaloids	PDWXCPOZTNNZCY
**2068**	-2.6919	336.2286	3.679403	Tryptophan alkaloids	Alkaloids	XPZFMHCHEWYIGE
**3024**	-2.7203	107.0854	4.894395	Macrolides	unknown	IVSZLXZYQVIEFR
**2340**	-2.7281	324.1566	4.046235	unknown	unknown	SHANZLCFUGOMJA
**2800**	-2.7463	351.2139	4.665263	unknown	unknown	IUDAFVFBMHXFER
**506**	-2.8522	193.0972	1.14341	Ornithine alkaloids	Alkaloids	WNKLVRDIRPADMZ
**3055**	-2.8738	369.2043	4.89874	Nicotinic acid alkaloids	Alkaloids	JTBMRNJFKYMXNT
**3343**	-2.8916	357.2627	5.269464	Triterpenoids	Fatty acids	VPRZXCNKOANCBI
**3530**	-2.925	341.2686	5.524953	Diterpenoids	Fatty acids	HPMCDJQPMNPVHR
**3582**	-2.9251	189.1485	5.544101	Diterpenoids	Fatty acids	FYSSBMZUBSBFJL
**4481**	-2.9744	435.3085	6.401498	Sesquiterpenoids	Fatty acids	MNGASGFOKSKGQQ
**3729**	-2.9788	490.373	5.721461	Diterpenoids	unknown	AHUPTCAANZMABA
**2926**	-3.0132	290.1756	4.783107	Small peptides	Alkaloids	RKUNBYITZUJHSG

**1114**	-3.056	231.1157	2.187523	Small peptides	Amino acids and Peptides	PLPBLFIRKVGHBJ
**509**	-3.0576	194.1007	1.140785	Pseudoalkaloids	Alkaloids	RITVBFZKAMDJHU
**3568**	-3.0884	135.1168	5.539627	Macrolides	unknown	KVNYFPKFSJIPBJ
**775**	-3.1094	239.0794	1.606517	Small peptides	Alkaloids	UGQMRVRMYYASKQ
**3455**	-3.1235	562.3978	5.490842	Diterpenoids	Amino acids and Peptides	unknown
**2047**	-3.1241	257.1748	3.632828	unknown	unknown	UNHVNJZHFJHEAM
**4263**	-3.1574	544.2727	6.166681	Diterpenoids	Polyketides	RONUKPQOBQKEHX
**994**	-3.1721	221.1284	1.971988	Lysine alkaloids	Alkaloids	UISQDUMVIZJYEI
**2410**	-3.1879	328.1898	4.128937	Fatty amides	Fatty acids	UMVKTYCOYYLLPX
**2484**	-3.1903	285.2045	4.206339	unknown	unknown	PWBGRXOGZIZZSS
**3521**	-3.2172	487.3614	5.519949	unknown	unknown	ITUGKYIZQDNKMM
**2358**	-3.2275	373.2571	4.049212	Guanidine alkaloids	Alkaloids	FVLJNWUXPRLMPE
**3588**	-3.2588	575.4131	5.525794	Linear polyketides	unknown	CJUDCLXYGJPHAJ
**3913**	-3.2843	407.2769	5.861825	Saccharides	Fatty acids	OEKUSRBIIZNLHZ
**4205**	-3.2889	409.2922	6.1159	Glycerolipids	Terpenoids	BHQCQFFYRZLCQQ
**3033**	-3.3035	1003.62	4.892698	Small peptides	unknown	unknown
**3042**	-3.304	125.096	4.907029	Fatty Acids and Conjugates	Fatty acids	BXRHTYIYAZHIHF
**2500**	-3.3056	303.2156	4.28352	Fatty Acids and Conjugates	Fatty acids	GTJOQTFPQLBALB
**3087**	-3.3128	853.4714	4.941095	Pseudoalkaloids	unknown	FGXIRJIGFSBQNF
**2567**	-3.3151	355.248	4.339195	Polyethers	Polyketides	ZEPMOPDHHDRDEK
**3563**	-3.3557	153.1274	5.53753	Pseudoalkaloids	Fatty acids	CCEFMUBVSUDRLG
**3564**	-3.4263	171.1379	5.534549	Fatty Acids and Conjugates	Fatty acids	FYSSBMZUBSBFJL
**4201**	-3.4885	404.3376	6.112749	Fatty acyl glycosides	Fatty acids	OQIJRBFRXGIHMI
**2451**	-3.5027	125.0961	4.250999	unknown	unknown	PCPJXSJZQTYXTL
**1204**	-3.5425	385.1771	2.347075	Meroterpenoids	Terpenoids	OLVSVPOPXGQLBV
**2049**	-3.6164	297.1676	3.649439	Fatty Acids and Conjugates	Fatty acids	SMXNLCYMHFJNLF
**4586**	-3.6589	544.2721	6.481457	Nicotinic acid alkaloids	Polyketides	RONUKPQOBQKEHX
**3088**	-3.7056	809.445	4.942673	unknown	Terpenoids	SKZTYAYWVMPFTB
**1823**	-3.7464	254.2483	3.327261	Sphingolipids	Fatty acids	YRPQTVNCCVPGFA
**3574**	-3.7616	911.6084	5.541386	unknown	unknown	

**3557**	-3.8308	359.2792	5.499894	Fatty Acids and Conjugates	Polyketides	ZFPAFAWFRTWCSK
**1205**	-3.841	233.1318	2.34736	Fatty amides	Fatty acids	HNZKRSKSIABLNS
**1001**	-3.9109	371.161	1.976042	Flavonoids	Shikimates and Phenylpropanoids	WGASWKHIQMYEHL
**3907**	-3.9122	121.1011	5.839583	Nicotinic acid alkaloids	Terpenoids	ZLCSFXXPPANWQY
**2470**	-3.9923	143.1067	4.245112	Fatty Acids and Conjugates	Fatty acids	TXXHDPDFNKHHGW
**2673**	-4.0952	457.1779	4.490413	Coumarins	Shikimates and Phenylpropanoids	AARICDGIAQPTML
**3770**	-4.1464	395.2774	5.755904	unknown	Fatty acids	YJSXTLYNFBFHAT
**2497**	-4.2539	115.0753	4.271053	unknown	unknown	CQJHAULYLJXJNL
**3559**	-4.3127	381.2614	5.52322	Fatty esters	Fatty acids	HVYBFBDAGMLZEH
**3902**	-4.3344	197.1535	5.835944	unknown	Fatty acids	ADZMLQKJPQFTIS
**4803**	-4.3811	438.3009	6.703179	Glycerolipids	Terpenoids	BLUHHIKWCCCMLQ
**1855**	-4.3829	337.2116	3.410284	Peptide alkaloids	Alkaloids	KUIJPSLAGAQZTC
**3211**	-4.4261	345.2636	5.131714	Anthranilic acid alkaloids	Fatty acids	YOMPSJJICGTGBM
**1861**	-4.5475	395.2545	3.390031	Eicosanoids	Amino acids and Peptides	CKDPEAINBFYEHJ
**2553**	-4.5759	298.2011	4.346155	Fatty amides	Fatty acids	PHSRRHGYXQCRPU
**4206**	-4.5803	795.5972	6.118484	Diterpenoids	unknown	WXGVDJKAEPVPOU
**3898**	-4.6645	179.143	5.84069	Steroids	unknown	KLTKQWRPJDRMTL
**3974**	-4.7113	554.3393	5.8856	unknown	Terpenoids	unknown
**3904**	-4.8409	939.6414	5.854365	Linear polyketides	unknown	unknown
**3074**	-4.8465	161.1172	4.918919	Nicotinic acid alkaloids	Fatty acids	APJYDQYYACXCRM
**2494**	-5.0024	325.1987	4.26857	Fatty Acids and Conjugates	Polyketides	YOSVFFVBSPQTTP
**3193**	-5.0155	367.245	5.150342	Anthranilic acid alkaloids	Fatty acids	OYOCQFXSNOJORV
**2496**	-5.0385	133.0859	4.270749	Fatty Acids and Conjugates	Fatty acids	LVRFTAZAXQPQHI
**1625**	-5.1463	293.1862	3.038829	Peptide alkaloids	Alkaloids	WUPLEXBYYUOWQF
**3857**	-5.1636	402.3227	5.856328	Pseudoalkaloids (transamidation)	unknown	PPMPLIBYTIWXPG
**4046**	-5.2032	381.2613	5.985807	Nicotinic acid alkaloids	Fatty acids	HVYBFBDAGMLZEH
**3046**	-5.233	143.1066	4.904306	Fatty Acids and Conjugates	Fatty acids	TXXHDPDFNKHHGW
**2498**	-5.3987	341.1736	4.271363	Tryptophan alkaloids	Alkaloids	IHISACBOLHQKCH
**3556**	-5.4062	376.3054	5.525034	unknown	Fatty acids	JZWLIRVAYJRWLN

**3045**	-5.4402	353.2299	4.90428	Fatty Acids and Conjugates	Fatty acids	KPQXJVQFJZUGRC
**1841**	-5.4858	936.4974	3.356499	unknown	unknown	PXGQQTUNCZXGMI
**3041**	-5.5533	331.2477	4.902814	Fatty acyls	Fatty acids	JHGVFGJXFVIYSM
**3035**	-5.6062	640.3906	4.895848	unknown	Polyketides	INNHOUBLAKKYHI
**2102**	-5.6269	352.173	3.722685	Ornithine alkaloids	Alkaloids	GDGIXKWOJJMLNP
**1865**	-5.715	296.1861	3.392579	Fatty amides	Fatty acids	DRDBTDHUTFJYHV
**3066**	-5.7978	313.2373	4.90437	Octadecanoids	Fatty acids	FIFLDFYODFCEFA
**2713**	-5.9006	339.2139	4.557355	Linear polyketides	Polyketides	IKHHODUJTQSREF
**1844**	-5.9211	292.1542	3.368793	Linear polyketides	Fatty acids	LJFWHSLCSLLNMJ
**2105**	-6.0902	312.1801	3.724945	Fatty amides	Fatty acids	XADABARFXGVGAG
**2030**	-6.2515	964.5263	3.616564	unknown	unknown	REFUJNWZICDHTR
**4504**	-6.2768	660.4081	6.40727	Fatty acyl glycosides	Polyketides	TZXXJSXEDDLWPB
**3553**	-6.2996	739.5338	5.523957	Cyclic polyketides	unknown	unknown
**1621**	-6.3617	315.1679	3.038101	unknown	unknown	WSFQKSIBZODGPB
**774**	-6.3645	357.1448	1.628669	Anthranilic acid alkaloids	Alkaloids	WVPWAWQSZCUQGO
**2466**	-6.4912	357.1814	4.236066	Tryptophan alkaloids	Alkaloids	KRKNYBCHXYNGOX
**4492**	-6.6649	358.1837	6.406226	Macrolides	Terpenoids	XCFSBOSFMAOQAL
**3036**	-6.8078	355.2358	4.903929	Macrolides	Amino acids and Peptides	XZORUNHMSUAKIS
**1549**	-7.0171	284.1496	2.911744	Fatty amides	Fatty acids	RRUCJRYDVISARM
**3071**	-7.0407	755.4445	4.918877	Fatty Acids and Conjugates	Amino acids and Peptides	HENXXJCYASTLGZ
**2278**	-7.0514	992.5574	3.965407		unknown	GWBOEEVOSDBARW
**3044**	-7.0801	348.2741	4.904153	Fatty Acids and Conjugates	Fatty acids	UDMBCSSLTHHNCD
**3048**	-7.1909	683.4728	4.900607	unknown	unknown	QWJFFNXSWQZKKL
**2493**	-7.4858	649.3942	4.258302	unknown	unknown	JZPVHPRJAZUVLI
**4037**	-7.5463	658.3196	5.979505	Lignans	Terpenoids	RUVURMKOCXQXKM
**4182**	-7.8019	649.3263	6.097589	Glycerolipids	Polyketides	QJEJLWMIUCXQMB
**2724**	-8.5579	1155.625	4.556579	unknown	unknown	TXQJQQFSZZXWBI
**3133**	-8.5885	329.2308	5.007749	Tryptophan alkaloids	Fatty acids	MKYUCBXUUSZMQB
**1746**	-8.6766	530.2971	3.180274	Oligopeptides	Amino acids and Peptides	YOGYBVYVKDQUPM
**3057**	-8.7787	705.4525	4.909996	Pseudoalkaloids	unknown	RKSUOLKPWPIKPM

**1769**	-8.8683	297.2177	3.233405	Small peptides	Fatty acids	NXLWUGHGMALROJ
**4036**	-8.9377	663.274	5.972192	Fatty Acids and Conjugates	Shikimates and Phenylpropanoids	CLHUYSRBASDRTG
**3123**	-8.9422	351.2138	5.006911	Polyethers	Fatty acids	ZSCYVHXHBMERNH
**4281**	-9.1374	380.3541	6.191017	Macrolides	Fatty acids	VDYDCVUWILIYQF
**3434**	-9.7211	429.2406	5.398778	Guanidine alkaloids	Alkaloids	BJIBMMAMDZHYDD
**3449**	-9.8148	601.3419	5.430261	Fatty acyl glycosides	unknown	XLQIVSCKAHVDRB
**1520**	-10.056	518.298	2.875767	Small peptides	Amino acids and Peptides	AAABMNXUOFPYQK
**2366**	-10.2	927.3637	4.114483	unknown	unknown	ZEEKBRYJSXBBCB
**4474**	-10.235	638.4261	6.346648	Linear polyketides	Polyketides	QMNSCQKHTRAKTJ
**2884**	-10.237	1155.625	4.731846	unknown	unknown	AFMABPRBXOCKJG
**2849**	-10.416	551.3319	4.685553	Oligopeptides	Polyketides	KOPYOPABTFQTRP
**2305**	-11.434	320.1829	3.997917	Linear polyketides	Alkaloids	FMGBNISRFNDECK
**3623**	-11.813	379.2455	5.585028	Fatty esters	Fatty acids	NCIVVGQMYOZDSP
**2301**	-11.896	298.2007	4.001052	Fatty amides	Fatty acids	PHSRRHGYXQCRPU
**3616**	-11.99	357.2637	5.580067	Fatty Acids and Conjugates	Fatty acids	RXGMJLUUKIBYON
**1735**	-15.449	270.1696	3.166518	Fatty amides	Fatty acids	KYGIKEQVUKTKRR

Table S3. Features with differential abundance in PA14Δ4mex vs PA14 WT and EPs overexpressors vs PA14Δ4mex – GC-MS

Predicted coumpound	Rentention time (min)	Match Factor (%)	NPC Pathway	NPC Superclass	NPC Class	log2(FC)	P-value	EP overexpressor
**Increased features (log2FC >1 or < -1 and a P-value ≤ 0.05) in EPs overexpressors compared to PA14Δ4mex**								
**1-(1,3-Oxazol-2-yl)ethan-1-one**	13.39489456	75.7484954	Unknwown	Unknwown	Unknwown	5.8634	0.00167	CDJ, PQE
**d-Proline, N-methoxycarbonyl-, heptyl ester**	14.98164656	78.37580761	Amino acids and Peptides	Small Peptides	Unknwown	5.6805	0.0042625	EFN, ABM, CDJ, PQE
**l-Norvaline, n-propargyloxycarbonyl-, pentyl ester**	14.93399338	78.86640291	Fatty acids	Fatty esters	Wax monoesters	2.4259	0.025057	CDJ
**l-Alanine, N-(2-trifluoromethylbenzoyl)-, dodecyl ester**	21.53954662	77.39857598	Unknwown	Unknwown	Unknwown	2.0657	0.041945	EFN
**Alanine, N-methyl-N-(2-methoxyethoxycarbonyl)-, dodecyl ester**	28.9577739	78.37549269	Fatty acids	Fatty esters	Wax monoesters	1.1695	0.018362	PQE
**Increased or decreased features (log2FC >1 or < -1 and a P-value ≤ 0.05) in PA14Δ4mex vs PA14 wild type**								
**Propanoic acid, 2-methyl-, propyl ester**	9.356240337	87.06495201	Fatty acids	Fatty acids and conjugates	Branched fatty acids	-3.9633	0.023432	*NA*
**Octanoic acid, 3-hydroxy-, methyl ester**	5.947940125	96.04446418	Fatty acids	Fatty acids and conjugates	Fatty esters	-3.8988	0.0026214	*NA*
**2-Butanol, 3-(2,2-dimethylpropoxy)-**	7.041511171	77.46146289	Fatty acids	Fatty acyls	Fatty alcohols	-3.4865	0.024534	*NA*
**cis-10-Nonadecenoic acid, methyl ester**	21.34896788	93.21047198	Fatty acids	Fatty esters	Wax monoesters	-3.4014	0.00095095	*NA*
**Acetyl iodide**	8.626896729	70.84489504	Unknown	Unknown	Unknown	-2.956	0.03187	*NA*
**Butanal butyl isoamyl acetal**	25.47398977	70.48601184	Fatty acids	Unknown	Unknown	-2.8577	0.020975	*NA*
**cis-10-Heptadecenoic acid, methyl ester**	17.79446779	83.27144488	Fatty acids	Fatty esters	Wax monoesters	-2.3997	0.0039815	*NA*
**Decanoic acid, 3-hydroxy-, methyl ester**	8.327474035	95.06972588	Fatty acids	Fatty acids and conjugates	Fatty esters	-2.0082	0.038271	*NA*
**Benzoic acid, 2-(isopropyl)amino-, methyl ester**	8.232292243	75.49069248	Alkaloids	Pseudoalkaloids	Anthranillic acid alkaloids	1.2167	0.041429	*NA*
**1-Hydroxyphenazine**	15.54292252	71.51768933	Alkaloids	Anthranillic acid alkaloids	Phenazine alkaloids	4.0915	0.015921	*NA*
**Phenazine, 1-methoxy-**	18.38674793	73.86611949	Alkaloids	Anthranillic acid alkaloids	Phenazine alkaloids	5.4146	0.0021368	*NA*

Table S4. Verification of feature ID identity using commercially available standards. All standards were injected at 1mg/ml. UHPLC-MS

Pseudomonas Quinolone signal (2-Heptyl-3-hydroxy-4-quinolone) Exact mass = 259.157229 g/mol

ID	m/z		retention time (RT) in min	Peak area in the standard
1954	260.1641	M + H	3.473661	1.17E+09

HHQ (2-heptylquinolin-4(1H)-one) Exact mass = 243.162314293 g/mol

ID	m/z		retention time (RT) in min	Peak area in the standard
1659	244.1695	M + H	3.0952892	1.45E+09

Pyochelin Exact mass = 324.06023472 g/mol

ID	m/z		retention time (RT) in min	Peak area in the standard
1509	325.0672	M + H	2.8904462	5.32E+08
1699	325.0673	M + H	3.1359556	4.93E+08
1372	325.0673	M + H	2.6265237	3.30E+08
1816	325.0675	M + H	3.2570164	1.89E+08

C4-HSL (N-butanoyl-L-homoserine lactone) Exact mass = 171.08954328 g/mol

ID	m/z		retention time (RT) in min	Peak area in the standard
327	172.0968	M + H	0.8151582	4.26E+06

Table S5. First fifty highest peaks in the QC samples (average of triplicates) – UHPLC-MS

ID	Predicted compound	Average peak intensity in QC
**3273**	Rhamnolipid	81177202.5
**2145**	2-nonyl-1H-quinolin-4-one	66475309
**1954**	PQS	56498153
**2132**	2-[(E)-non-1-enyl]-1H-quinolin-4-one	56058129
**2256**	1-hydroxy-2-nonylquinolin-4-one	36294325.5
**1783**	2-heptyl-3-hydroxy-3H-quinolin-4-one	34296427.5
**2505**	N-[(3,4-dimethoxyphenyl)methyl]-2-(4-methoxyphenyl)-N-methylethanamine	29861350.5
**2388**	2-undec-3-enyl-1H-quinolin-4-one	27586527.5
**3241**	3-(3-hydroxydecanoyloxy)decanoic acid	26344707
**3832**	[(2R)-2-hydroxy-3-[(Z)-icos-13-enoyl]oxypropyl] 2-(trimethylazaniumyl)ethyl phosphate	25522233
**1509**	Pyochelin	23217392
**3598**	Spirodienal B	22720840
**2999**	Kijimicin	21599482.5
**2111**	2-(8-oxononyl)-1H-quinolin-4-one	19102341.5
**3274**	(E)-3-[(1S,2S,4aS,6S,7R,8S,8aR)-6,7-dihydroxy-3,4a,8-trimethyl-2-[(E,5R,7S,8R,9R)-5,7,9-trihydroxy-8,10-dimethylundec-2-en-2-yl]-2,5,6,7,8,8a-hexahydro-1H-naphthalen-1-yl]-2-methylprop-2-enoic acid	17265733.5
**292**	Pyocyanin	14876033.75
**2472**	1-hydroxy-2-undec-4-enylquinolin-4-one	14371357.5
**2694**	2-undecyl-4-quinolone	12672796.75
**2147**	Stanolone	12591515
**2659**	2-undec-3-enyl-1H-quinolin-4-one	11724788.25
**2131**	(3R,4S,4aS,5S,8R,8aS)-4,8-dihydroxy-5-(2-hydroxypropan-2-yl)-3,8-dimethyl-1,3,4,4a,5,6,7,8a-octahydronaphthalen-2-one	11432515.5
**3514**	[(2E,4Z,6Z,8E,10E,12Z,14E)-2-[2-(4-hydroxy-2,6,6-trimethylcyclohexen-1-yl)ethynyl]-17-(1-hydroxy-2,2,6-trimethyl-4-oxocyclohexyl)-6,11,15-trimethyl-16-oxoheptadeca-2,4,6,8,10,12,14-heptaenyl] hexanoate	10744763.5
**1699**	Pyochelin	10063726.63
**3252**	3-hydroxydecanoic acid	8480178.75
**3247**	mono-rhamnolipid	8345841.25
**3830**	Spirodienal	8106307.75
**3248**	Unknown	7727843.875
**1335**	phenazine-1-carboxylic acid	7616861.45

**3264**	Unknown	7597638.5
**2576**	Belladine	7282557
**1917**	Elacomine	7129920.875
**2733**	[(2R)-2-[(Z)-hexadec-9-enoyl]oxy-3-hydroxypropyl] 2-(trimethylazaniumyl)ethyl phosphate	6827318.875
**2263**	(2R)-2-[(2S)-2-hydroxy-6-methylhept-5-en-2-yl]-2,3-dihydro-1H-indole-5-carboxamide	6691218
**2716**	1-hydroxy-2-undec-4-enylquinolin-4-one	6629732.625
**1271**	phenazine-1-carboxylic acid	6566872.875
**432**	quinolin-4-ylmethanol	6552653.25
**3828**	3-(3-hydroxydodecanoyloxy)decanoic acid	6393531.875
**1659**	2-heptyl-1H-quinolin-4-one	6153403.375
**3249**	Limonene oxide	5990816.375
**2464**	(2R)-2-[(2S)-2-hydroxy-6-methylhept-5-en-2-yl]-2,3-dihydro-1H-indole-5-carboxamide	5907046.375
**2384**	(4S,5S)-5-[(1S,2R,4R)-4-butyl-2-ethyl-2-hydroxycyclopentyl]-5-ethyl-4-hydroxyoxolan-2-one	5801807.625
**3605**	15-hydroxy-10,12,14-trimethyl-7-(7,9,11-trihydroxy-8,10-dimethyldodec-4-en-2-yl)-8,17-dioxabicyclo[14.1.0]heptadeca-4,10,12-trien-9-one	5704259.25
**1786**	(3S,6S)-3-benzyl-6-(2-methylpropyl)piperazine-2,5-dione	5451646.175
**3261**	(E,7S)-7-methoxy-N-(2-phenylethyl)tetradec-4-enamide	5199323.5
**2876**	2-[(Z)-dodec-5-enyl]-1-methylquinolin-4-one	5079902.125
**3246**	Unknown	5071765.875
**2771**	2-Undecyl-4-quinolinol 1-oxide	4778000
**3513**	[(2R)-3-[10-(2-hexylcyclopropyl)decanoyloxy]-2-hydroxypropyl] 2-(trimethylazaniumyl)ethyl phosphate	4728045.175
**3586**	[(2S)-2-hydroxy-3-[(Z)-2-methylbut-2-enoyl]oxypropyl] 12-methyltridecanoate	4561868.875
**3266**	Kijimicin	4499013.875

Table S6. Features predicted to be homoserine lactones (n= 23) in the UHPLC-MS analysis

Feature ID	Carbons chain length	Log2(FC) PA14Δ4mex vs PA14	m/z	RT	NPC Pathway	Predicted compound
327	4	0	172.0968	0.815158	Fatty acids	C4-HSL
1100	8	0	242.1388	2.176544	Fatty acids	N-(3-Oxooctanoyl)-L-homoserine lactone
1108	8	0	264.1205	2.180272	unknown	NA
**1735**	**10**	**-15.449**	**270.1696**	**3.166518**	**Fatty acids**	**3-oxo-N-(2-oxooxolan-3-yl)decanamide**
1736	10	0	292.1528	3.215087	unknown	NA
1741	10	0	308.126	3.175227	unknown	(3,4-dimethoxyphenyl)-N-[(4-methylphenyl)methyl]carboxamide
**2301**	**10**	**-11.896**	**298.2007**	**4.001052**	**Fatty acids**	**N-(3-Oxododecanoyl)-L-homoserine lactone**
**2305**	**10**	**-11.434**	**320.1829**	**3.997917**	**Alkaloids**	**NA**
2306	10	0	336.1571	4.004273	unknown	NA
2307	10	0	617.3785	4.004273	unknown	NA
2544	10	0	316.2483	4.33911	Fatty acids	NA
2015	11	0	284.1853	3.596734	Fatty acids	3-oxo-C11-HSL
2016	11	0	306.1675	3.596734	unknown	NA
2558	11	0	346.2002	4.289031	Alkaloids	NA
2562	11	0	306.2063	4.35744	Fatty acids	NA
2563	11	0	324.2166	4.329101	Fatty acids	3-oxo-C11-HSL
**2553**	**12**	**-4.5759**	**298.2011**	**4.346155**	**Fatty acids**	**N-(3-Oxododecanoyl)-L-homoserine lactone**
**1549**	**13**	**-7.0171**	**284.1496**	**2.911744**	**Fatty acids**	**3-oxo-C13-HSL**
**2105**	**13**	**-6.0902**	**312.1801**	**3.724945**	**Fatty acids**	**3-oxo-C13-HSL**
174	NA	0	144.1019	0.667265	Fatty acids	NA
325	NA	0	144.1019	0.800219	Fatty acids	NA
333	NA	0	186.1122	0.782869	Fatty acids	NA
368	NA	0	204.1229	0.877613	Fatty acids	methyl 2-(2-hydroxypropanoylamino)-3-methylbutanoate
