# Supplementary material for: Multidrug efflux pumps of Pseudomonas aeruginosa show selectivity for their natural substrates
Source: Front Microbiol. 2025 Jan 9;15:1512472. doi: 10.3389/fmicb.2024.1512472 (PMC11754269; doi:10.3389/fmicb.2024.1512472)
Supplement: Supplementary file 2 [file Data_Sheet_2.pdf]

Supplementary figures, Mazza et al.

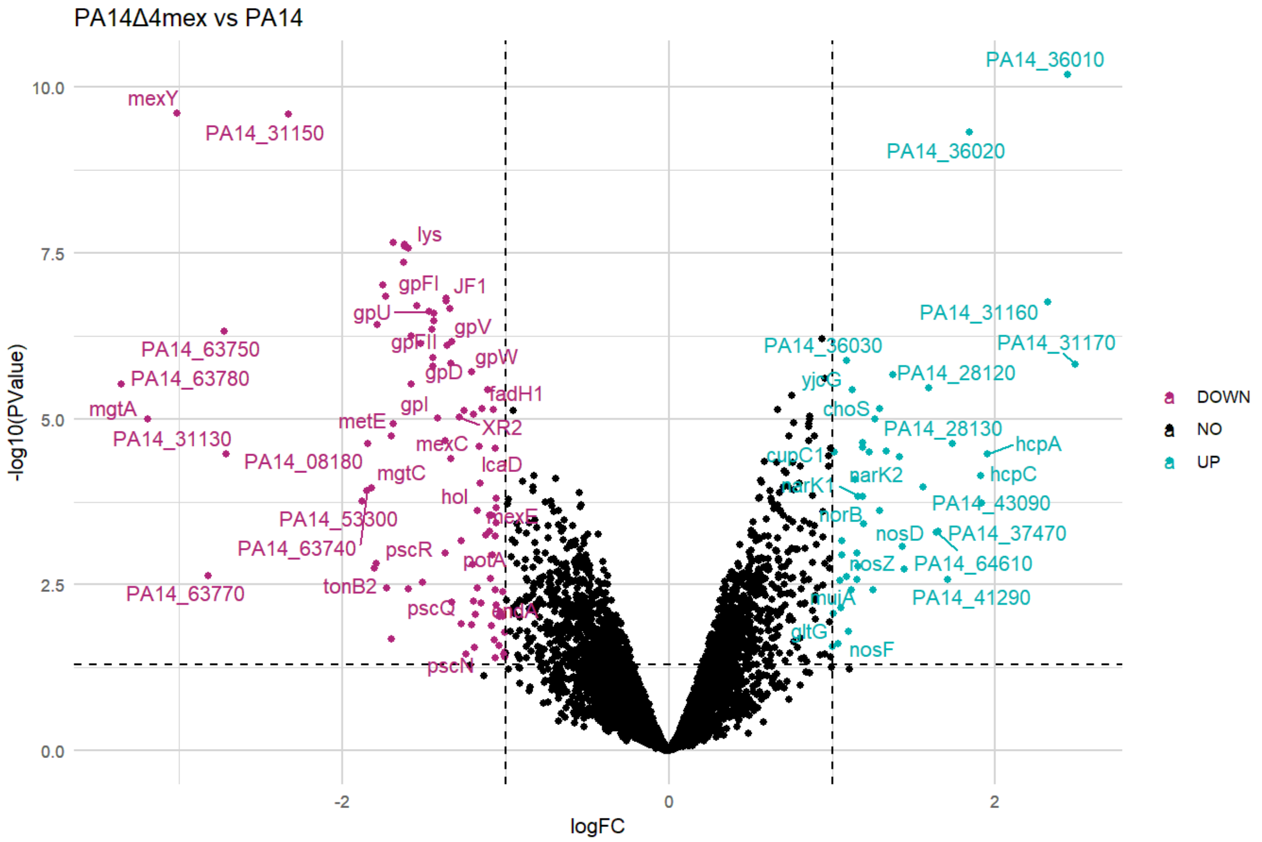

Figure S1. **RNA sequencing of PA14Δ4mex vs PA14.** Genes with significantly reduced expression are shown in magenta and genes with significantly increased expression are shown in blue. Genes without significantly changed expression are shown in black. Details can be found in Table S1.

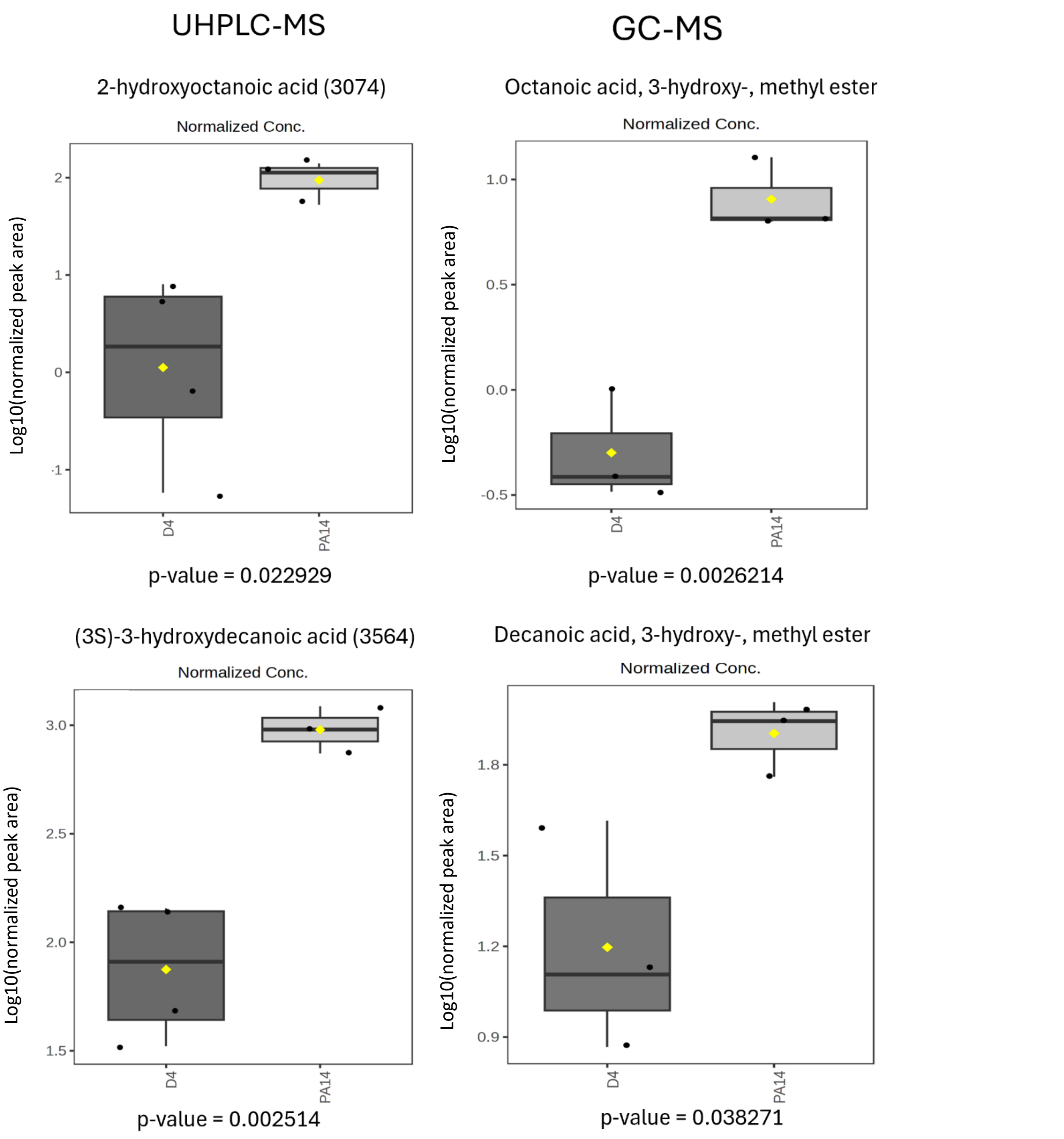

Figure S2. Metabolites with decreased abundance in PA14Δ4mex exo-metabolome compared to PA14 exo-metabolome. Predictions are based on UHPLC-MS analyses and Sirius annotation tool (left panels) and GC-MS analyses and comparison to NIST library (right panels). Relative concentrations are expressed as log10 of normalized peak areas.

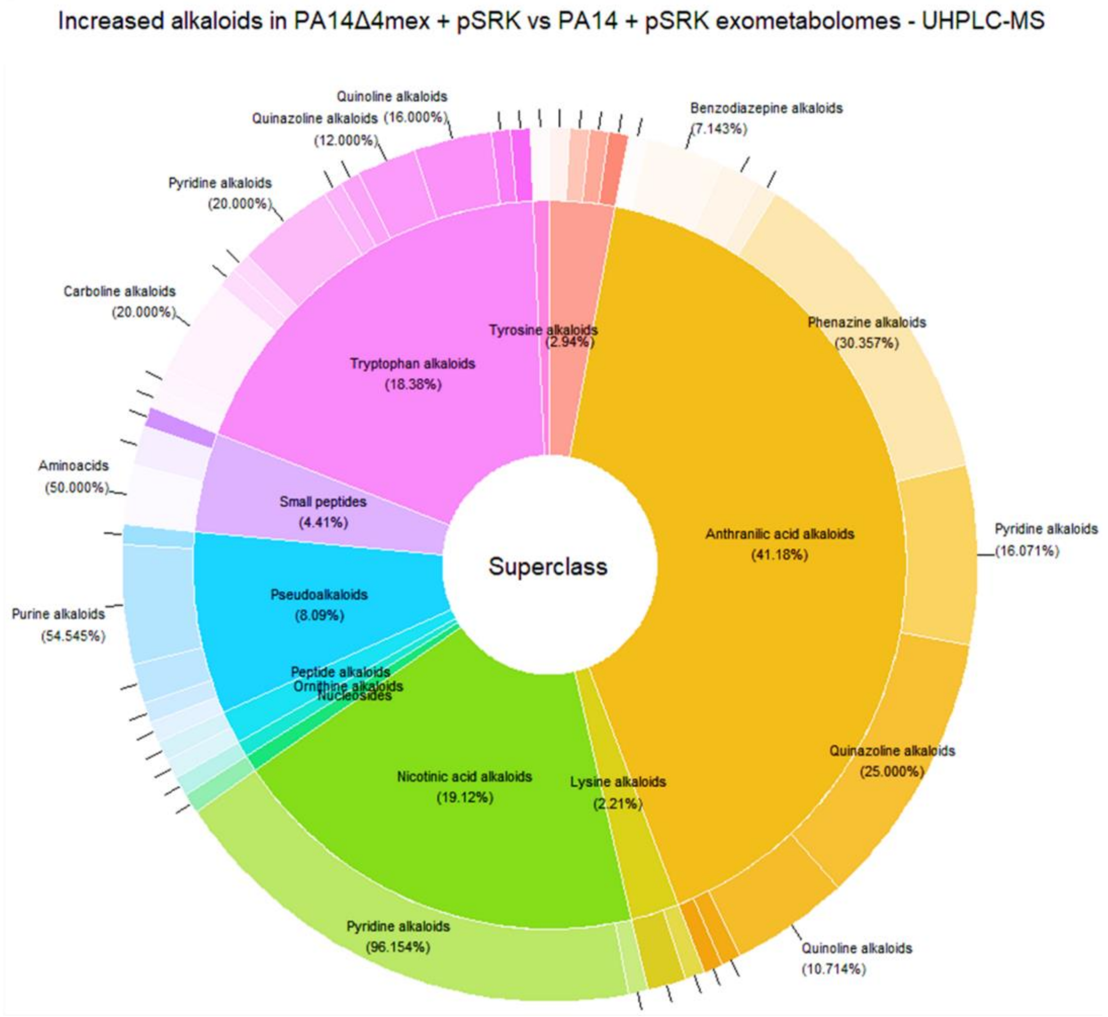

Figure S3. Alkaloids increased in UHPLC-MS analysis (log2FC ≥ 1 and p-value < 0.05) of PA14Δ4mex + pSRKGm vs PA14+ pSRKGm exo-metabolomes. Classification was established by NPClassifier[41] : NPC superclass (inner ring) and class (outer ring). The most represented features include anthranilic acid alkaloids, specifically pyridine alkaloids, phenazine alkaloids and quinazoline alkaloids.

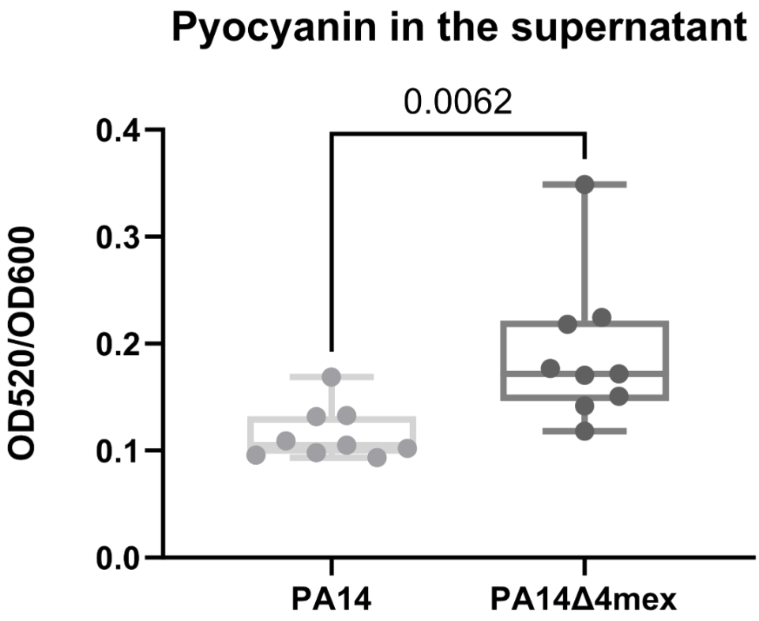

Figure S4. **Pyocyanin in the supernatant of PA14 wild type and PA14Δ4mex.** Pyocyanin was extracted by chloroform and measured after acidification in the aqueous phase at OD520. Absorption was normalized to cell density (OD600). Measures were repeated on three independent occasions in triplicates.

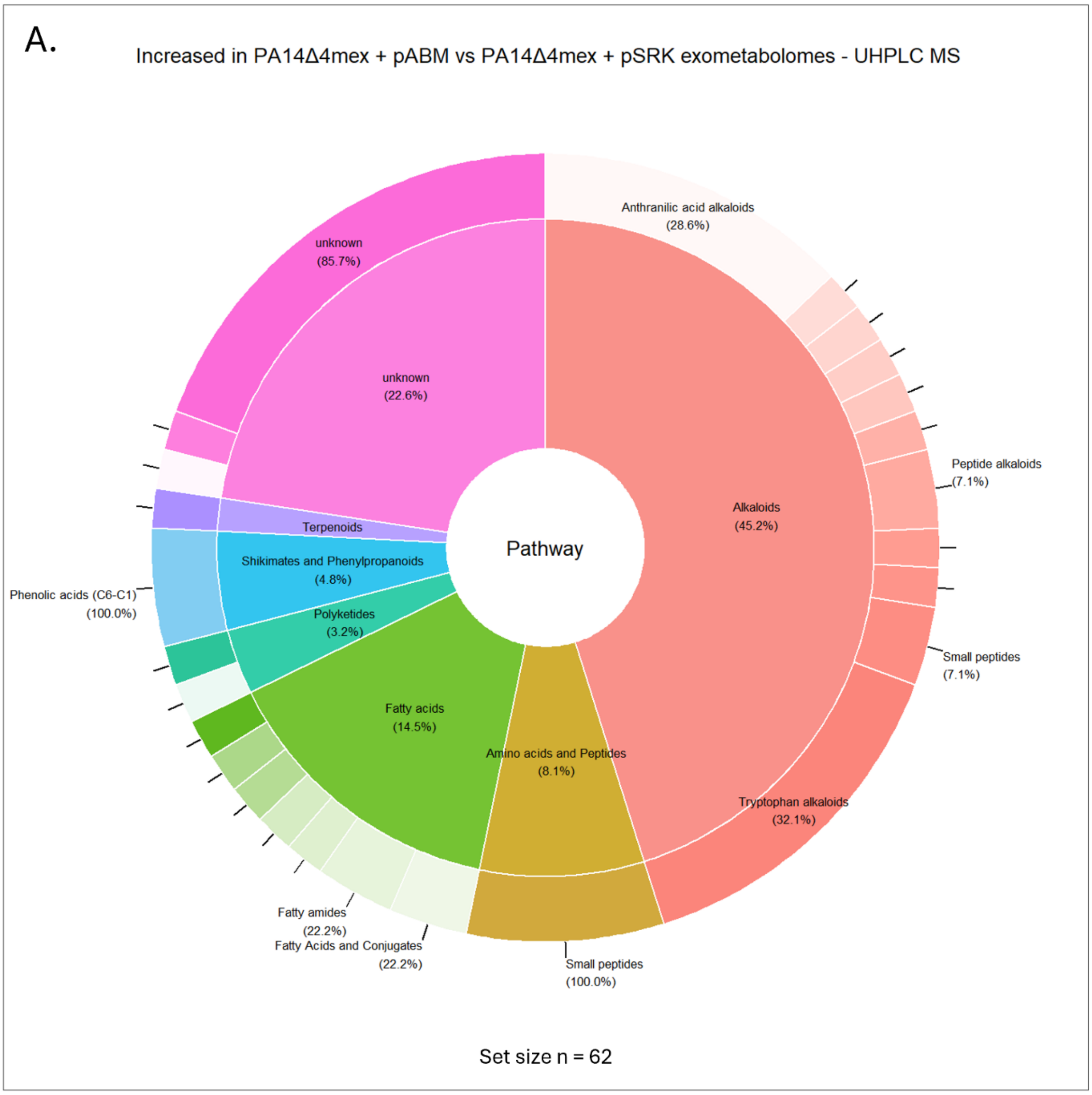
A.
Increased in PA14Δ4mex + pABM vs PA14Δ4mex + pSRK exometabolomes - UHPLC MS
Anthranilic acid alkaloids
(28.6%)
unknown
(85.7%)
unknown
(22.6%)
Peptide alkaloids
(7.1%)
Alkaloids
(45.2%)
Terpenoids
Pathway
Shikimates and Phenylpropanoids
(4.8%)
Phenolic acids (C6-C1)
(100.0%)
Polyketides
(3.2%)
Small peptides
(7.1%)
Fatty acids
(14.5%)
Amino acids and Peptides
(8.1%)
Tryptophan alkaloids
(32.1%)
Fatty amides
(22.2%)
Fatty Acids and Conjugates
(22.2%)
Small peptides
(100.0%)
Set size n = 62

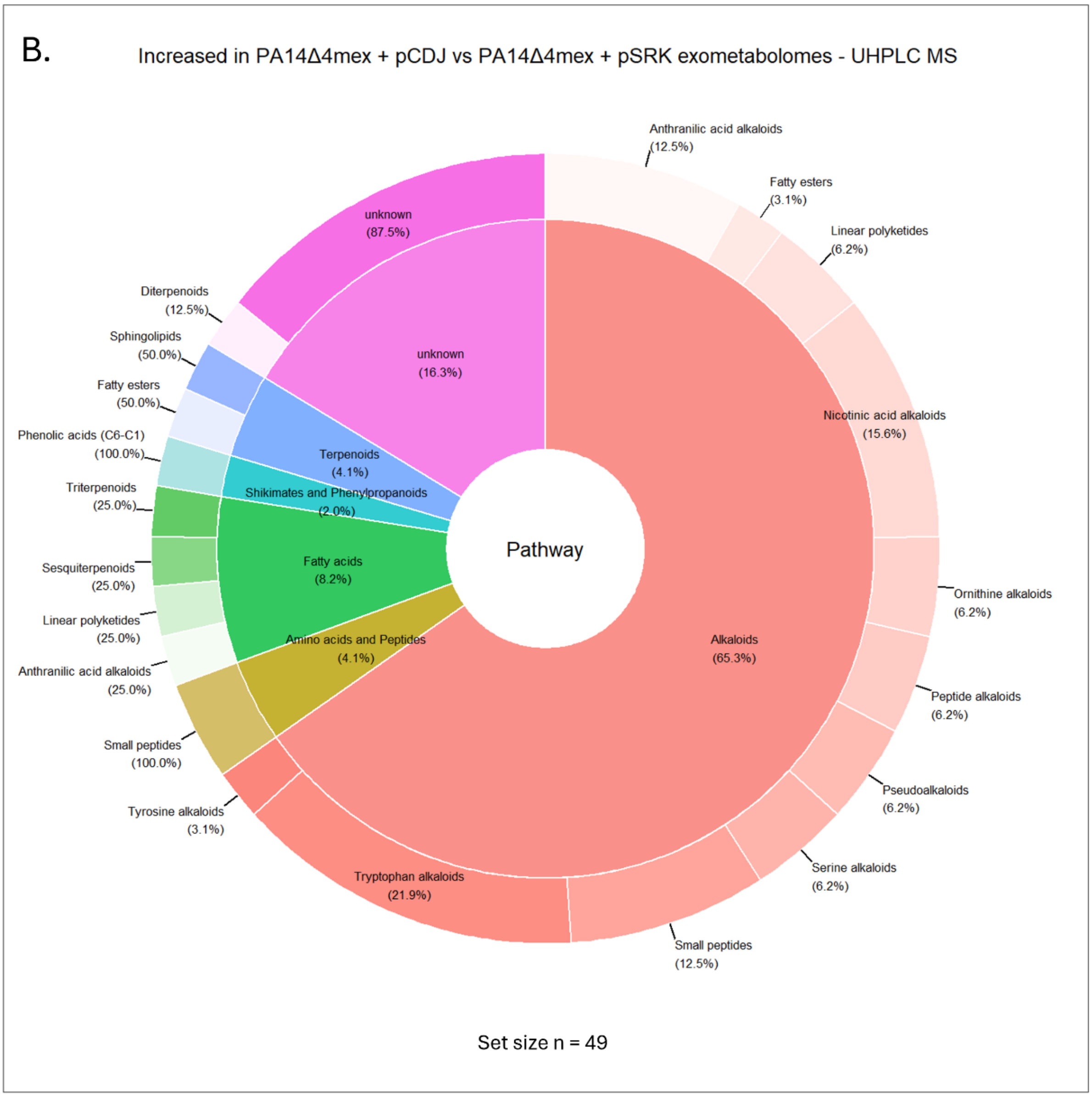

B.
Increased in PA14Δ4mex + pCDJ vs PA14Δ4mex + pSRK exometabolomes - UHPLC MS
Pathway
unknown (87.5%)
unknown (16.3%)
Anthranilic acid alkaloids (12.5%)
Fatty esters (3.1%)
Linear polyketides (6.2%)
Nicotinic acid alkaloids (15.6%)
Ornithine alkaloids (6.2%)
Peptide alkaloids (6.2%)
Pseudoalkaloids (6.2%)
Serine alkaloids (6.2%)
Small peptides (12.5%)
Tryptophan alkaloids (21.9%)
Alkaloids (65.3%)
Tyrosine alkaloids (3.1%)
Small peptides (100.0%)
Amino acids and Peptides (4.1%)
Anthranilic acid alkaloids (25.0%)
Linear polyketides (25.0%)
Sesquiterpenoids (25.0%)
Fatty acids (8.2%)
Triterpenoids (25.0%)
Phenolic acids (C6-C1) (100.0%)
Shikimates and Phenylpropanoids (2.0%)
Fatty esters (50.0%)
Sphingolipids (50.0%)
Terpenoids (4.1%)
Diterpenoids (12.5%)
Set size n = 49

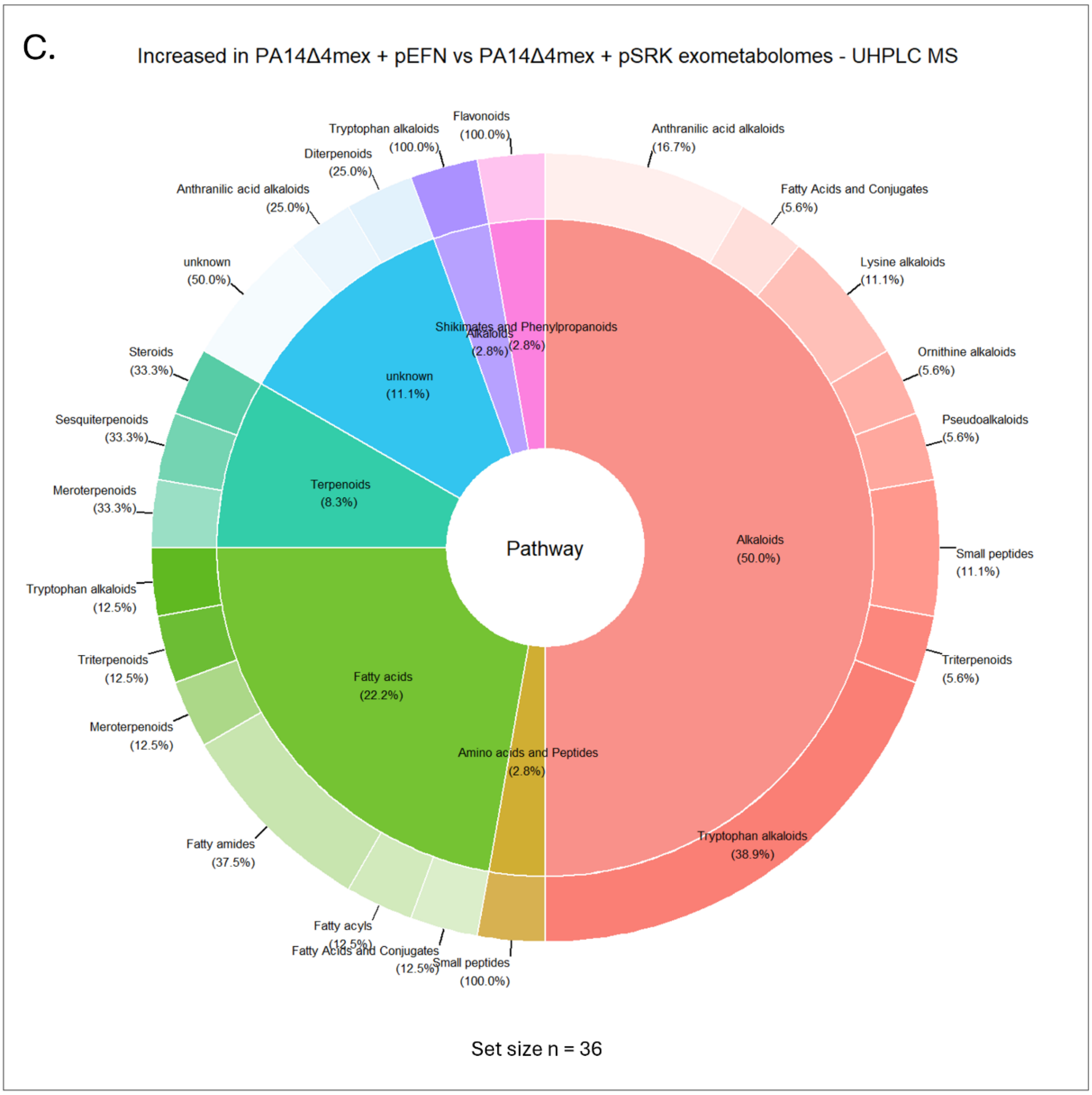
C.
Increased in PA14Δ4mex + pEFN vs PA14Δ4mex + pSRK exometabolomes - UHPLC MS
Pathway
Alkaloids (50.0%)
Fatty acids (22.2%)
Terpenoids (8.3%)
unknown (11.1%)
Shikimates and Phenylpropanoids (2.8%)
Alkaloids (2.8%)
Amino acids and Peptides (2.8%)
Tryptophan alkaloids (38.9%)
Anthranilic acid alkaloids (16.7%)
Fatty Acids and Conjugates (5.6%)
Lysine alkaloids (11.1%)
Ornithine alkaloids (5.6%)
Pseudoalkaloids (5.6%)
Small peptides (11.1%)
Triterpenoids (5.6%)
Flavonoids (100.0%)
Tryptophan alkaloids (100.0%)
Diterpenoids (25.0%)
Anthranilic acid alkaloids (25.0%)
unknown (50.0%)
Steroids (33.3%)
Sesquiterpenoids (33.3%)
Meroterpenoids (33.3%)
Tryptophan alkaloids (12.5%)
Triterpenoids (12.5%)
Meroterpenoids (12.5%)
Fatty amides (37.5%)
Fatty acyls (12.5%)
Fatty Acids and Conjugates (12.5%)
Small peptides (100.0%)
Set size n = 36

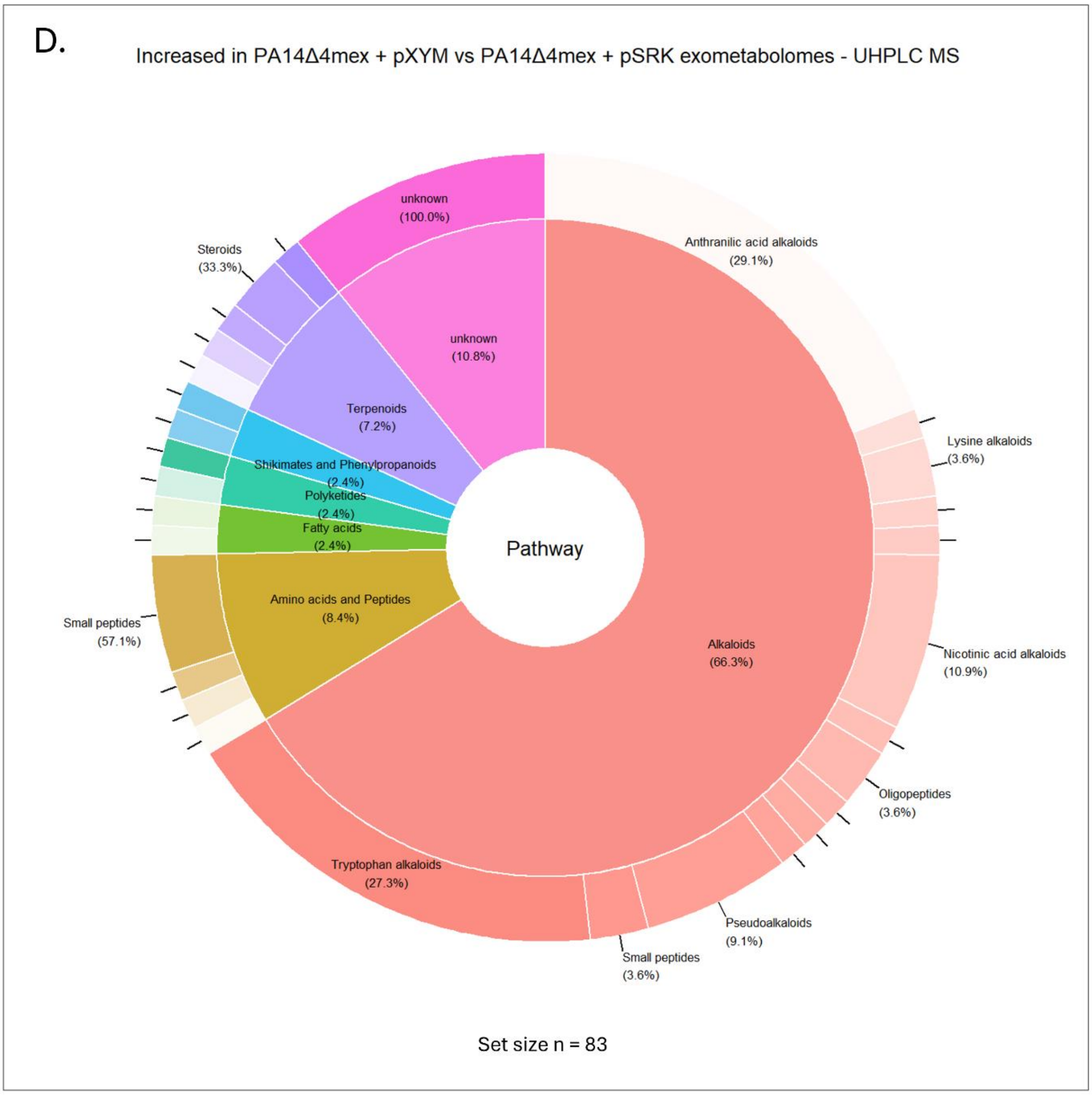
D.
Increased in PA14Δ4mex + pXYM vs PA14Δ4mex + pSRK exometabolomes - UHPLC MS
unknown
(100.0%)
Steroids
(33.3%)
unknown
(10.8%)
Terpenoids
(7.2%)
Shikimates and Phenylpropanoids
(2.4%)
Polyketides
(2.4%)
Fatty acids
(2.4%)
Amino acids and Peptides
(8.4%)
Small peptides
(57.1%)
Pathway
Anthranilic acid alkaloids
(29.1%)
Lysine alkaloids
(3.6%)
Alkaloids
(66.3%)
Nicotinic acid alkaloids
(10.9%)
Oligopeptides
(3.6%)
Tryptophan alkaloids
(27.3%)
Pseudoalkaloids
(9.1%)
Small peptides
(3.6%)
Set size n = 83

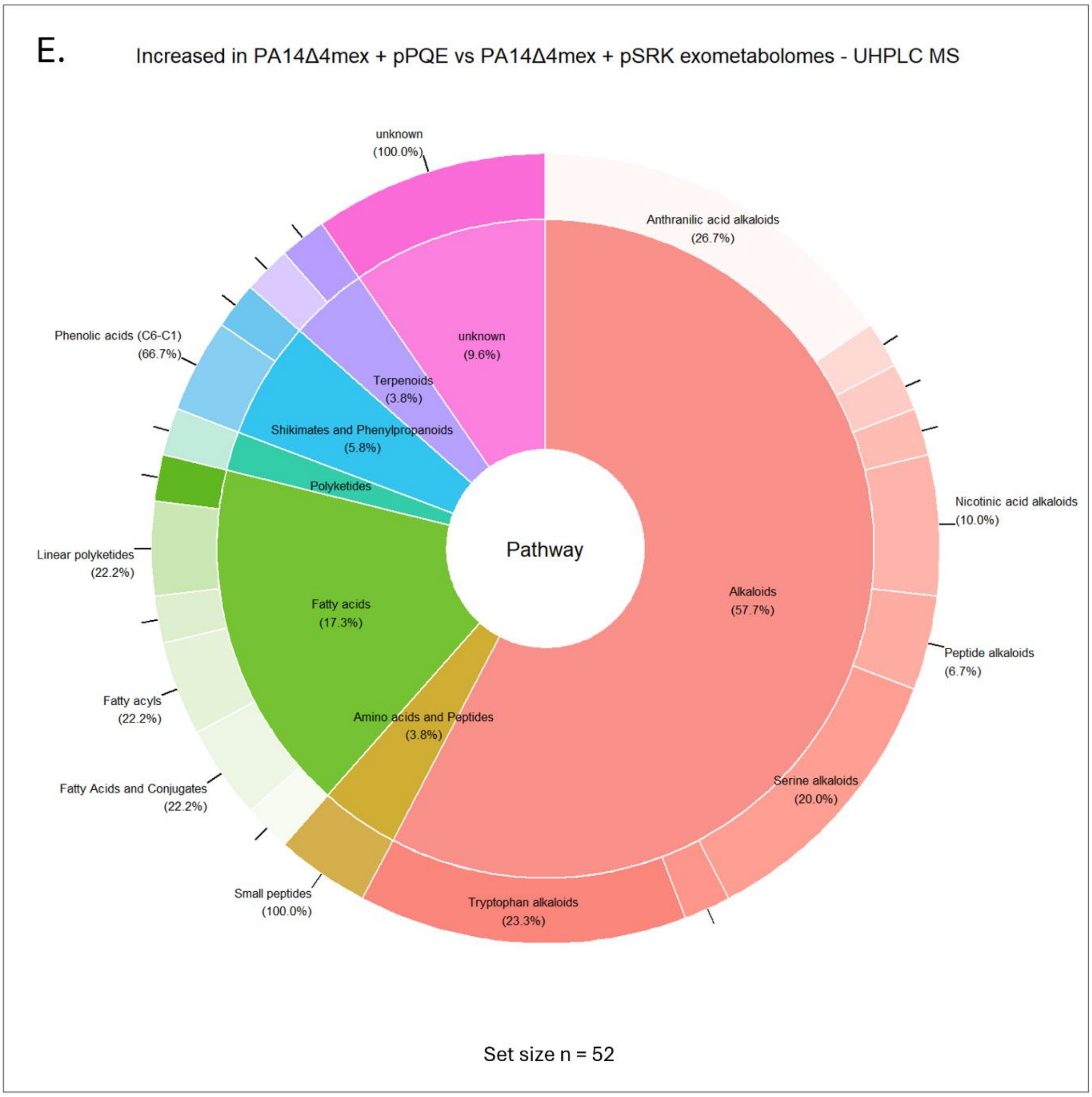

Figure S5. Increased features of interest ($log_2FC \geq 1$ and P-value ≤ 0.05) identified by UHPLC-MS analysis in exo-metabolomes of EP overexpressors compared to PA14Δ4mex (pSRKGm). The classification established by NPClassifier[34] shows the NPC pathways (inner ring) and superclasses (outer ring) for exo-metabolomes from strains overexpressing **A.** MexAB-OprM **B.** MexCD-OprJ **C.** MexEF-OprN **D.** MexXY-OprM and **E.** MexPQ-OpmE EPs.

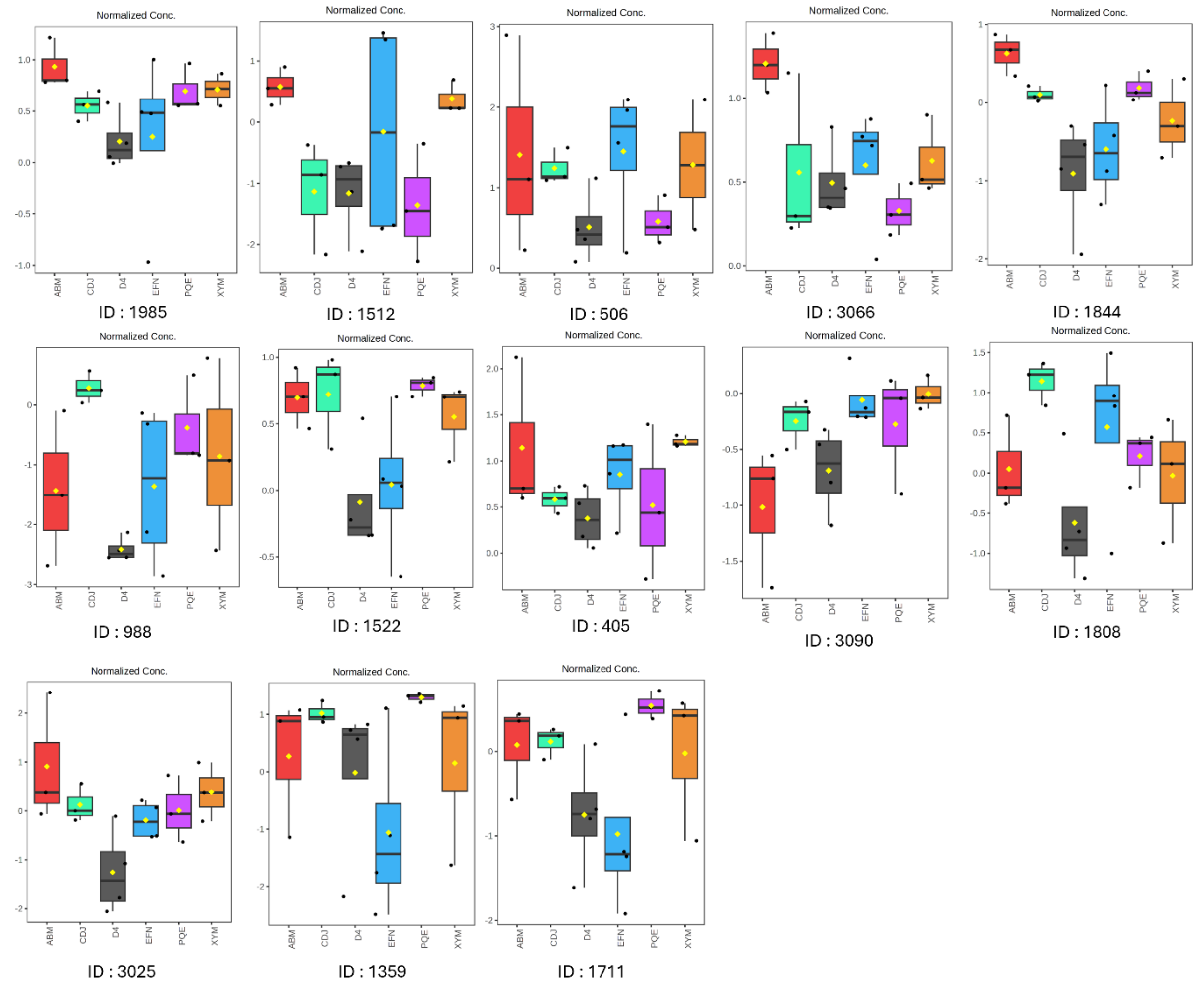

Figure S6. Normalized concentration (peak area of feature/peak area of internal standard and transformed to log10) of predicted compounds presented in Fig 4. Predictions were made using the Sirius annotation tool. Features “of interest” are defined by log2(FC) ≥ 1 and p-value ≤ 0.05 (Student T-test) in the UHPLC-MS analyses of exo-metabolomes from biological triplicates. PA14 = PA14 + pSRKGm. Δ4 = PA14Δ4mex + pSRKGm. ABM = PA14Δ4mex + pmexAB-oprM. CDJ = PA14Δ4mex + pmexCD-oprJ. EFN = PA14Δ4mex + pmexEF-oprN. XYM = PA14Δ4mex + pmexXY-oprM. PQE = PA14Δ4mex + pmexPQ-opmE.

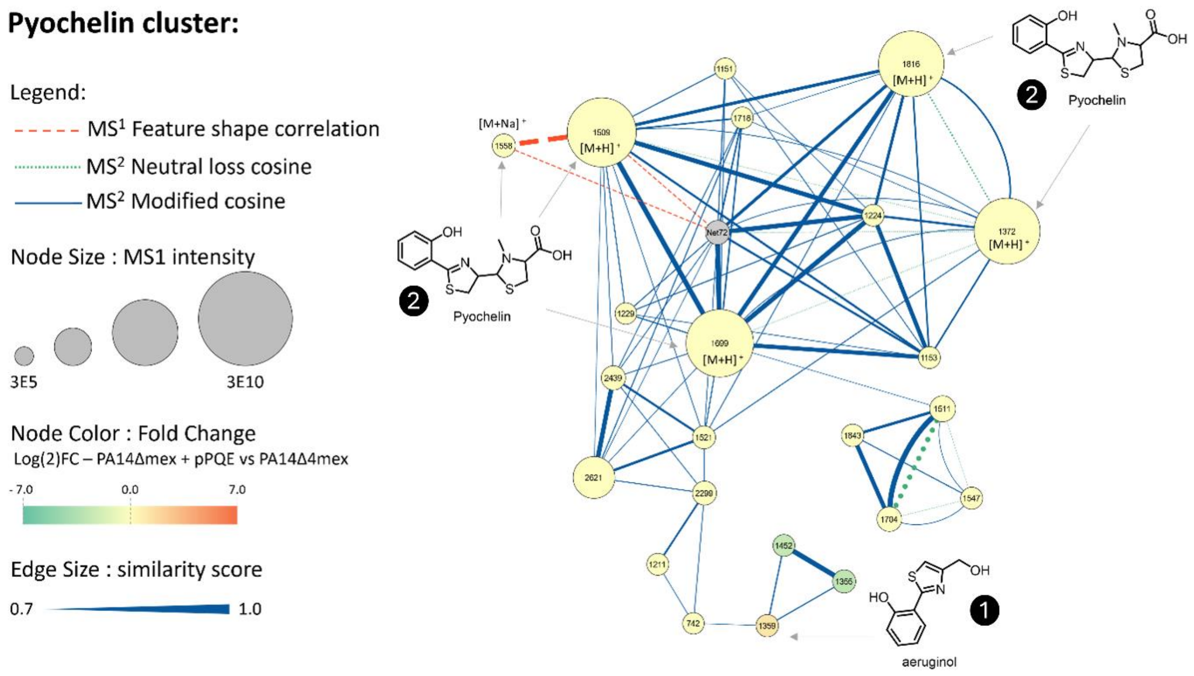

Figure S7. Metabolomic analysis and molecular networks of pyochelin cluster from PA14 exo-metabolomes. The molecular network was generated directly with MzMine 4.0. The identified features are represented as nodes (circles), showing their ID in the node centers together with the adduct when identified. The size of each nodes is proportional to the MS[1] intensity of the feature. Nodes are colored based on their relative abundance in the MexPQ-OpmE overexpressor exo-metabolome compared to PA14Δ4mex exo-metabolome. Nodes are linked with different relationships and width's edges are proportional to the similarity. Putative structures are based on SIRIUS annotations. Pyochelin was confirmed by injection of a commercially available standard.
